# Synthesis of depressin, cryptomeridiol and 4-*epi*-cryptomeridiol enabled by a terpenoid chiral pool-producing platform

**DOI:** 10.3762/bjoc.22.53

**Published:** 2026-05-05

**Authors:** Yao Kong, Tao Wang, Chen Wang, Pengcheng Zhang, Yuanning Liu, Kaibiao Wang, Fen Liu, Hongli Jia, Zhengren Xu

**Affiliations:** 1 State Key Laboratory of Natural and Biomimetic Drugs, Department of Integration of Chinese and Western Medicine, School of Basic Medical Sciences, Peking University, Beijing 100191, China; 2 Beijing Key Laboratory of Carbohydrate Intelligent Manufacture and Functional Applications, School of Pharmaceutical Sciences, Peking University, Beijing 100191, China

**Keywords:** chemoenzymatic synthesis, chiral pool, isopentenol utilization pathway, terpene cyclases

## Abstract

Depressin (**1**) is a soft coral-derived diterpenoid containing the typical bicyclo[12.1.0]pentadecane casbane skeleton and a C5-keto group. Key strategies for the synthesis of depressin, as well as its casbene skeleton reported so far focused on the formation of the challenging 14-membered ring system. Cryptomeridiol (**2**) and 4-*epi*-cryptomeridiol (**3**) are two eudesmane-type sesquiterpene diols produced by a variety of different plants with broad biological activity. Most of the syntheses focused on the transformation of chiral pool substrates into their *trans*-6/6-fused ring system. We herein report the synthesis of compounds **1**–**3** by taking advantage of an expanded chiral pool strategy, in which the terpenoid skeletons including casbene (**4**) and germacrene A (**5**) were produced by an *Escherichia coli*-based heterologous host harboring the isopentenol utilization pathway and corresponding terpene cyclases. Two allylic oxidations of both C13 and C5 positions of the casbene skeleton followed by deoxygenation of C13 hydroxy group allowed the synthesis of compound **1** from **4** in nine steps. Selective acid-mediated 5,10-transannular cyclization of **5** followed by hydration reaction furnished both products **2** and **3** in two steps.

## Introduction

Terpenoids are one of the most important family of natural products that have been found to be produced in all domains of life. Although containing only carbon, hydrogen and oxygen atoms in most cases, over 100,000 terpenoid structures containing diverse skeletons and rich oxidative modifications have been reported [1–3]. Furthermore, the broad biological functions of terpenoids have made them to be widely applied as pharmaceuticals, flavors, and agrochemicals and so forth. In this context, terpenoid natural products have attracted lasting attention in the field of synthetic chemistry. Their complex structures and diverse skeletons have continuously inspired chemists to design novel strategies and develop new synthetic methodologies.

**Figure 1:**
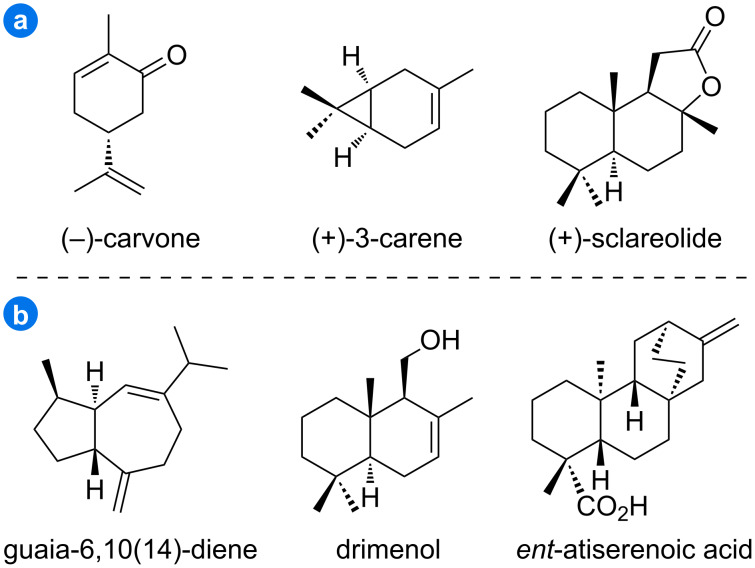
Representative terpenoid ‘chiral pool’ obtained from natural resources (a) and from heterologous production (b).

**Scheme 1:**
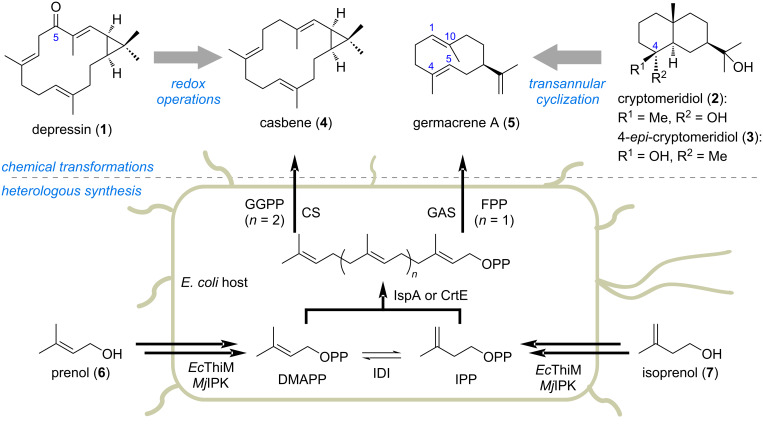
Synthetic design of depressin (**1**), cryptomeridiol (**2**), and 4-*epi*-cryptomeridiol (**3**).

For the synthesis of complex terpenoid natural products, in addition to those starting from simple and easily available starting materials, Nature has also provided us with several abundant chiral building blocks which are normally referred to as the ‘chiral pool’ to start with [4–5]. Representative terpenoid products, such as (−)-carvone, (+)-3-carene, and (+)-sclareolide that are typically isolated from renewable resources (Figure 1a), continue to be widely used as popular starting materials. It is interesting to note that for the biosynthesis of the complex terpenoid skeletons, Nature has evolved a straightforward strategy by the terpene cyclase-catalyzed cyclization of linear oligoprenyl pyrophosphates, which are generally derived from the condensation of dimethylallyl pyrophosphate (DMAPP) with different numbers of isopentenyl pyrophosphate (IPP) [6]. Two naturally existing pathways, i.e., the methylerythritol phosphate (MEP) pathway and mevalonic acid (MVA) pathway, as well as artificially designed pathways, such as the isopentenol utilization pathway, have been adopted to provide the two key five-carbon building blocks DMAPP and IPP. With the recent advancement in the field of synthetic biology, more chiral terpenes (e.g., guaia-6,10(14)-diene [7–8], drimenol [9–10], and *ent*-atiserenoic acid [11] as shown in Figure 1b) became easily available by taking advantage of Nature’s logic for terpene synthesis, either via in vitro enzymatic catalysis [12], or via heterologous production. The toolbox of the terpenoid ‘chiral pool’ has been further expanded by integrating enzymatic catalysis, hence providing us with more flexibility to design the synthetic plans of related terpenoid natural products. We herein report the synthesis of diterpene depressin (**1**), and sesquiterpenes cryptomeridiol (**2**) and 4-*epi*-cryptomeridiol (**3**) from casbene (**4**) and germacrene A (**5**), respectively, by taking advantage of this expanded ‘chiral pool’ strategy (Scheme 1).

Depressin (**1**) was isolated from the soft coral *Simularia depressa* collected from Hainan Province of China [13]. Its structure contains the typical bicyclo[12.1.0]pentadecane casbane diterpenoid skeleton with a *cis*-disubstituted cyclopropane unit, and a keto group at C5, which is the most frequently oxidized position of casbane diterpenes [14–15]. One total synthesis was reported that features a ring-closing alkyne metathesis (RCAM) reaction to forge the 14-membered macrocycle ring [16]. Besides depressin, only casbene (**4**) of the casbane family has been selected as a target for synthesis so far. The choice of efficient macrocyclization methods, including tetracarbonylnickel-promoted cyclization of allylic dibromide [17–18], copper-mediated intramolecular carbene–olefin cyclization [19], titanium-induced intramolecular carbonyl coupling [20], and organozinc carbenoid-mediated intramolecular cyclopropanation [21], for the formation of the challenging 14-membered ring remained as the key strategy for its synthesis. On the other hand, casbene synthases of different origins have been characterized that are able to convert the linear precursor geranylgeranyl pyrophosphate (GGPP) to the macrocycle skeleton [22–26], hence providing an efficient biocatalytic tool for the biosynthesis of casbene and related diterpenes [24,27].

Cryptomeridiol (**2**) and its C4-epimer 4-*epi*-cryptomeridiol (**3**) are two eudesmane-type sesquiterpene diols that have been found in different plants with biological activity including cytotoxicity and melanogenesis inhibition [28–32]. Furthermore, compound **2** is the active principle of a renal antispasmodic product Proximol^®^, derived from desert weed *Cymbopogon proximus* which is used as the folk medicine in Egypt [29]. One formal total synthesis of both compounds **2** and **3** was reported in 1988 starting from 3-vinyl-2-cyclohexen-1-one featuring a key intramolecular aldol condensation reaction [33]. On the other hand, semisyntheses of **2** were achieved starting from related terpene products, including elemol (featuring a Hg(OAc)_2_-mediated oxymetallation) [34], β-eudesmol (featuring a reductive epoxide ring-opening) [35], and ilicic acid (featuring a Curtius rearrangement reaction) [36]. It is interesting to note that compound **2** was obtained as the sole product when (+)-hedycaryol of the germacrene-type skeleton was incubated with a suspension of the root of *Cichorium intybus*, while multiple products were produced when the same compound was treated with acid or Hg(OAc)_2_, indicating (+)-hedycaryol might serve as the biosynthetic intermediate of compound **2** [37]. Terpene cyclase *Tw*CS from *Tripterygium wilfordii* responsible for the biosynthesis of **2** was discovered in 2018, hence allowing the heterologous synthesis of **2** (19.73 mg·L^−1^) by an engineered *Saccharomyces cerevisiae* strain [38]. In the same year, an eudesmanediol synthase *Zm*EDS from *Zea mays* (maize) was found to produce **3** as a minor product [39].

Our design for the chemoenzymatic synthesis of compounds **1**, **2**, and **3** is depicted in Scheme 1 [40–42]. We envisioned that by selective oxidation at C5, compound **1** could be directly obtained from casbene (**4**). The macrocyclic casbane-type skeleton could be produced by an engineered heterologous host harboring both casbene synthase (CS) and an isopentenol utilization (IU) pathway that using exogenously supplemented prenol (**6**) and isoprenol (**7**) for the terpene biosynthesis [10,43–51]. Although terpene cyclases *Tw*CS and *Zm*EDS could be considered as the suitable starting points for the direct enzymatic synthesis of **2** and **3**, respectively, in a more efficient and selective way by protein engineering, we thought that germacrene A (**5**), whose terpene cyclases are widely found in different organisms [52], could serve as a precursor for synthesis of both **2** and **3** via a transannular cyclization process [37,53–54]. A similar heterologous host harboring the same IU pathway in combination with the corresponding germacrene A synthase (GAS) could be applied to produce the desired germacrene-type sesquiterpene **5** from the two isopentenols **6** and **7**.

## Results and Discussion

To construct the heterologous hosts for the synthesis of both casbane-type diterpene and germacrane-type sesquiterpene, we firstly prepared two plasmids containing genes encoding enzymes responsible for the biosynthesis of geranylgeranyl pyrophosphate (GGPP) and farnesyl pyrophosphate (FPP) by taking advantage of the IU pathway (Table S1, Supporting Information File 1) [49]. Thus, four genes including *EcthiM* (coding for hydroxyethylthiazole kinase from *Escherichia coli*), *Mjipk* (coding for isopentenyl phosphate kinase from *Mathanocaldococcus jannaschii*), *idi* (coding for isopentenyl diphosphate isomerase from *E. coli*), and *ispA* (coding for farnesyl pyrophosphate synthase from *E. coli*) or *crtE* (coding for geranylgeranyl pyrophosphate synthase from *Pantoea agglomerans*) were cloned into the multiple cloning site-2 to give pXT02007 and pXT02013, respectively. Also, casbene synthase (CS from *Ricinus communis*) [22–23] and germacrene A synthase (GAS from *Tanacetum parthenium*) [52] encoding genes were synthesized and cloned into the pET28a-based plasmid, to give pET28a-*cs* and pET28a-*gas*, respectively. The *E. coli* heterologous host was then co-transformed with pXT02007/pET28a-*gas* and pXT02013/pET28a-*cs*, to afford strains XT02019 (for production of germacrene A) and XT02020 (for production of casbene), respectively. We then briefly investigated the fermentation conditions, including media and ratio of prenol/isoprenol supplemented, for the heterologous synthesis of **4** and **5** (Figure S1, Supporting Information File 1). To our delight, under the optimized conditions in shaking flasks, compounds **4** (110 mg·L^−1^) and **5** (100 mg·L^−1^) were successfully obtained by feeding isopentenol(s) to strains XT02020 and XT02019, respectively.

**Scheme 2:**
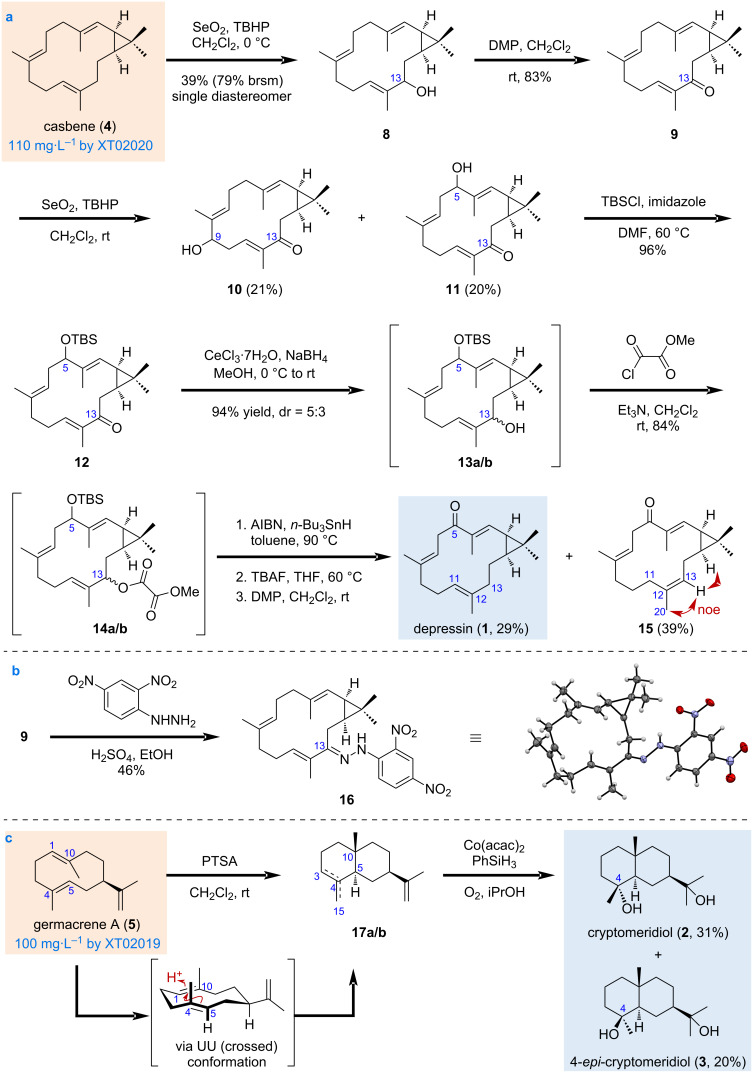
Synthesis of depressin (**1**), cryptomeridiol (**2**), and 4-*epi*-cryptomeridiol (**3**). a) Synthetic route of **1** starting from casbene (**4**). b) Preparation of 2,4-nitrophenylhydrazone derivative of 13-ketocasbene (**9**) for X-ray diffraction. c) Synthetic route of **2** and **3** starting from germacrene A (**5**).

With sufficient amounts of both compounds **4** and **5** produced by the engineered *E. coli* host by taking advantage of the expanded ‘chiral pool’ strategy, the stage was set forth for the late-stage modification of the corresponding terpene skeletons for the synthesis of the targeted natural products (Scheme 2) [55].

Formally, a selective oxidation of the allylic C5 position would give **1** directly (Scheme 2a). Considering the limited reports on direct modifications of the casbene skeleton, we thus commenced our synthesis with probing the reactivity of **4** by screening a series of conditions for allylic oxidation (Table S2 in Supporting Information File 1). However, most of the conditions tested did not give satisfactory results, as either no reaction occurred or complex mixtures were obtained when **4** was treated in metal- and non-metal-mediated reaction conditions. We have finally found that selective allylic oxidation of **4** at C13 could be achieved by the action of a catalytic amount of SeO_2_ (0.1 equiv) and *tert*-butyl hydroperoxide (TBHP, 0.6 equiv), which was added at 0 °C in three portions, affording 13-hydroxycasbene (**8**) as a single diastereomer in 48% isolated yield at milligram scale. The reaction could be performed on a gram scale albeit with a diminished isolated yield (39%, 79% brsm). We thought that with the most reactive allylic position C13 being oxidized, a second allylic oxidation would possibly occur at the desired C5 position. However, when compound **8**, as well as its acetyl ester (Ac) and *tert*-butyldimethylsilyl ether (TBS) derivatives, were subjected to several metal-mediated allylic oxidation conditions, either no reaction occurred (CuBr, RuCl_3_) or complex mixtures were obtained (CrO_3_, PDC, SeO_2_). We then transformed the allylic alcohol **8** into the less reactive enone derivative **9** using Dess–Martin periodinane (DMP) in 83% yield. The enone **9** could be condensed with 2,4-dinitrophenylhydrazine to give the hydrazone derivative **16** (CCDC 2470579) as a brownish-yellow needle suitable for X-ray diffraction (Scheme 2b) [56–57], which confirmed the absolute configuration of the casbene skeleton and the position of the first allylic oxidation. It is also interesting to note that the three double bonds in this molecule are relatively far apart from each other, and we have not isolated any products resulting from transannular cyclization of the double bonds during the conditions screening for allylic oxidation.

With enone **9** in hands, both C9 and C5 position could be hydroxylated by using the same SeO_2_-mediated allylic oxidation conditions, affording 9-hydroxy-13-ketocasbene (**10**, 21%) and 5-hydroxy-13-ketocasbene (**11**, 20%), respectively, as single diastereomers. Although the orientation of the newly introduced hydroxy group could not be assigned solely based on NMR analysis, the 5-hydroxy group of **11** will be transformed into a ketone in a later step for the synthesis of **1**. The 5-hydroxy group in **11** was then protected as a TBS ether to give **12** in 96% yield. However, direct deoxygenation of the 13-ketone of **12** was not fruitful in our hands [58–60]. We then converted enone **12** to the allylic alcohols **13a/b** as two diastereomers (dr = 5:3) under Luche reduction conditions. These two diastereomers were difficult to be separated from each other using normal column chromatography and therefore were used in the next deoxygenation steps as a mixture. Attempts to form the xanthate for Barton–McCombie deoxygenation [61–62], or the benzoate ester for reductive deoxygenation were not successful [63], and **13a/b** decomposed in the esterification step. We have finally found that the 13-hydroxy group could be smoothly removed by the methyl oxalyl ester deoxygenation method [64–65]. Thus, **13a/b** were esterified with methyl chlorooxoacetate to give **14a/b** in 84% yield, which were subjected to the radical deoxygenation conditions (AIBN, *n*-Bu_3_SnH) for 13-hydroxy removal, affording two deoxygenated products that were difficult to be separated from each other. Without further purification, the obtained mixture was treated with tetra-*n*-butylammonium fluoride (TBAF) for TBS ether removal, followed by DMP for hydroxy group oxidation, delivering the desired product **1** in 29% overall yield in three steps from **14a/b**, together with a double bond-isomerized product **15** in 39% overall yield after HPLC purification. The configuration of C12–C13 double bond of **15** was confirmed to be *Z* based on the NOESY correlations between 13-H/20-H_3_. The formation of product **15** could be attributed to the isomerization of the C13 allylic radical, generated by methyl oxalyl ester deoxygenation step, to the C11 allylic radical, followed by hydrogen abstraction from *n*-Bu_3_SnH. The physicochemical data of our synthetic sample of compound **1** matched those reported from natural resources and from total synthesis (Table S3, Supporting Information File 1) [13,16]. Thus, the synthesis of depressin (**1**) was achieved in nine steps from the expanded chiral pool casbene (**4**) prepared by the engineered *E. coli* strain XT02020.

For the synthesis of the *trans*-decalin ring system of compounds **2** and **3** (Scheme 2c), we then turned our attention to the transannular cyclization of the 10-membered cyclodecadiene ring of germacrene A (**5**). It should be noted that both 6/6- and 5/7-fused ring skeletons with different stereochemical outcomes could be obtained due to the flexible conformation of this cyclodecadiene ring system [34,37,53–54]. To our delight, treatment of compound **5** with *p*-toluene sulfonic acid (PTSA) delivered the desired *trans*-6/6-fused ring system, via its favored UU (crossed) conformation during the transannular 5,10-cyclization process [66–67], albeit with the formation of two double bond isomers **17a**/**b** that were difficult to be separated from each other. The mixture of the C3–C4 and C4–C15 double bond isomers **17a**/**b** was then subjected to the Mukaiyama hydration conditions directly without further purification, affording cryptomeridiol (**2**) in 31% yield and 4-*epi*-cryptomeridiol (**3**) in 20% yield, respectively, in two steps from the expanded chiral pool germacrene A (**5**) produced by the engineered strain XT02019 harboring the IU pathway. The two diastereomers **2** and **3** could be easily separated by column chromatography, and their physicochemical data were in consistent with those reported [35].

## Conclusion

The use of the chiral pool substrates containing complex chiral skeletons and suitable positioned functional groups would change the starting point of natural product synthesis, hence inspiring the design of new synthetic strategies. While the use of traditional chiral pool substrates derived from natural resources for synthesis has put into practice for a long time, using the expanded chiral pool substrates with additional skeletons produced enzymatically is emerging with the recent advancement in the field of synthetic biology. We have constructed the *E. coli-*based heterologous hosts harboring the IU pathway in combination with terpene cyclases that produce a diversity of terpene skeletons including casbene (**4**) and germacrene A (**5**), by supplementing the five-carbon alcohols prenol and isoprenol. Late-stage redox modification of the casbene skeleton allowed us to synthesize depressin in nine steps, while transannular cyclization of the germacrene A skeleton followed by hydration has made cryptomeridiol and 4-*epi*-cryptomeridiol easily accessible in two steps. With the ability to facilely obtain the terpene skeletons such as casbene and germacrene A by this heterologous terpene-producing platform, accessing other casbane- and germacrene-type of natural products and their analogues becomes possible if suitable chemical and enzymatic methods are available for the selective late-stage functionalization. In addition, since the precursors for terpene cyclases are general no matter they are provided by the IU pathway or MVA/MEP pathways, a diversity of terpene skeletons would be easily accessible by simply integrating known or newly discovered terpene cyclases into a heterologous host harboring the precursor-supplying pathway, hence further expanding the terpenoid chiral pool.

## Supporting Information

File 1 Materials, synthetic methods and copies of NMR spectra for all compounds.

File 2 X-ray crystal structure of **16**.

## Data Availability

All data that supports the findings of this study is available in the published article and/or the supporting information of this article.
