# Supplementary material for: Synthesis of depressin, cryptomeridiol and 4-epi-cryptomeridiol enabled by a terpenoid chiral pool-producing platform
Source: Beilstein J Org Chem. 2026 May 5;22:683–90. doi: 10.3762/bjoc.22.53 (PMC13159238; doi:10.3762/bjoc.22.53)
Supplement: File 1 — Materials, synthetic methods and copies of NMR spectra for all compounds. [file Beilstein_J_Org_Chem-22-683-s001.pdf]

BEILSTEIN JOURNAL OF ORGANIC CHEMISTRY

Supporting Information

for

Synthesis of depressin, cryptomeridiol and 4-*epi*-cryptomeridiol enabled by a terpenoid chiral pool-producing platform

Yao Kong, Tao Wang, Chen Wang, Pengcheng Zhang, Yuanning Liu, Kaibiao Wang, Fen Liu, Hongli Jia and Zhengren Xu

Beilstein J. Org. Chem. **2026,** *22,* 683–690. doi:10.3762/bjoc.22.53

Materials, synthetic methods and copies of NMR spectra for all compounds

License and Terms: This is a supporting information file under the terms of the Creative Commons Attribution License (https://creativecommons.org/licenses/by/4.0). Please note that the reuse, redistribution and reproduction in particular requires that the author(s) and source are credited and that individual graphics may be subject to special legal provisions.
The license is subject to the *Beilstein Journal of Organic Chemistry* terms and conditions: (https://www.beilstein-journals.org/bjoc/terms)

Table of contents

Material and methods	S2
Synthetic procedures and characterization data	S4
Protein and DNA sequences of related enzymes used in this study	S10
Table S1: Plasmids and strains used in this study	S12
Table S2: Screening the conditions for the allylic oxidation of casbene (**4**)	S13
Table S3: ^{1}H NMR data of depressin (**1**) in comparsion with those reported	S14
Table S4: ^{13}C NMR data of depressin (**1**) in comparsion with those reported	S15
Figure S1: Production of casbene (**4**) and germacrene A (**5**) using isopentenol utilization pathway	S16
Figures S2–S39: NMR spectra of compounds in this study	S17
References	S55

Materials and methods

Bacterial strains, plasmids, chemicals

All the strains and plasmids uesd in this study are listed in Table S1. High-fidelity Q5 DNA polymerase (NEB) and KOD One^{TM} PCR Master Mix (TOYOBO) were used for PCR amplification to construct plasmids, and 2 × Taq Plus Master Mix (Vazyme) was used for colony PCR. PCR primers were synthesized by Tsingke (Beijing). Gibson Assembly Kit (TransGen) was purchased from corresponding commercial suppliers and reactions were performed according to the manufacturer’s protocols. DNA gel extraction and plasmid preparation kits were purchased from TransGen. DNA sequencing was conducted by Majorbio. *Escherichia coli Trans*1-T1 was used for routine cloning. *E. coli* T7 was used for gene expression and microbial production. Ampicillin (Amp, 100 μg·mL^{-1}), streptomycin (Str, 50 μg·mL^{-1}), kanamycin (Kan, 50 μg·mL^{-1}) and chloramphenicol (Chl, 25 μg·mL^{-1}) were used for selection in *E. coli*. Genes were ordered from GenScript, and cloned into the *Nde*I and *Hind*III sites of pET28a(+), with codon-optimized for *E. coli* expression. Deuterated solvents such as $CDCl_3$, C_6D_6, etc. were purchased from Energy Chemical. Media components were purchased from Genstar (tryptone, yeast extract) and Solarbio (glycerol). Other common chemicals, biochemical, and media components were purchased from standard commercial sources (Adamas-beta, Stronger etc.).

General procedures

E. coli strains for routine cloning and seed culture were grown in Luria-Bertani (LB) medium or maintained on LB agar with appropriate antibiotics at 37 °C.

IR spectra were collected with a Nicolet Nexus 470 spectrometer. All ^{1}H, ^{13}C, and 2D-NMR (HSQC, ^{1}H,^{1}H COSY, HMBC, NOESY) spectra were collected at room temperature (25 °C) with a Bruker AVANCE III 400 at 400 MHz for ^{1}H and 100 MHz for ^{13}C nuclei, or a Bruker Avance III 600 at 600 MHz for ^{1}H and 150 MHz for ^{13}C nuclei. Chemical shifts were calibrated with the solvent residue signals (δ $CDCl_3$: 7.26, 77.16; benzene-*d*$_6$: 7.16, 128.06 for ^{1}H and ^{13}C NMR spectra). HRESIMS data were acquired on a Waters XEVO G2 QTOF instrument. Melting point was recorded with a Büchi M-560 melting point apparatus. Optical rotation was recorded with a Rudolph Autopol VI digital Polarimeter. GC–MS was performed on an Agilent 7890A series GC system, equipped with 5975 mass spectrometry coupled with a HP-5MS column (30 m × 250 μm × 0.25 μm, Agilent Technologies, Santa Clara, CA, USA). The carrier gas was helium with a constant flow of 1 mL·min^{-1}. Injection was in split mode (10:1) with the injector temperature set at 250 °C. The oven initial temperature was 50 °C and hold for 4 min, increased to 280 °C at 20 °C/min, and then kept at 280 °C for 3 min. X-ray crystal was diffracted with Rigaku XtaLAB Synergy single crystal X-ray diffractometer.

All the X-ray data reported here have been deposited to Cambridge Crystallographic Data Centre with Deposition Number: CCDC 2470579 (for **16**).

Fermentation and purification of germacrene A (5) and casbene (4)

Seed culture was prepared by inoculating a single colony of the *E. coli* strain XT02019 (harboring plamids pXT02007 and pET28a-*gas* for producing germacrene A) or XT02020 (harboring plamids pXT02013 and pET28a-*cs* for producing casbene) into the LB medium containing streptomycin (50 μg·mL^{-1}) and kanamycin (50 μg·mL^{-1}) followed by growing at 37 °C with shaking at 200 rpm overnight. The seed culture (10 mL, 1% (v/v)) was innoculated to a 2 liter-flask containing 1 L of the indicated medium (TB, TB-20, M9Y5 and AM mineral media) with the appropriate antibiotics. The culture was incubated at 37 °C until an OD_{600} value of 0.8 was reached. The culture was then cooled to 4 °C, followed by addition of IPTG (0.1 mM for producing germacrene A, 0.5 mM for producing casbene), 8 mM isoprenol (≈800 μL) (for producing germacrene A), or 6 mM prenol (≈600 μL) and 2 mM isoprenol (≈200 μL) (for producing casbene). The resultant culture was incubated at 18 °C for 72 h.

A mixture of the fermentation broth (1 L) and EtOAc (500 mL) was filtrated through a pad of Celite. The aqueous phase was extracted with EtOAc (500 mL × 3). The combined organic layers were dried over Na_2SO_4. The solvent was removed under reduced pressure. The residue was purified by silica gel chromatography, and eluted with petroleum ether (PE) to give germacrene A (100 mg) or casbene (110 mg), as colorless oils.

Germacrene A (5)
R*f* = 0.96 (PE);
$[\alpha]_D^{20}$ +20.6 (*c* 0.75, CCl_4); +42.1 (*c* 1.0, CCl_4)[1];
^{13}C NMR (100 MHz, $CDCl_3$) *δ* 153.9, 138.3, 131.8, 129.1, 126.5, 107.4, 51.5, 41.9, 39.7, 35.0, 33.8, 36.9, 24.5, 20.4, 20.1, 16.8, 16.4, 15.6.

Casbene (4)[2]
R*f* = 0.95 (PE);
$[\alpha]_D^{25}$ -161 (*c* 1.0, $CDCl_3$);
IR (neat)ν_{max} 2977, 2922, 2858, 1450, 1376 cm^{-1};
^{1}H NMR (400 MHz, $CDCl_3$) *δ* 5.03 (s, 1H), 4.97 – 4.88 (m, 2H), 2.28 – 1.97(m, 8H), 1.96 – 1.84 (m, 2H), 1.69 (m, 1H), 1.61 (s, 3H), 1.55 (s, 3H), 1.49 (s, 3H), 1.12 (t, *J* = 8.3 Hz, 1H), 1.02 (s, 3H), 0.97 (m, 1H), 0.91 (s, 3H), 0.53 (t, *J* = 9.4 Hz, 1H);
^{13}C NMR (100 MHz, $CDCl_3$) *δ* 136.2, 135.3, 133.3, 126.1, 124.1, 121.8, 41.0, 40.0, 39.8, 31.1, 29.2, 26.3, 25.5, 24.6(2C), 20.0, 16.8, 16.6, 16.1, 16.0.

Media for fermentation include:
Terrific Broth (TB) medium: yeast extract (24 g·L^{-1}), tryptone (12 g·L^{-1}), glycerol (5 g·L^{-1}), and 100 mL of phosphate buffer (0.17 M KH_2PO_4, 0.72 M K_2HPO_4).
TB-20 medium: yeast extract (24 g·L^{-1}), tryptone (12 g·L^{-1}), glycerol (20 g·L^{-1}), and 100 mL of phosphate buffer (0.17 M KH_2PO_4, 0.72 M K_2HPO_4).
M9Y5 medium: glucose (20 g·L^{-1}), yeast extract (5 g·L^{-1}), $MgSO_4$ (1 mM), $CaCl_2$ (0.3 mM), biotin (1 μg), thiamin (1 μg), M9 salt solution [10 X, $Na_2HPO_4·2H_2O$ (75.2 g·L^{-1}), KH_2PO_4 (30 g·L^{-1}), NaCl (5 g·L^{-1}), NH_4Cl (5 g·L^{-1})] and 10 mL of trace elements solution [EDTA (5 g·L^{-1}), $FeCl_3·6H_2O$ (0.83 g·L^{-1}), $ZnCl_2$ (84 mg·L^{-1}), $CuCl_2·2H_2O$ (13 mg·L^{-1}), $CoCl_2·2H_2O$ (10 mg·L^{-1}), H_3BO_3 (10 mg·L^{-1}), $MnCl_2·4H_2O$ (1.6 mg·L^{-1})].
Concentrated stocks of biotin (1 mg·mL^{-1}), thiamin (1 mg·mL^{-1}), and trace elements solution (100 X) are sterilized by a 0.22-μM filter. They were added aseptically to the medium when used.
Mineral 'AM' medium: KH_2PO_4 (4.2 g·L^{-1}), $K_2HPO_4·3H_2O$ (15.7 g·L^{-1}), $(NH_4)_2SO_4$ (2.0 g·L^{-1}), citric acid (1.7 g·L^{-1}), EDTA (8.4 mg·L^{-1}), glycerol (30 g·L^{-1}), yeast extract (5 g·L^{-1}). Stock solution of 5 mL·L^{-1} of $MgSO_4·7H_2O$ (1 M), 1 mL·L^{-1} of thiamine·HCl (4.5 g·L^{-1}), and 10 mL·L^{-1} of batch trace metal solution were added aseptically to the medium when used. Finally, the pH was adjusted to 7.0 by 10 M NaOH.
Batch trace metal solution contains the following ingredient and dissolved in 1 M HCl: $CoCl_2·6H_2O$ (0.25 g·L^{-1}), $MnCl_2·4H_2O$ (1.5 g·L^{-1}), $CuCl_2·2H_2O$ (0.15 g·L^{-1}), H_3BO_3 (0.3 g·L^{-1}), $Na_2MoO_4·2H_2O$ (0.25 g·L^{-1}), $Zn(OAc)_2·2H_2O$ (1.3 g·L^{-1}), Fe(III)citrate (10 g·L^{-1}).

Synthetic procedures and characterization data

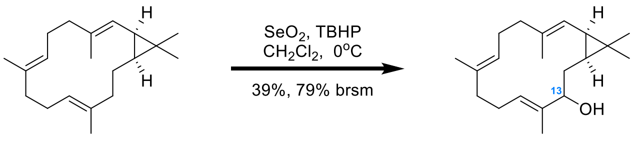

casbene (**4**) 13-hydroxy-casbene (**8**)

13-Hydroxycasbene (8): To a stirring solution of casbene (**4**, 1.10 g, 4.04 mmol, 1.0 equiv) in CH_2Cl_2 (150 mL) at 0 °C was added SeO_2 (48 mg, 0.4 mmol, 0.1 equiv). The resultant mixture was allowed to stir at 0 °C for 5 min, followed by addition of TBHP (440 µL, 5–6 mmol in decane) in three portions over 6 h. After completion of the additions, the reaction mixture was stirred at the same temperature for additional 6 h before the reaction being quenched by addition of sat. aq. $Na_2S_2O_3$ (50 mL). The mixture was extracted with CH_2Cl_2 (100 mL × 3), the combined organic layers were washed with brine (150 mL), dried over Na_2SO_4, filtered, and concentrated in vacuo. The crude residue was purified by flash column chromatography on silica gel (PE/EtOAc = 10:1) to give casbene (**4**, 547.6 mg, 50%), and 13-hydroxycasbene (**8**, 460.4 mg, 39%) as a colorless oil.

R*f* = 0.35 (PE/EtOAc = 10:1);
$[\alpha]_D^{20}$ -128 (*c* 1.0, $CHCl_3$);
IR (neat) ν_{max} 3340, 2978, 2961, 1436, 1377, 1050 cm^{-1};
^{1}H NMR (400 MHz, $CDCl_3$) *δ* 5.15 (m, 1H), 4.87-4.80 (m, 2H), 3.92(dd, *J* = 8.4, 1.4 Hz, 1H), 2.31-1.84 (m, 8H), 1.76 (dd, *J* = 13.6, 8.7 Hz,1H), 1.65 (s, 3H), 1.61 (s, 3H), 1.55 (s, 3H), 1.25-1.21(m, 2H), 1.07 (s, 3H),1.07-1.02 (m, 1H), 0.95 (s, 3H), 0.92-0.86 (m, 1H);
^{13}C NMR (100 MHz, $CDCl_3$) *δ* 137.8, 137.1, 133.1, 126.2, 126.0, 120.8, 80.2, 39.4, 39.1, 32.7, 28.9, 28.0, 25.5, 24.8, 23.4, 19.7, 16.5, 15.9, 15.6, 10.7;
HR-ESI-MS *m/z*: 287.2372 $[M - H]^-$ (calcd. for $C_{20}H_{31}O^-$ 287.2375).

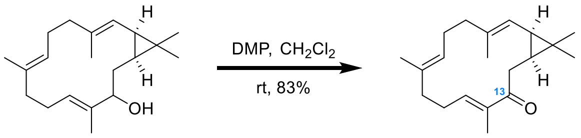

13-hydroxy-casbene (**8**) 13-keto-casbene (**9**)

13-Ketocasbene (9) was prepared in a manner similar to that of reported[24]: To a stirring solution of 13-hydroxycasbene (**8**, 110.0 mg, 0.39 mmol, 1.0 equiv) in CH_2Cl_2 (20 mL) at room temperature was added Dess–Martin periodinane (DMP, 182.9 mg, 0.43 mmol, 1.1 equiv). The reaction mixture was stirred at room temperature until TLC analysis showed the full consumption of the starting material. The reaction was quenched by addition of sat. aq. $NaHCO_3$ solution (20 mL), and the mixture was extracted with CH_2Cl_2 (20 mL × 3). The combined organic layers were dried over Na_2SO_4, and evaporated to dryness. The crude residue was purified with silica gel chromatography (CH_2Cl_2/MeOH 25:1) to yield 13-ketocasbene (**9**, 93.4 mg, 83%) as a colorless oil.

R*f* = 0.61 (PE/EtOAc = 10:1);
$[\alpha]_D^{20}$ -132 (*c* 1.0, $CHCl_3$);
IR (neat) ν_{max} 2921, 1665, 1441, 1380, 1287 cm^{-1};
^{1}H NMR (400 MHz, $CDCl_3$) *δ* 6.49 (br t, *J* = 6.2 Hz 1H), 4.96 (m, 1H), 4.87 (d, *J* = 8.2, 1H), 2.82 (dd, *J* = 14.6, 9.9 Hz, 1H), 2.36-2.05 (m, 9H), 1.74 (s, 3H), 1.56 (s, 3H), 1.55 (s, 3H), 1.26-1.22 (m, 1H), 1.14-1.10 (m, 1H), 1.09 (s, 3H), 0.95 (s, 3H);
^{13}C NMR (100 MHz, $CDCl_3$) *δ* 203.2, 143.6, 137.5 (2C), 133.1, 126.5, 120.6, 39.8, 38.2, 32.2, 28.7, 26.1, 25.9, 25.6, 25.1, 19.7, 16.1, 15.9, 15.6, 11.6;
HR-ESI-MS *m/z*: 287.2367 $[M + H]^+$ (calcd. for $C_{20}H_{31}O^+$ 287.2375).

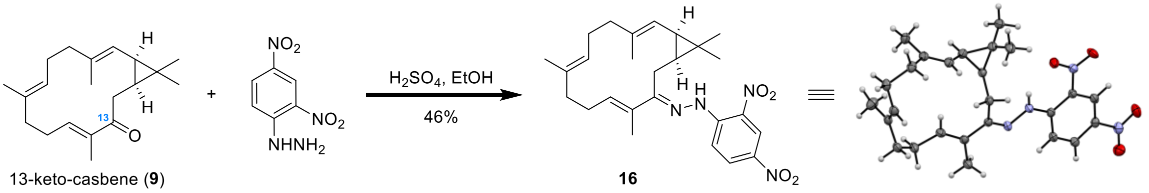

Compound 16 was prepared in a manner similar to that of reported[24]: Solid 2,4-dinitrophenylhydrazine (82.4 mg, 0.42 mmol, 4.0 equiv) and conc. H_2SO_4 (2.0 mL) were mixed and stirred at room temperature for 10 min to dissolve all the solids. To the resultant reaction mixture with stirring was added a solution of 13-ketocasbene (30.0 mg, 0.10 mmol,1.0 equiv) in 95% EtOH (3.0 mL) dropwise. The reaction mixture was allowed to stir at room temperature for 30 min until the precipitation of a brownish-yellow solid was observed. The mixture was filtered off, and the solid was washed with EtOH (≈2 mL). The solid was desiccated to afford compound **16** (22.3 mg, 0.05 mmol, 46%) as a brownish-yellow solid.

R*f* = 0.45 (PE/EtOAc = 1:1);
m.p. 157-161°C (brownish-yellow needle, PE/CH_2Cl_2, 5:1);
$[\alpha]_D^{20}$ +196 (*c* 0.1, $CHCl_3$);
IR (neat) ν_{max} 3304, 2952, 2924, 2853, 1616, 1592, 1537, 1518, 1423, 1377, 1334, 1311, 1267, 1132 cm^{-1};
^{1}H NMR (400 MHz, $CDCl_3$) δ 11.39 (s, 1H), 9.15 (d, *J* = 2.6 Hz, 1H), 8.32 (dd, *J* = 9.6, 2.5 Hz, 1H), 8.02 (d, *J* = 9.6 Hz, 1H), 6.10 (brdd, *J* = 3.1, 3.4 Hz, 1H), 5.10-5.04 (m, 1H), 4.90 (d, *J* = 10.2 Hz, 1H), 2.61 (dd, *J* = 14.9, 7.2 Hz, 1H), 2.49-2.05 (m, 9H), 2.00 (s, 3H), 1.68 (s, 3H), 1.61 (s, 3H), 1.46-1.39 (m, 1H), 1.16 (s, 3H), 1.13 (s, 3H), 0.52 (brt, *J* = 7.3 Hz, 1H);
^{13}C NMR (100 MHz, $CDCl_3$) δ 160.1, 145.2, 137.8, 137.1, 135.5, 134.0, 133.3, 130.0, 129.6, 126.5, 123.7, 120.2, 116.9, 40.5, 38.6, 28.3, 26.3, 25.6, 25.6, 25.5, 21.2, 20.5, 15.6, 15.5, 15.4, 13.5;
HR-ESI-MS *m/z*: 465.2502 $[M - H]^-$ (calcd. for $C_{26}H_{33}N_4O_4$ 465.2505).

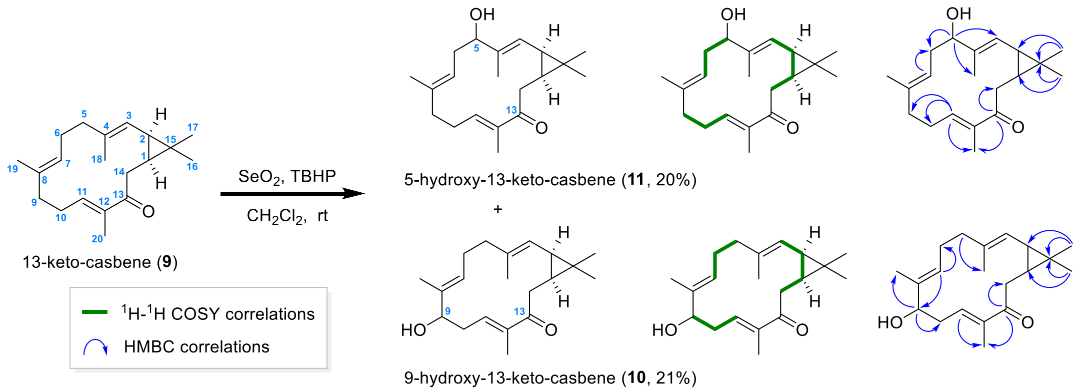

To a stirring solution of 13-ketocasbene (**9**, 54.0 mg, 0.19 mmol, 1.0 equiv) and SeO_2 (2.1 mg, 0.02 mmol, 0.1 equiv) in CH_2Cl_2 (20.0 mL) was added TBHP (34.0 μL, 1–1.2 mmol in decane, 0.04 mmol, 0.2 equiv). The reaction mixture was allowed to stir at room temperature for 2 h, during which period an additional amount of TBHP (102.0 μL, 1–1.2 mmol in decane, 0.12 mmol, 0.6 equiv) was added in three portions. The reaction was quenched by addition of sat. aq. $Na_2S_2O_3$ (20 mL). The mixture was extracted with CH_2Cl_2 (20 mL × 3), and the combined organic layers were washed with brine (80 mL), dried over Na_2SO_4, filtered, and concentrated in vacuo. The crude residue was purified by flash column chromatography on silica gel (PE/EtOAc 5:1) to give 5-hydroxy-13-ketocasbene (**11**, 11.3 mg, 20.1%) and 9-hydroxy-13-ketocasbene (**10**, 11.6 mg, 21.2%), respectively.

5-Hydroxy-13-ketocasbene (11)
R$_f$ = 0.23 (PE/EtOAc = 4 : 1);
$[\alpha]_D^{20}$ -118 (*c* 0.1, $CHCl_3$);
IR (neat) v_{max} 3419, 2923, 2867, 1659, 1447, 1377, 1288, 1250, 1015 cm^{-1};
^{1}H NMR (400 MHz, $CDCl_3$) δ 6.43 (brt, *J* = 5.5 Hz, 1H), 5.13 (d, *J* = 8.7 Hz, 1H), 4.88(brt, *J* = 8.5 Hz, 1H), 4.10 (dd, *J* = 10.5, 5.2 Hz, 1H), 2.77 (dd, *J* = 14.4, 9.7 Hz, 1H), 2.39-2.09 (m, 7H), 1.76 (s, 3H), 1.62 (s, 3H), 1.59 (s, 3H), 1.29 (t, *J* = 8.8 Hz,1H), 1.17 (td, *J* = 9.8, 1.3 Hz, 1H), 1.11 (s, 3H), 0.99 (s, 3H);
^{13}C NMR (100 MHz, $CDCl_3$) δ 203.0,143.4, 139.0, 137.5, 134.8, 124.7, 121.6, 79.3, 38.0, 33.2, 32.1, 28.6, 26.8, 25.8, 25.2, 20.3, 16.1, 15.8, 11.7, 10.5;
HR-ESI-MS *m/z* 325.2144 $[M + Na]^+$ (calcd. for $C_{20}H_{30}O_2Na^+$ 325.2143).

9-Hydroxy-13-ketocasbene (10)
R$_f$ = 0.20 (PE/EtOAc = 4 : 1);
$[\alpha]_D^{20}$ -126 (*c* 0.1, $CHCl_3$);
IR (neat) v_{max} 3438, 2923, 1661, 1449, 1377, 1290, 1251, 1040 cm^{-1};
^{1}H NMR (400 MHz, $CDCl_3$) δ 6.25 (td, *J* = 6.7, 1.2 Hz, 1H), 5.22 (dd, *J* = 9.8, 3.1 Hz, 1H), 4.84(d, *J* = 7.9 Hz, 1H), 4.17 (dd, *J* = 8.8, 6.8 Hz, 1H), 2.76 (dd, *J* = 14.6, 9.7 Hz, 1H), 2.54 (brt, *J* = 6.8 Hz, 2H), 2.42-2.28 (m, 2H), 2.17 (dd, *J* = 14.6, 1.5 Hz, 1H), 2.14-2.05 (m, 2H), 1.78 (s, 3H), 1.56 (s, 3H), 1.55 (s, 3H), 1.23 (brt, *J* = 8.2 Hz, 1H), 1.12-1.08 (m, 1H), 1.10 (s, 3H), 0.93 (s, 3H);
^{13}C NMR (100 MHz, $CDCl_3$) δ 202.9, 138.8, 138.7, 137.5, 135.9, 129.2, 120.7, 77.9, 39.4, 33.8, 32.4, 28.6, 25.9, 25.6, 25.1, 19.8, 16.2, 15.9, 11.9, 10.2;
HR-ESI-MS *m/z* 325.2147 $[M + Na]^+$ (calcd. for $C_{20}H_{30}O_2Na^+$ 325.2143).

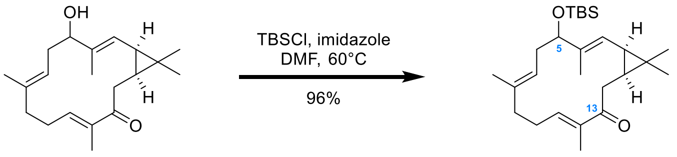

11 **12**

To a solution of 5-hydroxy-13-ketocasbene (**11**, 12.0 mg, 0.04 mmol, 1.0 equiv) in DMF (5 mL) was added TBSCl (6.4 mg, 0.04 mmol, 1.1 equiv) and imidazole (3.0 mg, 0.04 mmol, 1.1 equiv). The reaction mixture was stirred at 60 °C until TLC analysis showed the full consumption of the starting material. The reaction was quenched by addition of sat. aq. NH_4Cl solution (10 mL), and the mixture was extracted with CH_2Cl_2 (15 mL × 3). The combined organic layers were dried over Na_2SO_4 and evaporated to dryness. The crude product was purified with silica gel chromatography (PE/EtOAc 20:1) to yield compound **12** (15.7 mg, 96%) as a colorless oil.

R$_f$ = 0.74 (PE/EtOAc = 10 : 1);
$[\alpha]_D^{20}$ -88 (*c* 1.0, $CHCl_3$);
IR (neat) v_{max} 2953, 2928, 2856, 1664, 1462, 1251, 1066, 836 cm^{-1};
^{1}H NMR (400 MHz, $CDCl_3$) δ 6.46 (t, *J* = 6.7 Hz, 1H), 4.96 (d, *J* = 9.4 Hz, 1H), 4.90(brt, *J* = 7.32 Hz, 1H), 3.99 (dd, *J* = 11.0, 4.4 Hz, 1H), 2.81 (dd, *J* = 13.7, 9.7 Hz, 1H), 2.39-2.04 (m, 7H), 1.76 (s, 3H), 1.60 (s, 3H), 1.59 (s, 3H), 1.29-1.17 (m,2H), 1.11 (s, 3H), 0.94 (s, 3H), 0.88 (s, 9H), 0.05-0.00 (m, 6H);
^{13}C NMR (100 MHz, $CDCl_3$) δ 203.3, 143.5, 138.8, 137.3, 134.4, 122.5, 122.4, 79.9, 38.0, 35.0, 32.5, 28.8, 27.4, 26.0 (3C), 25.9, 25.2, 21.0, 18.4, 16.1, 15.8, 11.7, 10.7, -4.5, -4.8;
HR-ESI-MS *m/z* 439.3001 $[M + Na]^+$ (calcd. for $C_{26}H_{44}O_2NaSi^+$ 439.3002).

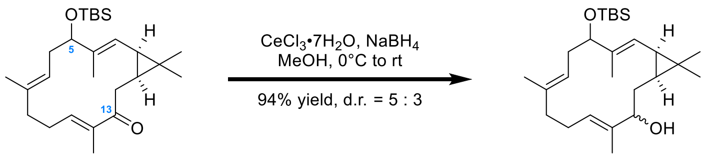

12 **13a/b**

To a stirring solution of compound **12** (15.0 mg, 0.04 mmol, 1.0 equiv) in MeOH (5 mL) was added $CeCl_3 \cdot 7H_2O$ (17.4 mg, 0.05 mmol, 1.3 equiv) in one portion. The suspension was allowed to stir at 0 °C for 10 min before $NaBH_4$ (1.8 mg, 0.05 mmol, 1.3 equiv) was added. The reaction mixture was allowed to warm to room temperature and stirred for another 2 h at the same temperature. When TLC showed the full consumption of the starting material, aq. HCl (5 mL, 1.0 N) was added, and the mixture was extracted with ether (5 mL × 3). The combined organic layers were washed successively with water and brine, dried over Na_2SO_4 and concentrated under vacuum. The resultant residue was purified by flash column chromatography (PE/EtOAc 10:1) to afford compounds **13a/b** as a mixture of two diastereomers (14.0 mg, 94% overall yield, dr = 5:3), which were difficult to separate from each other and used directly in the next step.

R_f = 0.42 (PE/EtOAc = 10 : 1);
^{1}H NMR (400 MHz, $CDCl_3$), both diastereomers are shown: δ 5.21-5.14 (m, 1.6H), 4.91 (d, J = 9.4 Hz, 0.6H), 4.84 (d, J = 9.4 Hz, 1H), 4.78-4.70 (m, 1.6H), 4.26 (brt, J = 3.7 Hz, 1H), 3.98 (dd, J = 10.9, 4.2 Hz, 1H), 3.93 (brt, J = 6.6 Hz, 0.6H), 3.88 (dd, J = 10.7, 4.3 Hz, 1H), 2.34-1.76 (m, 12.4H), 1.68 (s, 3H), 1.64 (s, 3H), 1.63 (s, 1.8H), 1.61 (s, 1.8H), 1.59 (s, 3H), 1.57 (s, 1.8H), 1.43-1.27 (m, 3.2H), 1.12 (s, 3H), 1.09 (s, 1.8H), 0.94 (s, 1.8H), 0.90 (s, 3H), 0.87 (s, 14.4H), 0.04-–0.01 (m, 9.6H);
^{13}C NMR (100 MHz, $CDCl_3$), both diastereomers are shown: δ 138.3, 137.5, 137.4, 137.0, 134.8, 134.3, 127.2, 125.9, 123.7, 123.6, 121.4, 119.8, 80.1, 79.8, 79.6, 76.8, 39.0, 38.3, 35.0, 34.8, 32.1, 31.6, 29.8, 29.2, 28.9, 28.4, 26.1(2C), 26.1(2C), 26.0, 25.3, 24.6, 24.2, 23.7, 21.3, 20.4, 18.4, 18.4, 17.1, 16.2, 16.0, 15.8, 12.7, 12.3, 10.8, 10.4, -4.5, -4.8.

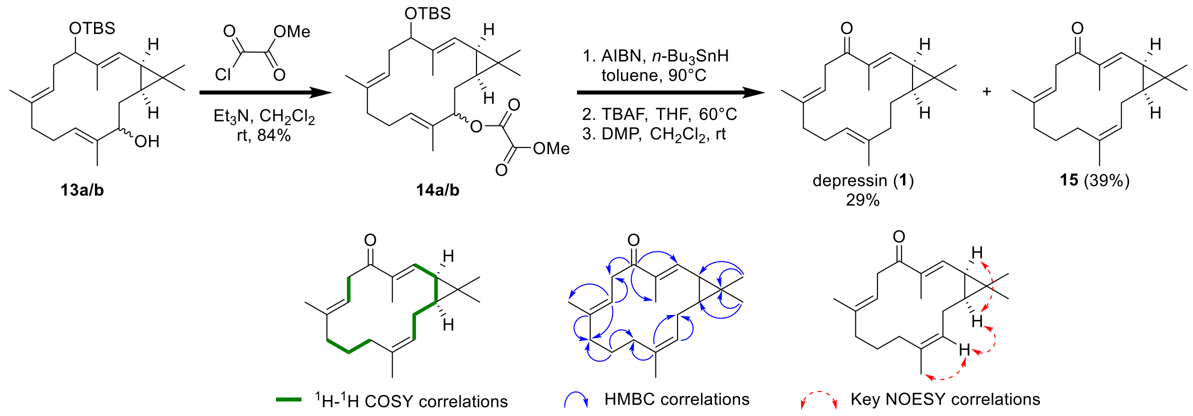

To a stirred solution of compounds **13a/b** (38.5 mg, 0.09 mmol, 1.0 equiv) in CH_2Cl_2 (5.0 mL) was added methyl oxalyl chloride (21.0 µL, 0.18 mmol, 2.0 equiv) and Et_3N (30.0 µL, 0.18 mmol, 2.0 equiv). The reaction mixture was allowed to stir at room temperature for 30 min. The reaction was quenched by addition of sat. aq. NH_4Cl solution (5 mL), and the mixture was extracted with CH_2Cl_2 (10 mL × 3). The combined organic layers were dried over Na_2SO_4, and evaporated to dryness. The crude product was purified with silica gel chromatography (PE/EtOAc 10:1) to yield compounds **14a/b** (38 mg, 84% overall yield, dr = 5:3) as a colorless oil, which was used in the next step as a mixture of two diastereomers.

To a solution of the compounds **14a/b** (15.0 mg, 0.03 mmol, 1.0 equiv) in anhydrous toluene (3.0 mL) was added tributyltin hydride (n-Bu_3SnH, 54.2 µL, 0.15 mmol, 5.0 equiv) and 2,2'-azobisisobutyronitrile (AIBN, 1.0 mg, 0.006 mmol, 0.2 equiv). The reaction mixture was heated

to reflux for 1 h under nitrogen atmosphere. The mixture was cooled, concentrated in vacuo to give the crude deoxygenated products (4.0 mg), which were used for the next step without further purification.

To a solution of the crude deoxygenated products (4.0 mg, 0.01 mmol) in THF (2 mL) was added TBAF (100 μL, 0.1 mmol). The reaction mixture was heated to reflux for 3 h until TLC analysis showed the full consumption of the starting material. The reaction mixture was quenched by adding sat. aq. NH_4Cl solution at room temperature and extracted with ether (5 mL × 3). The combined organic layers were washed with water and brine, dried over Na_2SO_4 and concentrated under reduced pressure. The resultant residue was briefly filtered through a pad of silica gel and washed with PE/EtOAc 10:1 to afford the crude TBS-deprotected products (2.8 mg), which were used for the next step without further purification.

The DMP-mediated oxidation was performed in a manner similar to that of reported[24]: To a stirring solution of the obtained crude TBS-deprotected products (2.8 mg) in CH_2Cl_2 (2 mL) at room temperature was added Dess–Martin periodinane (DMP, 5.1 mg, 0.012 mmol). The reaction mixture was stirred at room temperature until TLC analysis showed the full consumption of the starting material. The reaction was quenched by addition of sat. aq. $NaHCO_3$ solution (2 mL), and the mixture was extracted with CH_2Cl_2 (5 mL × 3). The combined organic layers were dried over Na_2SO_4, and evaporated to dryness. The resultant residue was purified with silica gel chromatography (PE/EtOAc 20:1) to yield the alkene products, which were further purified by preparative HPLC (eluting with CH_3CN/H_2O 87:13, 8.0 mL/min) to yield depressin (**1**, 0.8 mg, 29%) and its double bond regioisomer (**15**, 1.1 mg, 39%), respectively.

Depressin (1)
R*f* = 0.65 (PE/EtOAc = 10 : 1);
$[\alpha]_D^{20}$ -80 (*c* 0.05, $CHCl_3$); **$[\alpha]_D^{20}$** -85 (*c* 0.02, $CHCl_3$)[3];
IR (neat) ν_{max} 3727, 2923, 2850, 2206, 1655, 1460 cm^{-1};
^{1}H- and **^{13}C-NMR** data are listed in Tables S3-S4;
HR-ESI-MS *m/z* 287.2369 $[M + H]^+$ (calcd. for $C_{20}H_{31}O^+$ 287.2369).

Compound 15
R*f* = 0.65 (PE/EtOAc = 10:1);
$[\alpha]_D^{20}$ +60 (*c* 0.05, $CHCl_3$);
IR (neat) ν_{max} 3723, 2921, 2850, 1699, 1656, 831 cm^{-1};
^{1}H NMR (600 MHz, $CDCl_3$) δ 6.54 (d, *J* = 10.9 Hz, 1H, H3),5.16 (qt, *J* = 4.9, 1.4 Hz, 1H, H13), 5.13 (m, 1H, H7) 3.83 (dd, *J* = 14.1, 10.2 Hz, 1H, H6a), 2.92 (d, *J* = 14.2 Hz, 1H, H6b),2.15 (td, *J* = 13.0, 3.4 Hz, 1H, H11a), 2.12 (dd, *J* = 12.9, 3.3 Hz, 1H, H9a), 2.07 (dd, *J* = 14.6, 5.1 Hz, 1H, H14a), 1.94 (m, 1H, H14b), 1.89 (td, *J* = 12.9, 3.2 Hz, 1H, H9b), 1.85 (d, *J* = 1.2 Hz, 3H, H18), 1.70 (s, 3H, H19), 1.69 (t, *J* = 1.4 Hz, 3H, H20), 1.67 (m, 1H, H10a), 1.51 (dd, *J* = 10.9, 8.4 Hz, 1H, H2), 1.43 (ddd, *J* = 13.1, 6.4, 6.3 Hz, 1H, H11b), 1.29 (m, 1H, H10b),1.16 (s, 3H, H17), 1.08 (s, 3H, H16), 1.06 (br t, *J* = 1.4 Hz, 1H, H1);
^{13}C NMR (150 MHz, $CDCl_3$) δ 200.7, 143.9, 137.1, 135.7, 135.0, 123.3, 122.3, 40.5, 38.6, 34.3, 30.0, 29.2, 28.2, 25.5, 25.4, 23.8, 23.2, 16.2, 16.1, 11.7;
HR-ESI-MS *m/z* 287.2365 $[M + H]^+$ (calcd. for $C_{20}H_{31}O^+$ 287.2369).

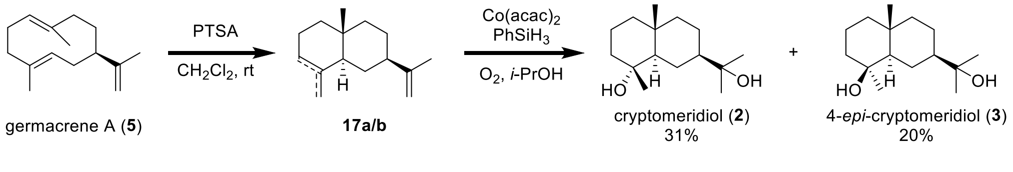

Cryptomeridiol (2) and **4-*epi*-cryptomeridiol (3)**: To a solution of germacrene A (**5**, 204 mg, 1 mmol, 1.0 equiv) in CH_2Cl_2 (10 mL) was added PTSA (8.6 mg, 0.05 mmol, 0.05 equiv), the

reaction mixture was stirred at room temperature until the TLC indicated the full consumption of the starting material. The reaction was quenched by sat. aq. $NaHCO_3$ and extracted with CH_2Cl_2 (10 mL × 2). The combined organic phases were washed with brine, dried with Na_2SO_4 and concentrated in vacuo to give a mixture of two double-bond isomers **17a/b**, which were used directly for the next step without further purification.

The crude product obtained above was dissolved in iPrOH (50 mL) and $Co(acac)_2$ (25.7 mg, 0.1 mmol, 0.1 equiv) was added at room temperature. The mixture was fitted with a balloon of O_2 and purged with sonication for 5 min before $PhSiH_3$ (212.1 mg, 1.96 mmol, 2.0 equiv) was added. The reaction mixture was stirred at room temperature under O_2 atmosphere for 36 h. After the TLC indicated the full consumption of the starting material, PPh_3(130 mg, 0.49 mmol, 0.5 equiv) was added, and the mixture was stirred for an additional 2 h. The solvent was removed and the residue was purified by flash column chromatography (PE/EtOAc 3:1 to 1:2) to afford 4-*epi*-cryptomeridiol (**3**, 48 mg, 20%) and cryptomeridiol (**2**, 74 mg, 31%) as colorless solids.

4-*epi*-Cryptomeridiol (3)
R_f = 0.25 (PE/EtOAc = 1 : 1);
$[\alpha]_D^{25}$ +4.0 (*c* 1.7, $CHCl_3$); $[\alpha]_D^{25}$ +26.1 (*c* 0.82, $CHCl_3$)[4]
IR (neat): 3406, 2930, 2845, 1457, 1376, 1185 cm^{-1};
^{1}H NMR (400 MHz, $CDCl_3$) δ 1.90-1.77 (m, 2H), 1.72-1.64 (m, 1H), 1.62-1.53 (m, 2H), 1.47-1.30 (m, 6H), 1.25 (s, 1H), 1.21 (d, *J* = 1.9 Hz, 6H), 1.17 (s, 3H), 1.15-1.03 (m, 4H), 1.02 (s, 3H);
^{13}C NMR (100 MHz, $CDCl_3$) δ 73.2, 72.2, 51.8, 50.1, 44.0, 41.7, 41.5, 33.8, 30.4, 27.6, 26.9, 22.6, 21.5, 18.8, 18.2;
HR-ESI-MS *m/z* 223.2062 $[M - OH]^+$ (calcd. for $C_{15}H_{27}O^+$ 223.2056).

Cryptomeridiol (2)
R_f = 0.20 (PE/EtOAc = 1 : 1);
$[\alpha]_D^{25}$ = -19.9 (*c* 3.3, $CHCl_3$); $[\alpha]_D^{25}$ -25.3 (*c* 1.11, $CHCl_3$)[4];
IR (neat): 3368, 2970, 2928, 2866, 2848, 1457, 1382, 1181, 1139, 1124, 1091, 1059 cm^{-1};
^{1}H NMR (400 MHz, $CDCl_3$) δ 1.93 (dq, *J* = 12.6, 3.4, 2.9 Hz, 1H), 1.79 (dtd, *J* = 12.4, 3.2, 1.6 Hz, 1H), 1.64-1.53 (m, 3H), 1.51 (t, *J* = 3.2 Hz, 1H), 1.48-1.32 (m, 5H), 1.31-1.25 (m, 1H), 1.23 (m, 1H), 1.20 (s, 6H), 1.19-1.15 (m, 1H), 1.12 (s, 3H), 1.09-0.97 (m, 2H), 0.86 (s, 3H);
^{13}C NMR (100 MHz, $CDCl_3$) δ 73.1, 72.4, 54.9, 50.0, 44.7, 43.5, 41.1, 34.6, 27.5, 27.1, 22.7, 22.6, 21.6, 20.3, 18.8;
HR-ESI-MS *m/z* 258.2433 $[M + NH_4]^+$ (calcd. for $C_{15}H_{32}NO_2^+$ 258.2433).

Protein and DNA sequences of related enzymes used in this study

Protein sequence of casbene synthase from *Ricinus communis* (accession number: NP_001411127.1)
MALPSAAMQSNPEKLNLFHRLSSLPTTSLEYGNNRFPFFSSSAKSHFKKPTQACLSSTTHQEV
RPLAYFPPTVWGNRFASLTFNPSEFESYDERVIVLKKKVKDILISSTSDSVETVILIDLLCRLGVS
YHFENDIEELLSKIFNSQPDLVDEKECDLYTAAIVFRVFRQHGFKMSSDVFSKFKDSDGKFKES
LRGDAKGMLSLFEASHLSVHGEDILEEAFAFTKDYLQSSAVELFPNLKRHITNALEQPFHSGVP
RLEARKFIDLYEADIECRNETLLEFAKLDYNRVQLLHQQELCQFSKWWKDLNLASDIPYARDR
MAEIFFWAVAMYFEPDYAHTRMIIAKVVLLISLIDDTIDAYATMEETHILAEAVARWDMSCLEKLP
DYMKVIYKLLLNTFSEFEKELTAEGKSYSVKYGREAFQELVRGYYLEAVWRDEGKIPSFDDYL
YNGSMTTGLPLVSTASFMGVQEITGLNEFQWLETNPKLSYASGAFIRLVNDLTSHVTEQQRGH
VASCIDCYMNQHGVSKDEAVKILQKMATDCWKEINEECMRQSQVSVGHLMRIVNLARLTDVS
YKYGDGYTDSQQLKQFVKGLFVDPISI*

DNA sequence of casbene synthase (codon-optimized for E. coli expression)
ATGGCTCTACCCTCAGCGGCAATGCAAAGTAACCCGGAAAAATTGAACCTTTTCCACCGG
CTGAGCTCCCTGCCGACGACCAGCCTTGAATACGGCAACAACCGCTTTCCGTTTTTTTCTT
CTTCGGCTAAGTCTCACTTTAAGAAACCGACCCAGGCATGTCTGAGCTCGACGACCCACC
AGGAGGTTCGTCCGCTGGCCTACTTCCCTCCGACTGTTTGGGGTAACCGCTTCGCTAGCC
TGACCTTCAATCCGTCCGAATTCGAGAGCTATGATGAGCGCGTGATAGTGCTGAAGAAAA
AGGTCAAGGACATCCTGATCAGCTCTACGAGCGACAGCGTTGAAACCGTGATCCTAATCG
ACTTGCTCTGCCGCTTAGGTGTTTCTTACCATTTTGAAAATGACATCGAAGAACTGCTGTC
GAAAATTTTCAACAGCCAACCGGATTTGGTTGATGAAAAGGAGTGCGATCTGTACACCGC
GGCTATTGTGTTCCGCGTTTTCAGACAGCACGGTTTTAAAATGTCCTCCGATGTGTTTAGT
AAGTTTAAGGACAGCGATGGCAAGTTCAAAGAAAGCCTGAGAGGCGATGCAAAGGGTATG
CTGAGCCTGTTCGAGGCCAGCCACTTGTCCGTGCATGGTGAAGACATCTTGGAGGAGGC
GTTTGCCTTCACCAAGGACTACCTGCAATCCTCTGCGGTGGAATTGTTCCCCAACTTGAAG
CGTCACATCACCAATGCACTGGAGCAGCCGTTTCACAGCGGCGTGCCGCGTCTAGAGGC
GCGTAAATTTATCGACCTGTATGAAGCGGATATCGAATGCCGTAATGAGACCCTGCTGGA
GTTCGCCAAGCTGGATTACAACCGTGTTCAGCTGTTACATCAGCAAGAACTGTGCCAGTTT
AGCAAATGGTGGAAAGACTTAAATCTTGCTAGCGACATCCCGTATGCGCGTGATCGTATG
GCTGAGATCTTCTTTTGGGCAGTCGCTATGTATTTCGAGCCGGACTATGCCCATACCCGC
ATGATCATTGCAAAGGTGGTACTGCTGATTTCTTTGATTGATGATACCATTGATGCCTATGC
GACCATGGAAGAGACGCATATTCTGGCGGAAGCGGTGGCGCGTTGGGATATGAGCTGCT
TGGAGAAGCTGCCGGACTACATGAAAGTGATCTACAAATTGTTGCTCAACACCTTTTCGGA
ATTCGAGAAGGAGTTGACTGCGGAAGGTAAAAGCTACAGCGTGAAATATGGTCGTGAAGC
GTTCCAAGAGCTGGTTCGTGGCTACTATCTGGAGGCGGTTTGGCGTGATGAGGGGAAAAT
CCCGTCCTTCGATGACTACCTGTACAATGGTTCAATGACCACGGGTCTGCCACTGGTTTC
CACCGCATCCTTTATGGGTGTTCAAGAGATTACCGGCCTCAACGAGTTCCAGTGGCTGGA
GACAAACCCAAAATTGAGCTATGCGAGCGGTGCTTTCATTCGTCTAGTGAACGATCTGAC
CAGCCATGTTACCGAACAACAACGTGGCCACGTAGCGAGTTGTATTGACTGCTATATGAA
CCAGCATGGTGTTTCAAAGGACGAAGCAGTCAAAATCCTCCAAAAAATGGCTACCGACTG
TTGGAAAGAGATTAACGAAGAATGCATGCGTCAAAGCCAAGTCTCCGTCGGTCACCTGAT
GCGTATCGTGAATCTGGCGCGCCTCACTGACGTATCGTACAAATACGGCGACGGCTATAC
GGACAGCCAGCAGCTTAAGCAGTTCGTGAAGGGCCTGTTTGTTGACCCGATCAGCATTTA
A

Protein sequence of germacrene A synthase GAS from *Tanacetum parthenium* (accession number: F8UL80.1)

MAAVQATTGIQANTKTSAEPVRPLANFPPSVWGDRFLSFSLDKSEFERYAIAMEKPKEDVRKLI
VDSTMDSNEKLGLIYSVHRVGLTYMFLQEIESQLDKLFNEFSLQDYEEVDLYTISINFQVFRHLG
YKLPCDVFKKFKDAISGTFKESITSDVRGMLGLYESAQLRIRGEKILDEASVFIEGKLKSVVNTL
EGNLAQQVKQSLRRPFHQGMPMVEARLYFSNYEEECSSHDSLFKLAKLHFKYLELQQKEELR
IVTKWYKDMRFQETTPYIRDRVPEIYLWILGLYFEPRYSLARIIATKITLFLVVLDDTYDAYATIEEI
RLLTDAMNKWDISAMEQIPEYIRPFYKVLLDEYAEIGKRMAKEGRADTVIASKEAFQDIARGYLE
EAEWTNSGYVASFPEYMKNGLITSAYNVISKSALVGMGEIVSEDALAWYESHPKPLQASELISR
LQDDVMTYQFERERGQSATGVDAYIKTYGVSEKKAIDELKIMIENAWKDINEGCLKPRQVSMD
LLAPILNLARMIDVVYRYDDGFTFPGSTLKEYINLLFVDSLPV*

DNA sequence of gas (codon-optimized for E. coli expression)

ATGGCGGCAGTACAAGCTACTACAGGAATACAAGCCAACACCAAGACCAGCGCGGAACC
GGTTCGTCCACTGGCGAACTTCCCGCCTTCCGTTTGGGGTGATCGTTTTCTGAGCTTCAG
CTTGGACAAATCGGAATTCGAGCGTTACGCCATTGCTATGGAAAAACCGAAAGAGGACGT
TCGTAAACTGATCGTGGATTCTACGATGGACAGCAACGAAAAGCTGGGCCTCATCTATAG
CGTTCACCGCGTGGGTCTCACGTACATGTTTTTGCAAGAGATCGAAAGCCAGCTGGATAA
GCTGTTCAACGAGTTCTCATTGCAGGATTATGAGGAGGTTGACCTGTATACCATTTCTATT
AATTTTCAGGTGTTTCGTCACCTGGGCTACAAACTTCCGTGCGATGTGTTCAAGAAATTCA
AGGACGCCATCAGTGGCACCTTCAAAGAGAGCATTACGTCCGACGTTCGCGGTATGCTG
GGTTTGTACGAAAGTGCGCAGCTGCGTATTCGTGGCGAAAAGATCCTTGACGAGGCGTCC
GTCTTCATCGAGGGCAAACTGAAGAGCGTTGTAAATACCCTGGAAGGCAACCTGGCTCAG
CAGGTGAAACAGAGCCTGCGTCGTCCGTTTCATCAAGGTATGCCGATGGTTGAAGCGCG
CCTGTACTTTTCTAACTATGAGGAAGAGTGTAGCAGCCATGATAGCTTGTTCAAGCTCGCA
AAATTACACTTTAAGTATTTGGAACTGCAACAGAAGGAGGAGCTGCGGATTGTCACCAAAT
GGTATAAGGACATGCGTTTCCAAGAAACCACCCCGTATATCCGCGATCGCGTGCCGGAGA
TTTATCTGTGGATCCTGGGTTTGTACTTCGAGCCGCGTTACTCTCTGGCGCGCATCATCG
CTACCAAGATCACGCTGTTTTTGGTTGTGCTGGACGACACGTACGATGCGTACGCGACCA
TTGAGGAGATTCGCCTGCTGACCGATGCAATGAATAAATGGGATATCTCGGCAATGGAGC
AAATTCCGGAATATATTAGGCCATTTTATAAAGTCCTGTTAGACGAATATGCTGAAATTGGC
AAACGTATGGCAAAAGAGGGCCGTGCAGATACGGTCATTGCGAGCAAAGAGGCATTTCAA
GATATCGCGAGAGGCTACTTGGAAGAAGCTGAGTGGACCAATTCCGGTTACGTGGCGAG
CTTCCCGGAATATATGAAGAACGGTCTGATCACCTCTGCCTACAATGTTATTAGCAAAAGC
GCTCTGGTTGGTATGGGTGAAATCGTGTCCGAGGATGCGCTGGCGTGGTACGAATCCCA
CCCGAAGCCGCTGCAAGCGAGCGAGCTGATCTCTCGACTGCAAGACGACGTAATGACCT
ACCAGTTCGAACGTGAACGCGGTCAGTCCGCGACTGGCGTGGATGCGTATATTAAAACCT
ATGGCGTCTCCGAGAAGAAGGCGATCGATGAGCTTAAAATTATGATCGAGAACGCATGGA
AAGACATCAACGAAGGGTGCCTGAAGCCGCGTCAGGTTAGCATGGACTTGTTAGCCCCTA
TTTTGAATCTCGCTCGTATGATCGACGTCGTGTATCGTTACGACGATGGTTTTACCTTTCC
GGGTAGCACTCTGAAGGAGTACATCAACCTGCTGTTCGTGGACAGCCTTCCGGTTTAA

Table S1: Plasmids and strains used in this study.

Plasmids/Strains	Descriptions	Source
**plasmids**		
pET28a-*cs*	pET28a-derived plasmid containing gene *cs* coding casbene synthase from *Ricinus communis* for producing casbene	Genscript
pET28a-*gas*	pET28a-derived plasmid containing gene *gas* coding germacrene A synthase from *Tanacetum parthenium* for producing germacrene A	Genscript
pXT02007	pCDFDuet-1 derived plasmid for producing FPP, contained genes *Ecthim*, *Mjipk, idi, ispA* in multiple cloning site-2	[5]
pXT02013	pCDFDuet-1 derived plasmid for producing GGPP, contained genes *Ecthim*, *Mjipk, idi, ispA, crtE* in multiple cloning site-2	[6]
**Bacterial strains**		
E. coli Trans1-T1	*E. coli* host for general cloning	Beijing TransGen Biotech Co., Ltd
E. coli T7	*E. coli* host for protein expression	Beijing TransGen Biotech Co., Ltd
XT02019	*E. coli* T7 with plasmids pXT02007 and pET28a-*gas*, for producing germacrene A	This study
XT02020	*E. coli* T7 with plasmids pXT02013 and pET28a-*cs*, for producing casbene	This study

Table S2: Screening the conditions for the allylic oxidation of casbene (**4**).[a]

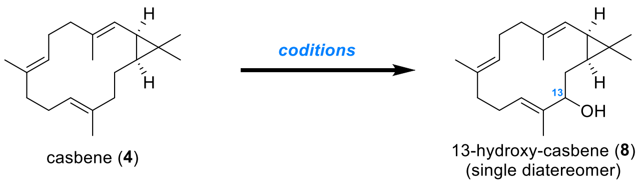

Entry	conditions	Results[b]
**1**[7]	Na_2CrO_4, Ac_2O/AcOH, NaOAc, C_6H_6, 60 °C	complex mixtures
**2**[8]	$Na_2Cr_2O_7$, NOS, acetone, 50 °C	complex mixtures
**3**[9]	PCC, NaOAc, Celite, C_6H_6, 95 °C	complex mixtures
**4**[10]	PDC, TBHP, C_6H_6, rt	complex mixtures
**5**[11]	$Cr(CO)_6$, CH_2Cl_2, rt	complex mixtures
**6**[12]	CrO_3, AcOH, rt	complex mixtures
**7**	CrO_3, 3,5-DMP, CH_2Cl_2, −20 °C	complex mixtures
**8**[13]	4CzIPN, NHPI, 456 nm, CH_3CN/H_2O, −20 °C	complex mixtures
**9**[13]	4CzIPN, O_2, 456 nm, CH_3CN/H_2O, −20 °C	complex mixtures
**10**[13]	Na_2-eosin Y, O_2, 456 nm, CH_3CN/H_2O, −20 °C	complex mixtures
**11**[14]	CuBr, TBHP, C_6H_6, 60 °C	no reaction
**12**[15]	CuI, TBHP, CH_3CN, 50 °C	no reaction
**13**[16]	PIFA, TBHP, Cs_2CO_3, O_2, EtOAc, −15 °C	no reaction
**14**[17]	$Pd(OH)_2$, TBHP, Cs_2CO_3, CH_2Cl_2, rt	no reaction
**15**[18]	$Co(acac)_2$, NHPI, O_2, CH_3CN, 50 °C	no reaction
**16**[19]	$Rh_2(cap)_4$, TBHP, K_2CO_3, CH_2Cl_2, rt	no reaction
**17**[20]	$Mn(OAc)_3$, TBHP, O_2, $CH_3COOCH_2CH_3$, rt	no reaction
**18**[21]	$RuCl_3$, TBHP, $ClCH_2CH_2Cl$, rt	no reaction
**19**	SeO_2 (1.2 equiv), CH_2Cl_2, rt	16%
**20**	SeO_2 (0.5 equiv), TBHP (0.6 equiv), CH_2Cl_2, rt	15%
**21**	SeO_2 (0.1 equiv), TBHP (1.0 equiv), pyridine (1.0 equiv), CH_2Cl_2, rt	12%
**22**	SeO_2 (0.1 equiv), TBHP (1.0 equiv), Na_2HPO_4 (1.0 equiv), CH_2Cl_2, rt	13%
**23**	SeO_2 (0.1 equiv), TBHP (1.0 equiv), CH_2Cl_2, rt	19%
**24**	SeO_2 (0.1 equiv), TBHP (1.0 equiv), 1,4-dioxane, rt	12%
**25**	SeO_2 (0.1 equiv), TBHP (1.0 equiv), Ac_2O, rt	5%
**26**	SeO_2 (0.1 equiv), TBHP (1.0 equiv), CH_3CN, rt	13%
**27**	SeO_2 (0.1 equiv), TBHP (1.0 equiv), toluene, rt	13%
**28**[c]	SeO_2 (0.1 equiv), TBHP (1.0 equiv), $ClCH_2CH_2Cl$, 45°C	6%
**29**	SeO_2 (0.1 equiv), TBHP (3 × 0.2 equiv), CH_2Cl_2, rt	33%
**30**[d]	SeO_2 (0.1 equiv), TBHP (3 × 0.2 equiv), CH_2Cl_2, 0 °C	48%; [39% (79% brsm)][e]
**31**[f]	SeO_2 (0.1 equiv), TBHP (3 × 0.2 equiv), CH_2Cl_2, −20 °C	26%
**32**	SeO_2 (0.1 equiv), TBHP (5 × 0.2 equiv), CH_2Cl_2, 0 °C	27%

[a]General conditions: 0.02 mmol of casbene (**4**) in indicated solvent (2.0 mL) was treated with indicated oxidant under air for 1 h; [b]isolated yield; [c]reaction for 15 min; [d]reaction for 12 h; [e]gram-scale yield; [f]reaction for 18 h.

Table S3: ^{1}H NMR data (in $CDCl_3$) of depressin (**1**) in comparison with those of reported.

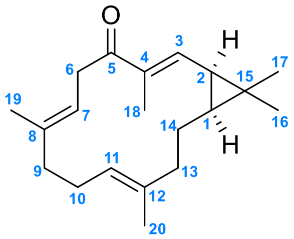

depressin

No.	δ_H, multi (J in Hz)^a Isolated[22]	δ_H, multi (J in Hz)^b Synthetic[23]	δ_H, multi (J in Hz)^b Our synthetic sample
**1**	1.15, m	1.14, m	1.14, m
**2**	1.50, dd (10.2, 8.7)	1.49, dd (10.2, 8.7)	1.49, dd (10.0, 8.7)
**3**	6.37, d (10.2)	6.38, dq (1.3, 10.2)	6.37, d (10.2)
**4**	--	--	--
**5**	--	--	--
**6a**	3.55, dd (13.8, 5.7)	3.55, dd (13.9, 8.6)	3.55, dd (13.8, 8.6)
**6b**	2.97, dd (13.8, 5.7)	2.98, dd (13.8, 5.7)	2.98, dd (13.7, 5.5)
**7**	5.08, t (6.6)	5.07, ddt (8.7, 5.9, 1.4)	5.07, td (6.8, 1.4)
**8**	--	--	--
**9a**	2.15, m	2.09, m	2.09, m
**9b**	2.00, m	2.00, m	2.00, m
**10a**	2.17, m	2.16, m	2.16, m
**10b**	1.96, m	1.98, m	1.98, m
**11**	4.84, t (5.4)	4.84, dd (9.0, 5.1)	4.84, dd (8.4, 5.1)
**12**	--	--	--
**13a**	2.20, m	2.20, d (12.8)	2.20, m
**13b**	1.75, m	1.75, ddd (12.8, 9.9, 2.9)	1.75, ddd (12.8, 9.9, 2.9)
**14a**	2.05, m	2.06, m	2.06, m
**14b**	0.80, m	0.86, dddd (13.8, 12.6, 9.6, 2.9)	0.86, dddd (14.8, 12.4, 9.6, 2.9)
**15**	--	--	--
**16**	1.16, s	1.16, s	1.16, s
**17**	1.09, s	1.09, s	1.09, s
**18**	1.87, s	1.87, d (1.3)	1.87, d (1.1)
**19**	1.56, s	1.57, t (1.2)	1.57, br s
**20**	1.56, s	1.56, s	1.56, s

^{a}400 MHz; ^{b}600 MHz.

Table S4: ^{13}C NMR data (in $CDCl_3$) of depressin (**1**) in comparison with those of reported.

No.	δ_C^a Isolated[22]	δ_C^b Synthetic[23]	δ_C^b Our synthetic sample
**1**	35.2	35.2	35.3
**2**	27.6	27.7	27.7
**3**	143.1	143.2	143.2
**4**	136.6	136.6	136.7
**5**	199.9	199.9	199.9
**6**	39.4	39.4	39.4
**7**	119.4	119.4	119.4
**8**	137.1	137.1	137.2
**9**	39.0	39.0	39.1
**10**	23.9	23.9	23.9
**11**	124.4	124.4	124.4
**12**	135.9	135.9	135.9
**13**	39.9	39.9	39.9
**14**	26.3	26.3	26.3
**15**	25.4	25.4	25.4
**16**	29.0	29.0	29.0
**17**	15.8	15.9	15.9
**18**	11.6	11.6	11.7
**19**	15.6	15.6	15.6
**20**	15.3	15.3	15.3

^a100 MHz; ^b150 MHz.

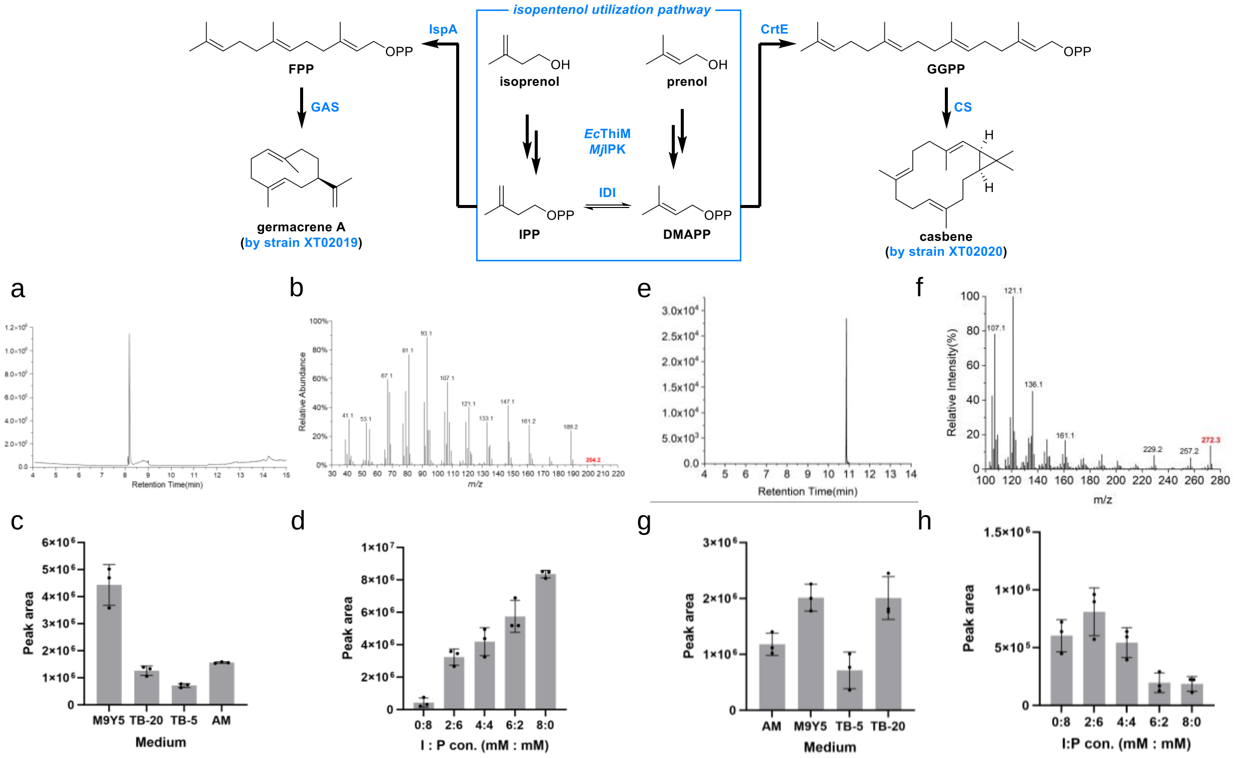

Figure S1. Production of casbene (**4**) and germacrene A (**5**) using the isopentenol utilization pathway. a) Gas chromatogram of the EtOAc extract of the fermentation for producing germacrene A. b) EI mass spectrum of germacrene A. c) Effect of the fermentation medium on the production of germacrene A. d) Effect of the ratio of isoprenol and prenol on the production of germacrene A. e) Gas chromatogram of the EtOAc extract of the fermentation for producing casbene. f) EI mass spectrum of casbene. g) Effect of the fermentation medium on the production of casbene. h) Effect of the ratio of isoprenol and prenol on the production of casbene.

Figure S2. ¹H NMR spectrum of casbene (4, 400 MHz, $CDCl_3$)

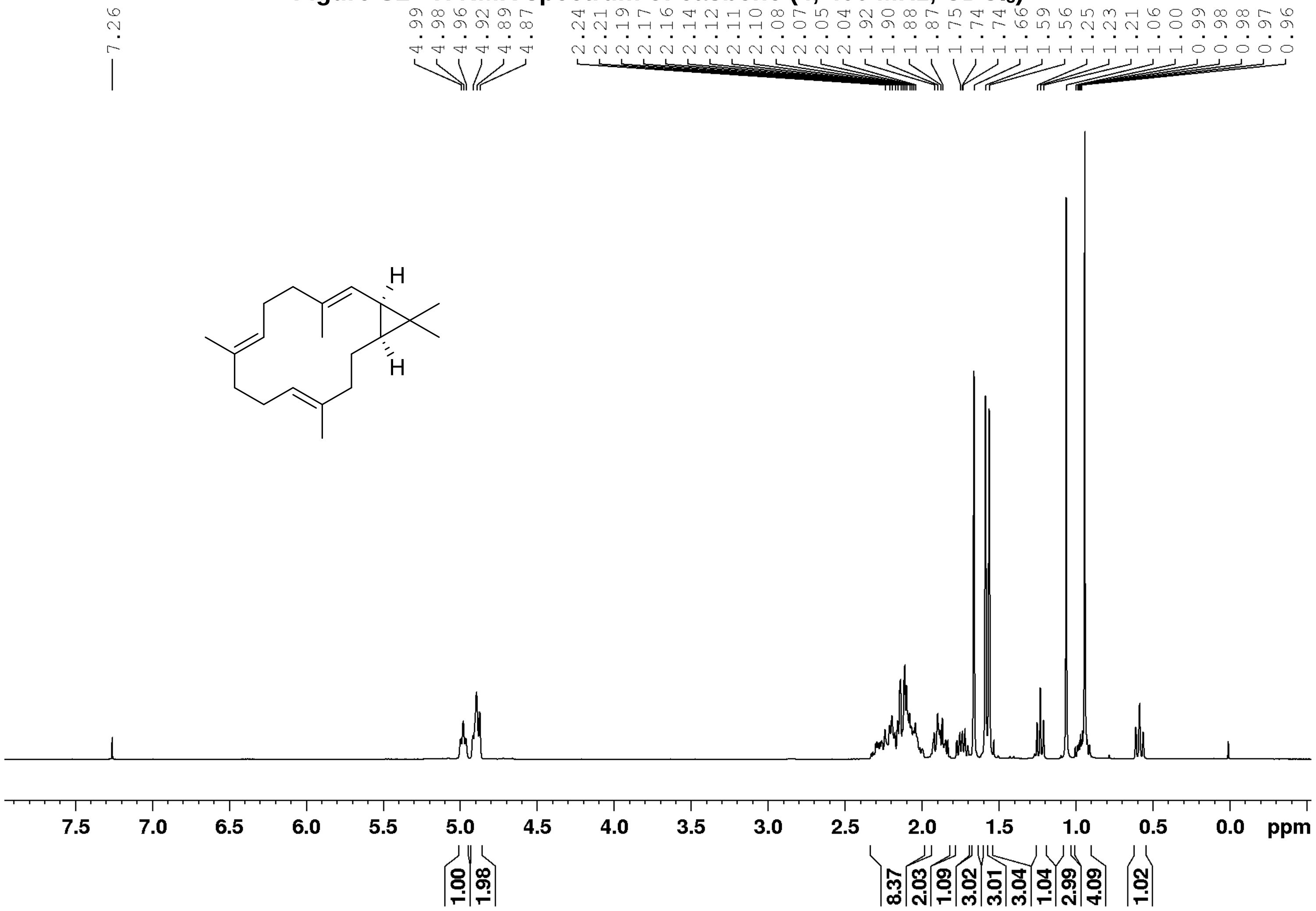

Figure S3. ^{13}C NMR spectrum of casbene (4, 100 MHz, $CDCl_3$)

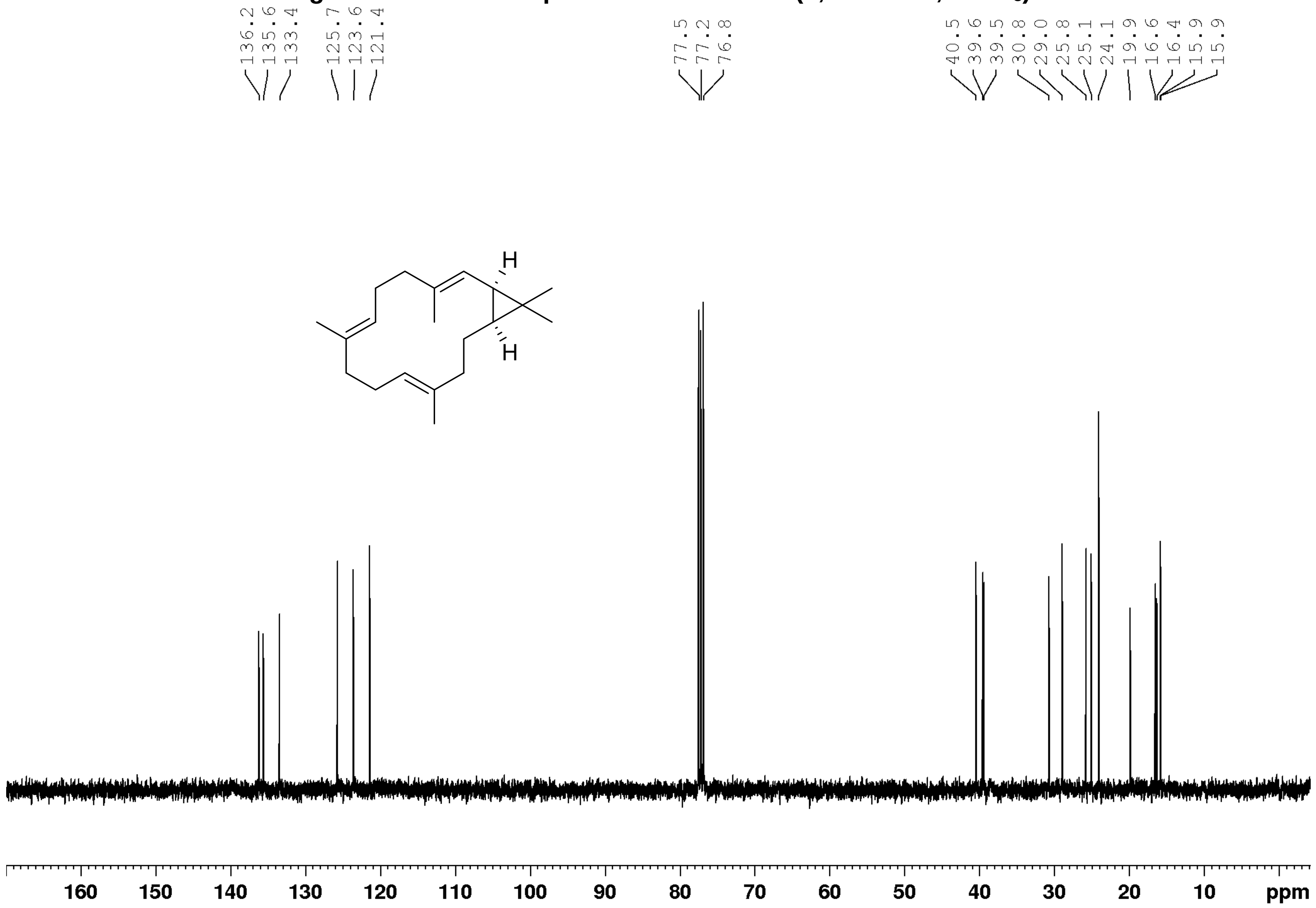

Figure S4. ^{1}H NMR spectrum of casbene (4, 600 MHz, C_6D_6)

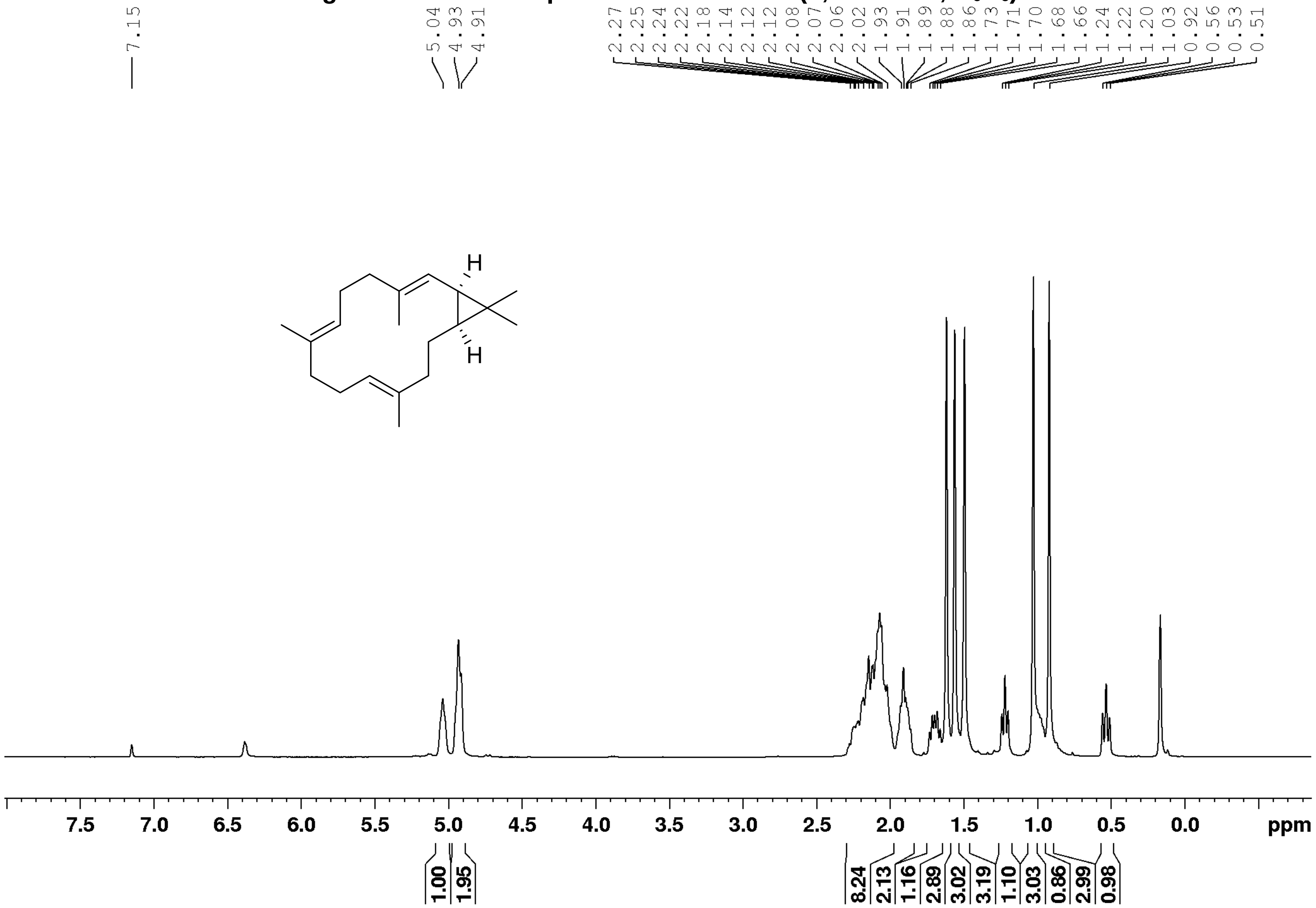

Figure S5. ^{13}C NMR spectrum of casbene (4, 150 MHz, C_6D_6)

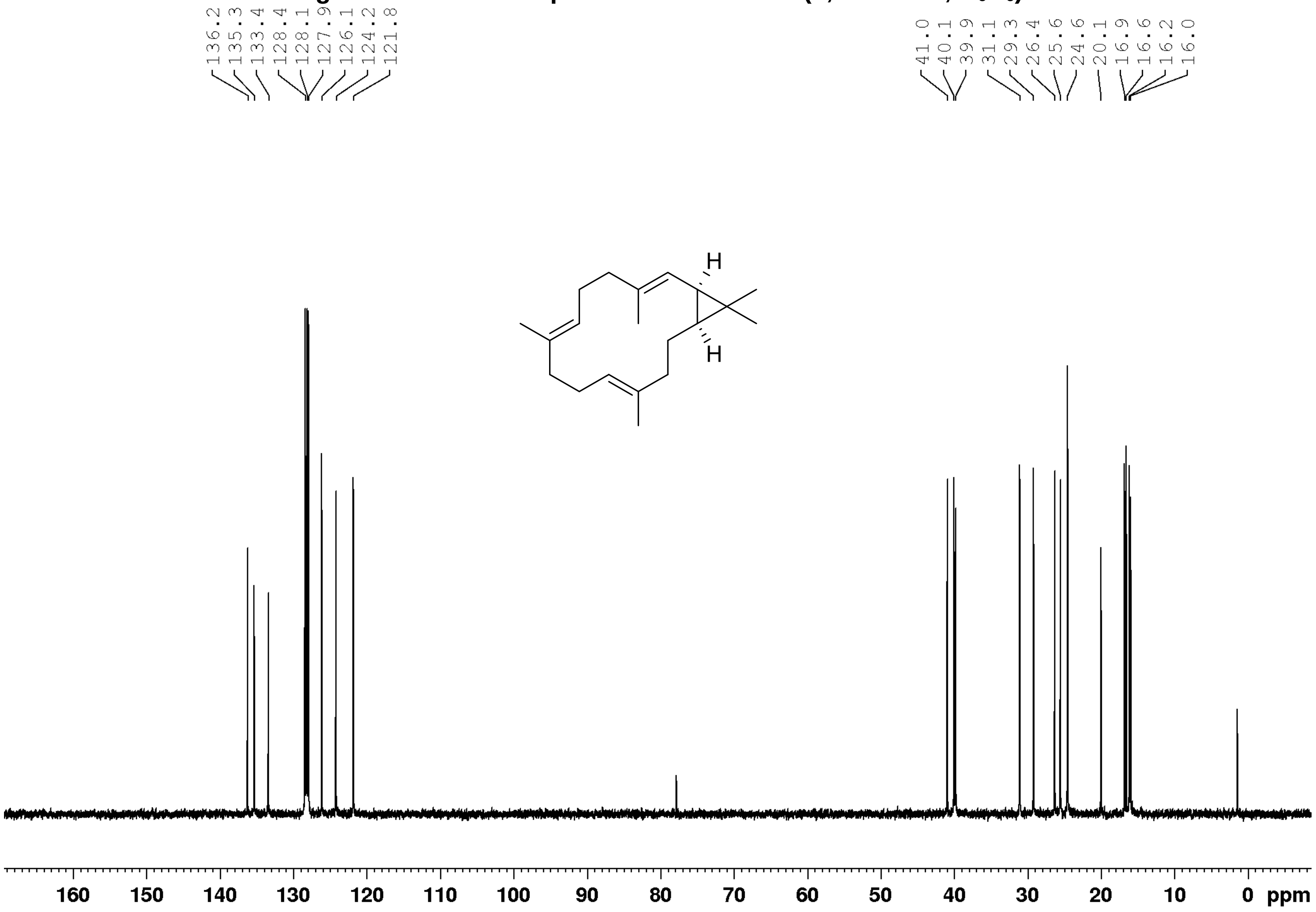

Figure S6. ¹H NMR spectrum of compound 8 (400 MHz, $CDCl_3$)

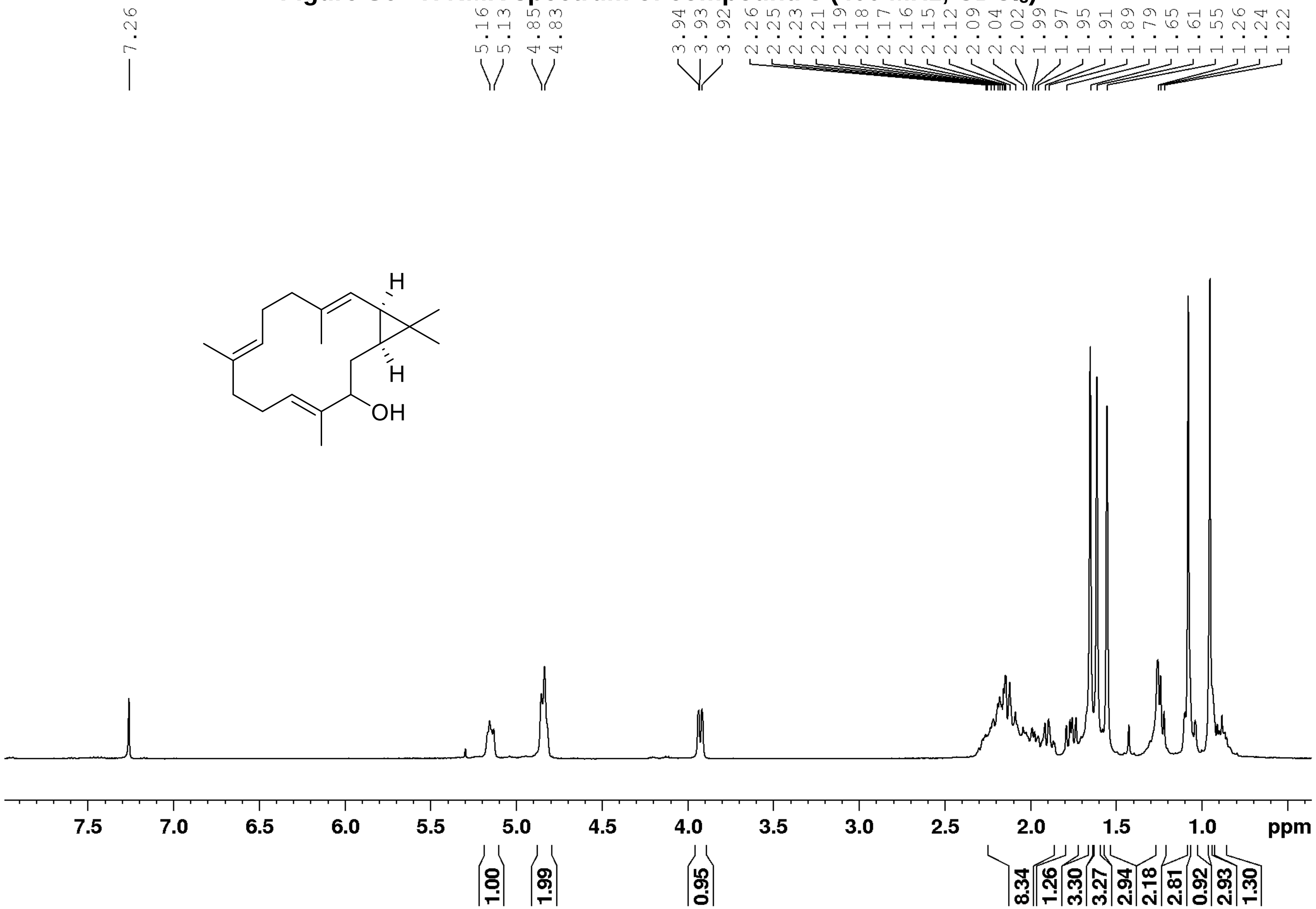

Figure S7. ^{13}C NMR spectrum of compound 8 (100 MHz, $CDCl_3$)

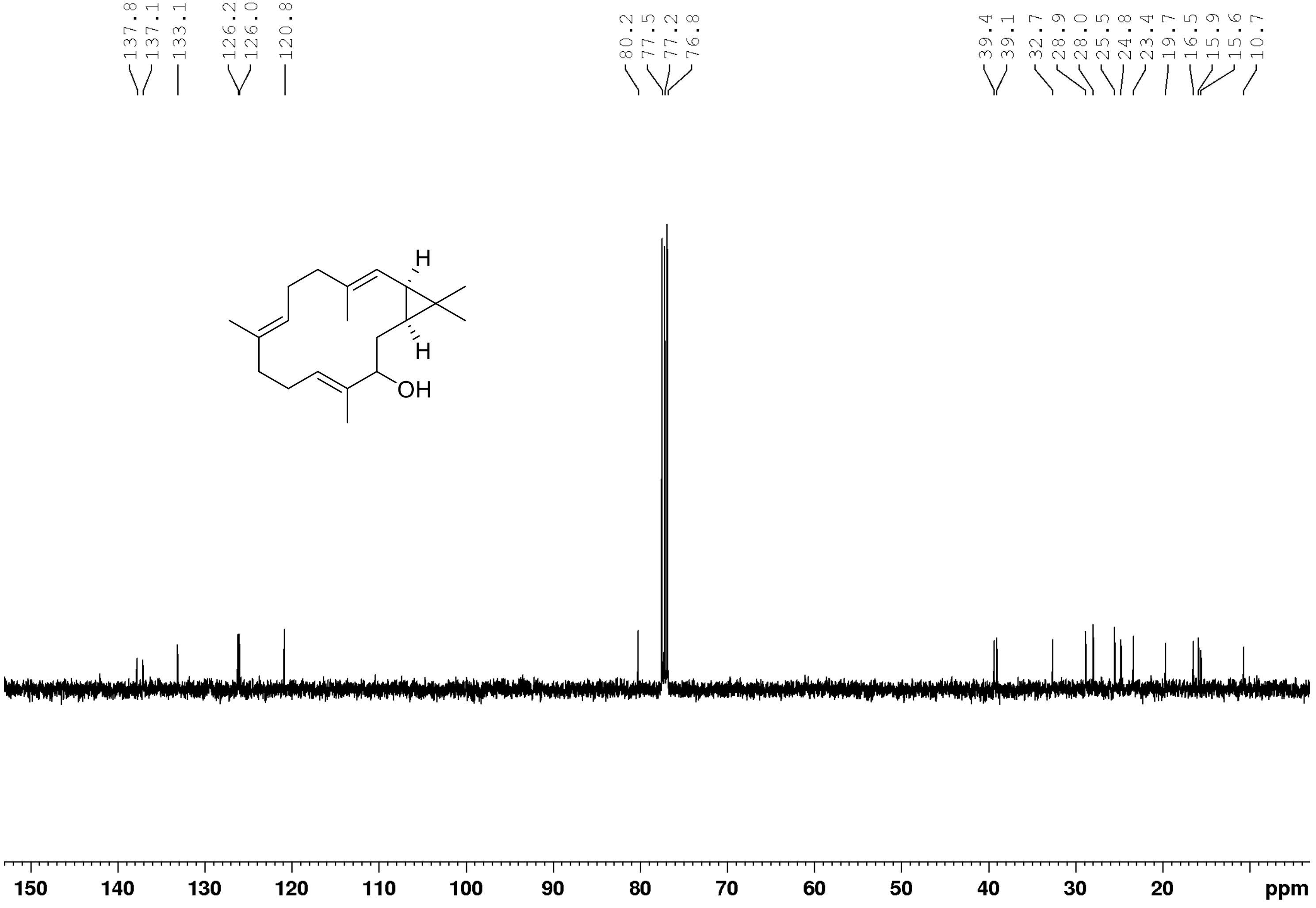

Figure S8. ^1H NMR spectrum of compound 9 (400 MHz, $CDCl_3$)

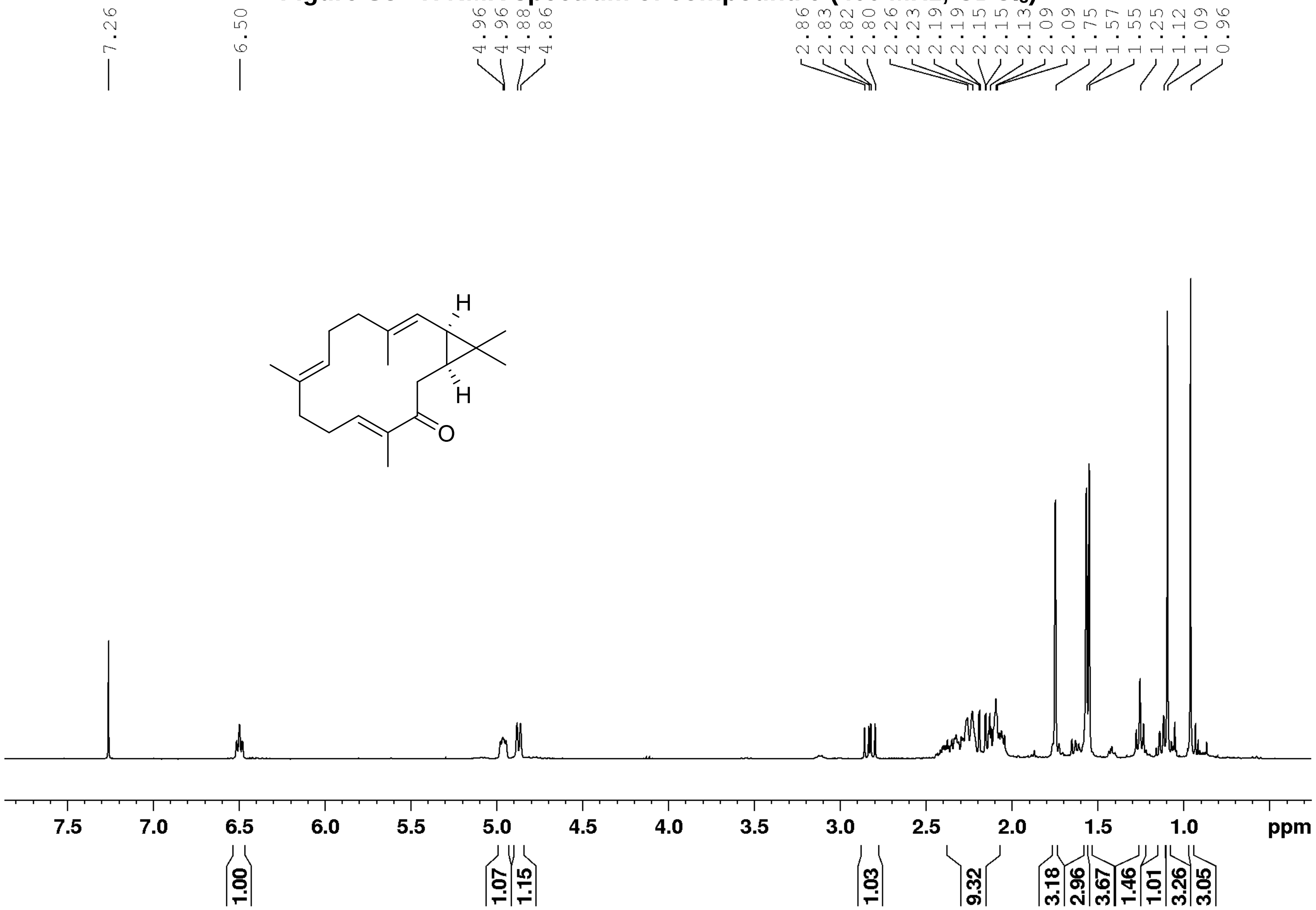

Figure S9. ^{13}C **NMR spectrum of compound 9 (100 MHz,** $CDCl_3$**)**

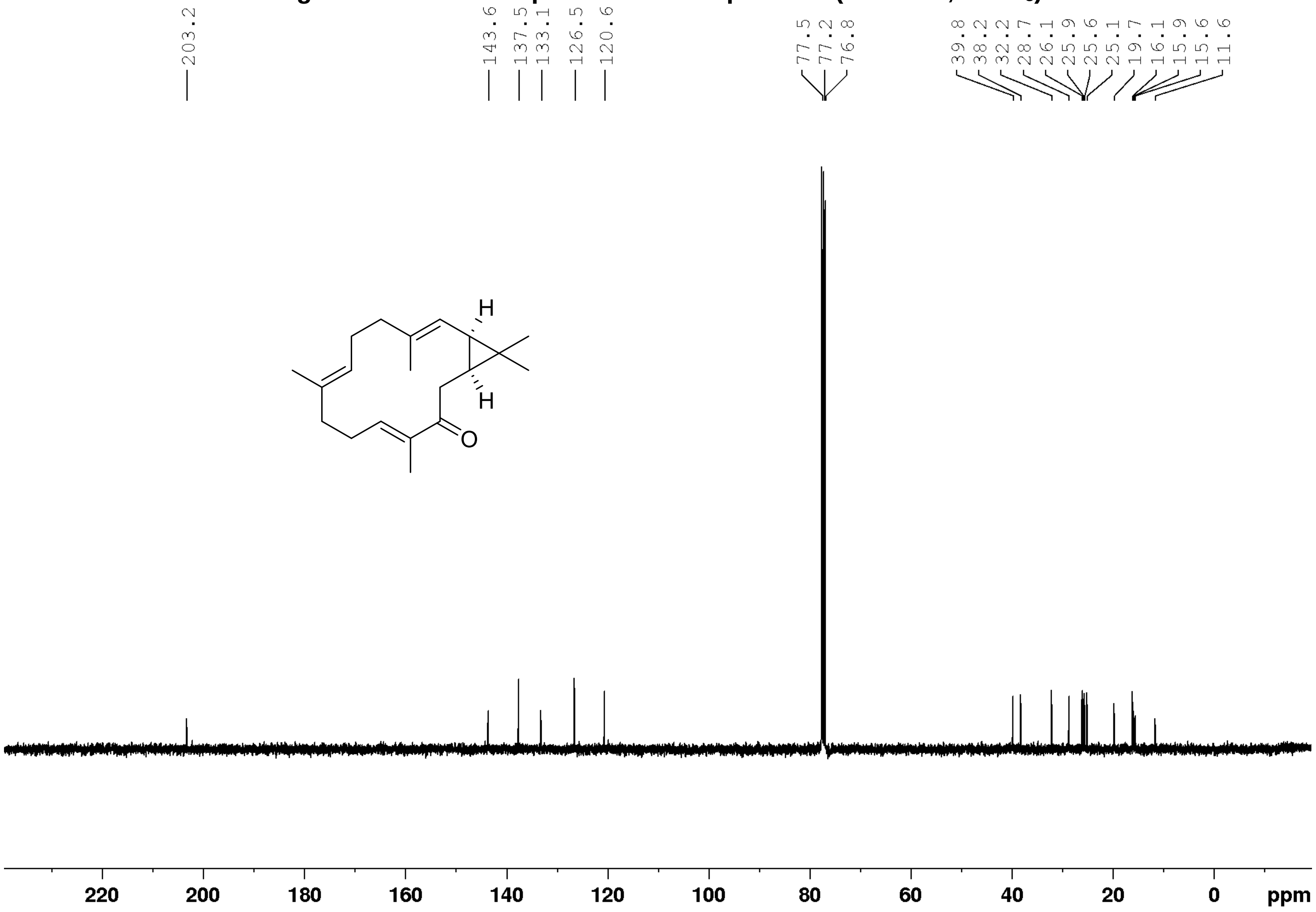

Figure S10. ^{1}H NMR spectrum of compound 16 (400 MHz, CDCl$_3$)

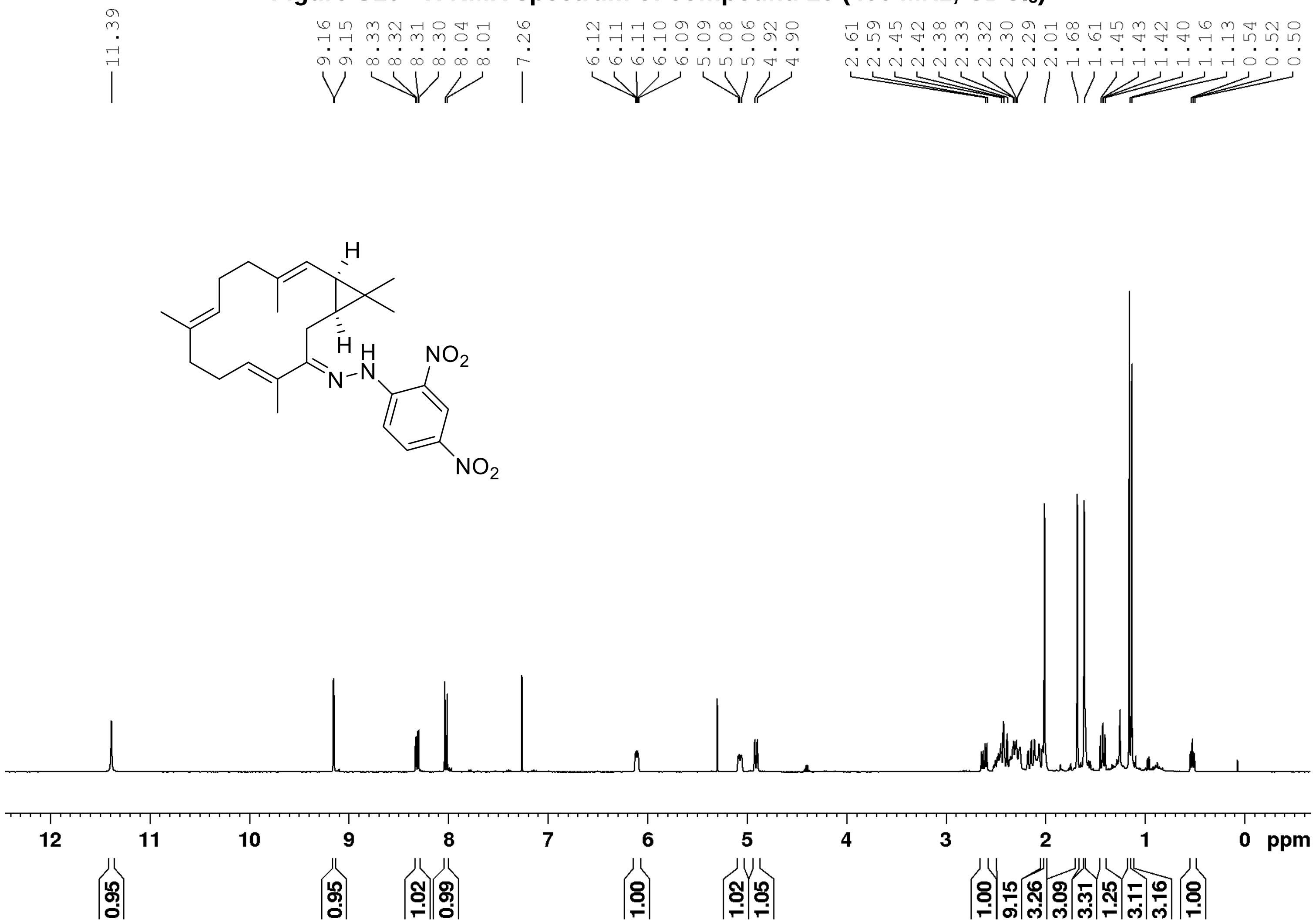

Figure S11. ^{13}C NMR spectrum of compound 16 (100 MHz, $CDCl_3$)

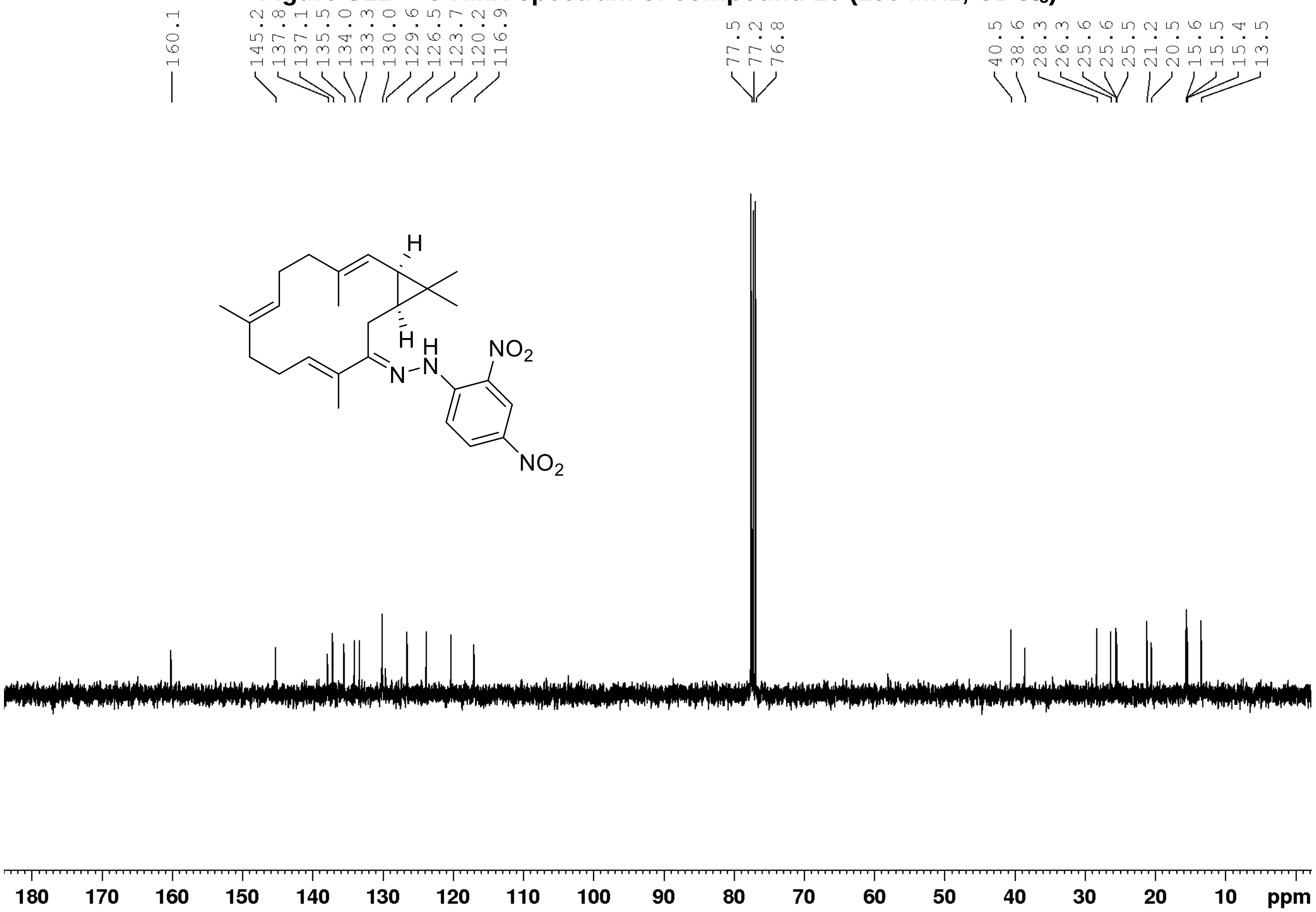

Figure S12. ^{1}H NMR spectrum of 5-hydroxy-13-ketocasbene (11, 400 MHz, $CDCl_3$)

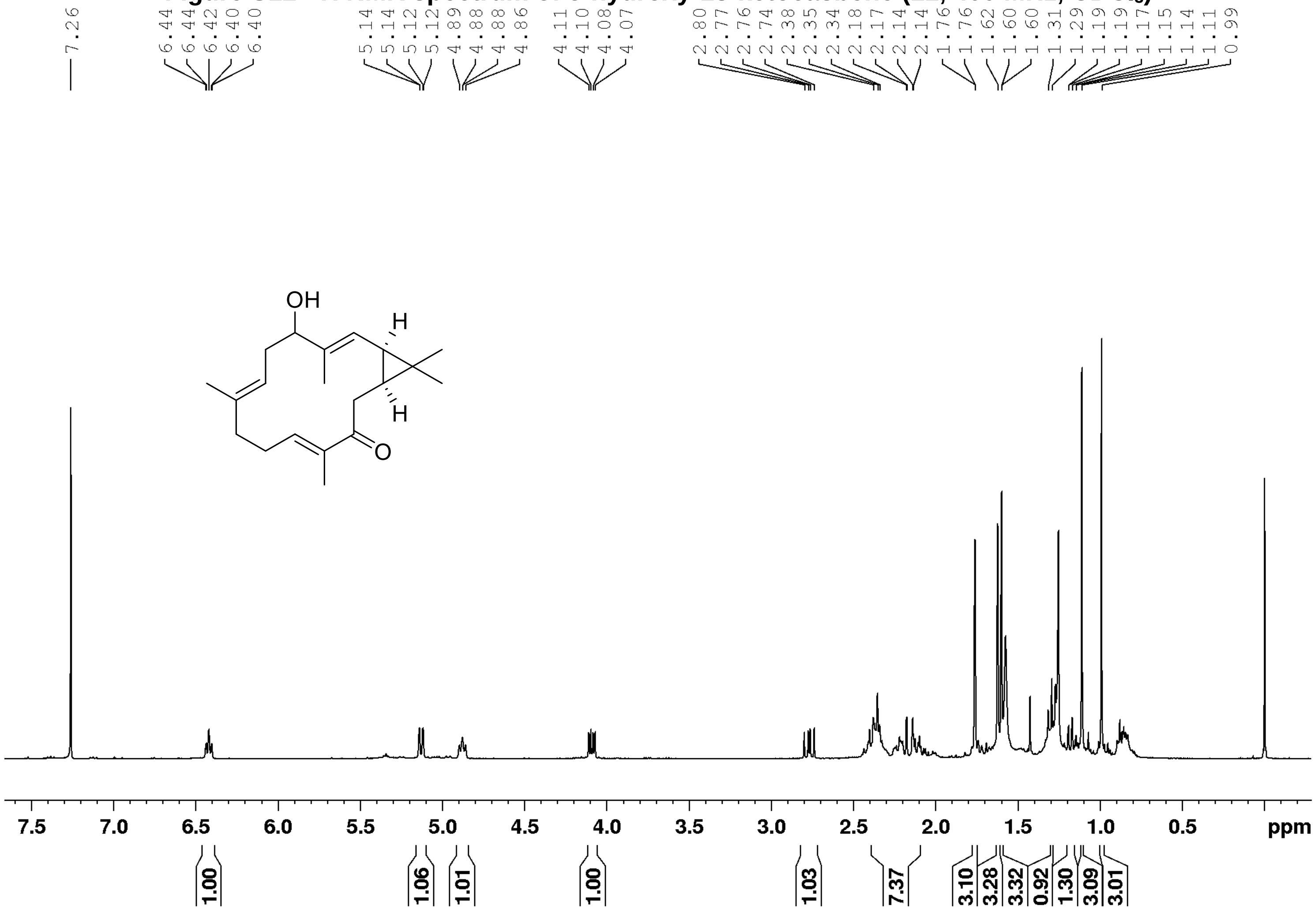

Figure S13. ^{13}C NMR spectrum of 5-hydroxy-13-ketocasbene (11, 100 MHz, $CDCl_3$)

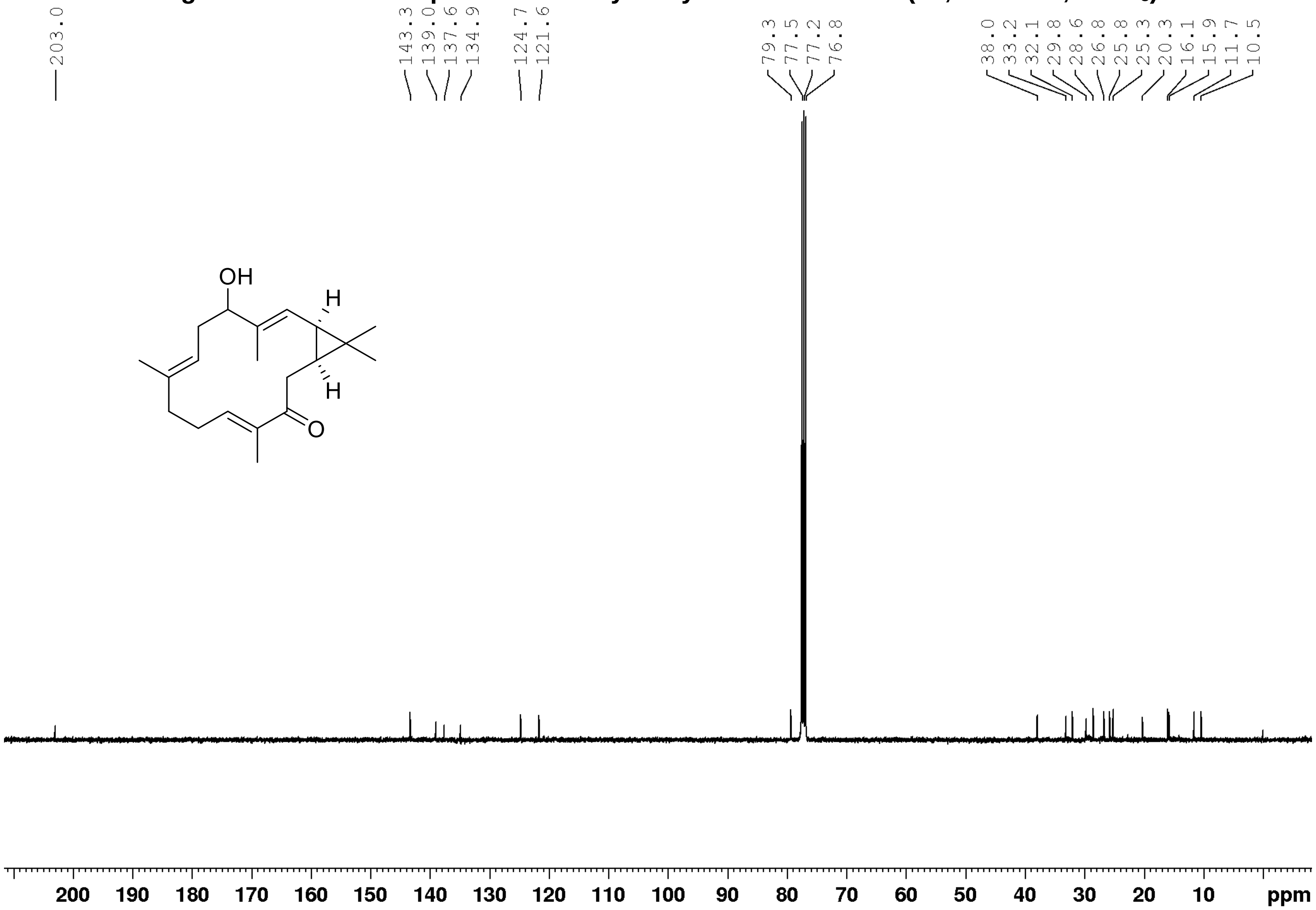

Figure S14. $^1H,^1H$ COSY spectrum of 5-hydroxy-13-ketocasbene (11, 400 MHz, $CDCl_3$)

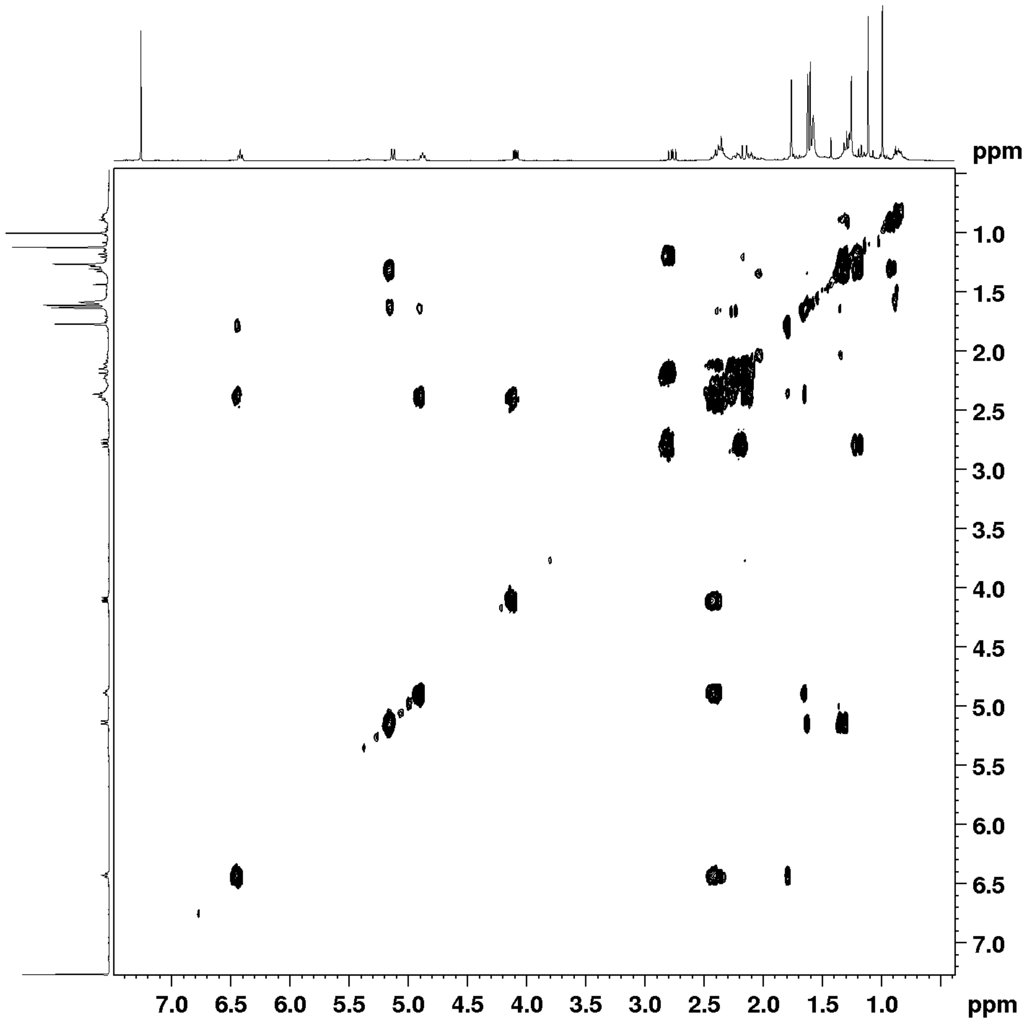

Figure S15. HSQC spectrum of 5-hydroxy-13-ketocasbene (11, 100 MHz, $CDCl_3$)

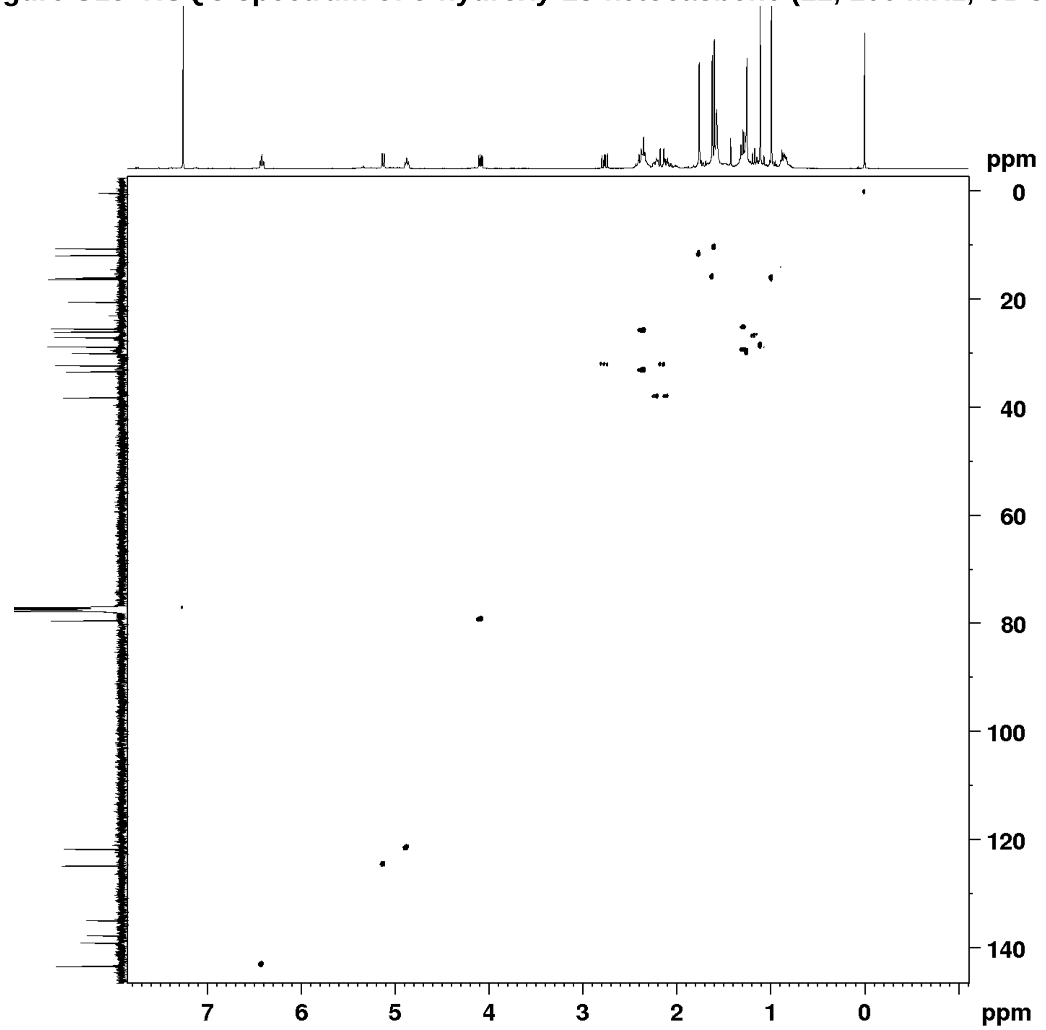

Figure S16. HMBC spectrum of 5-hydroxy-13-ketocasbene (11, 100 MHz, $CDCl_3$)

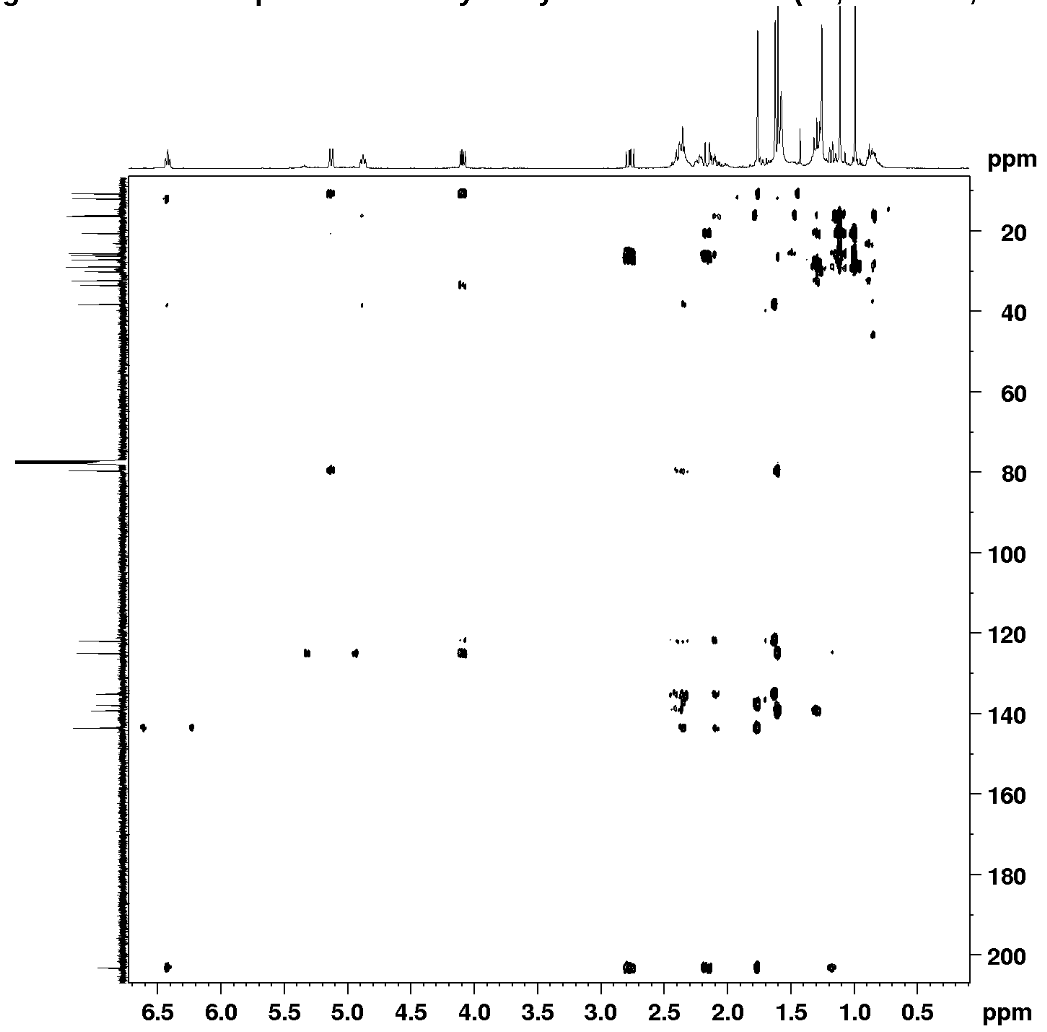

Figure S17. ^{1}H NMR spectrum of 9-hydroxy-13-ketocasbene (10, 400 MHz, $CDCl_3$)

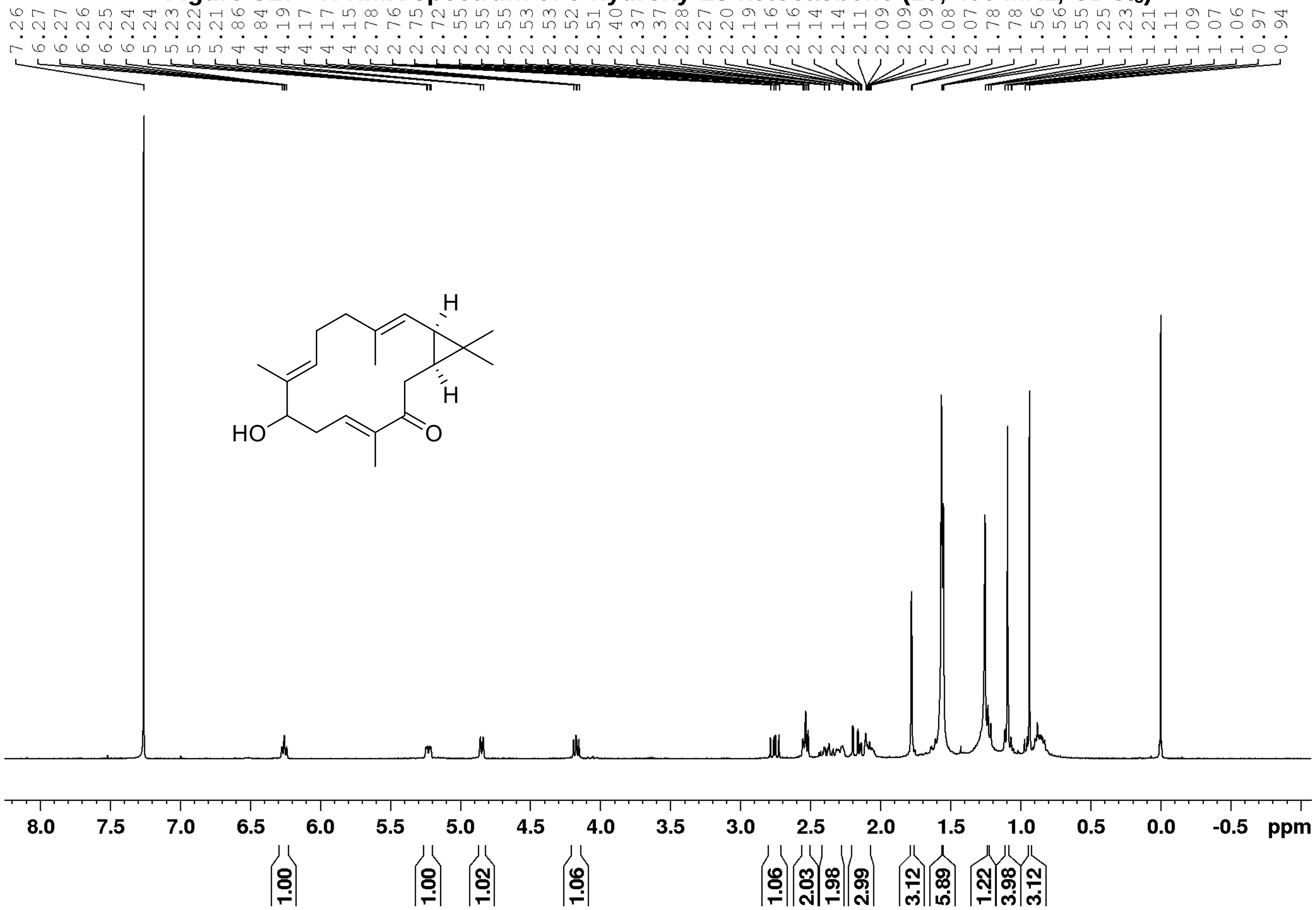

Figure S18. ^{13}C NMR spectrum of 9-hydroxy-13-ketocasbene (10, 100 MHz, $CDCl_3$)

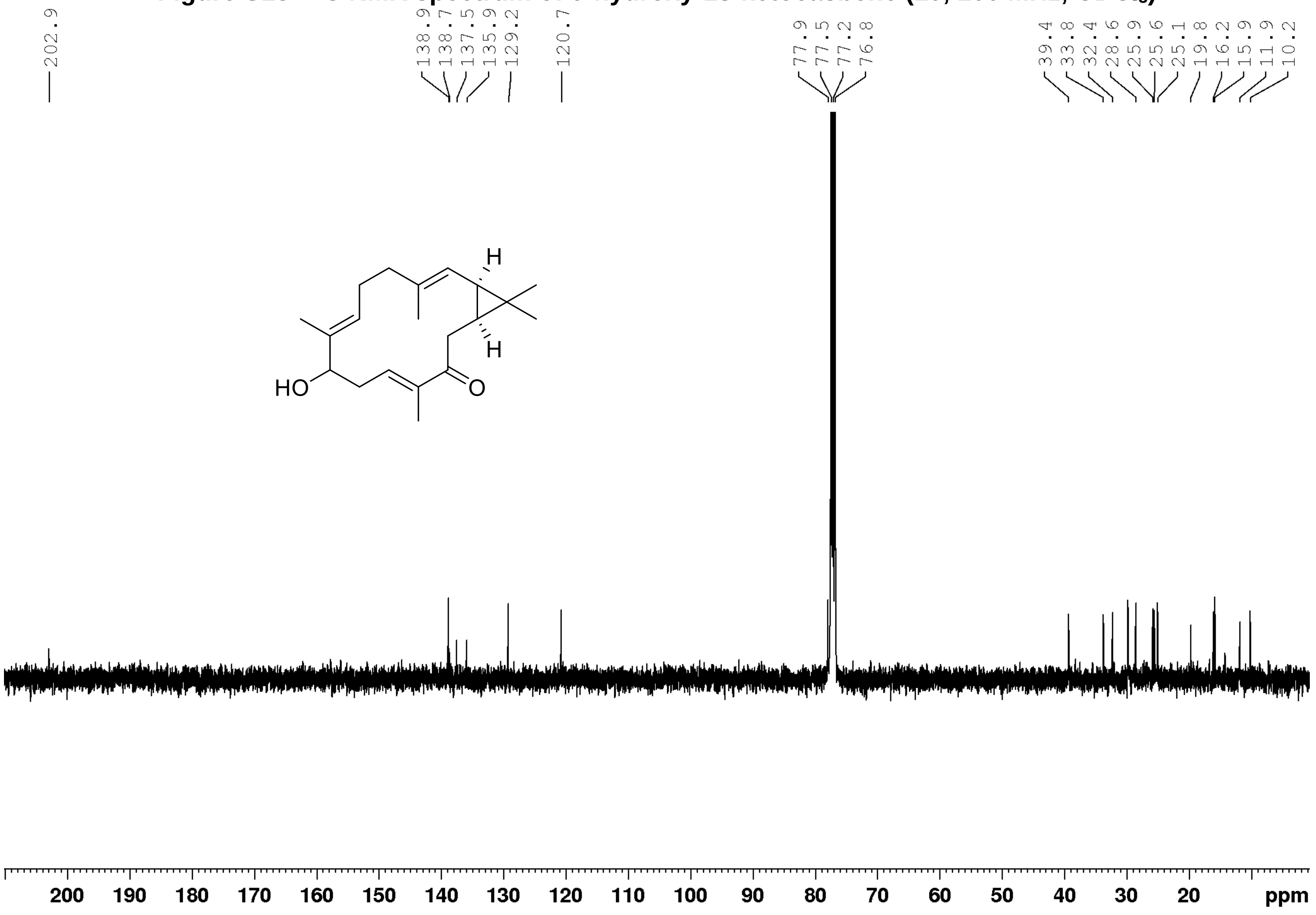

Figure S19. ^1H-^1H COSY spectrum of 9-hydroxy-13-ketocasbene (10, 400 MHz, $CDCl_3$)

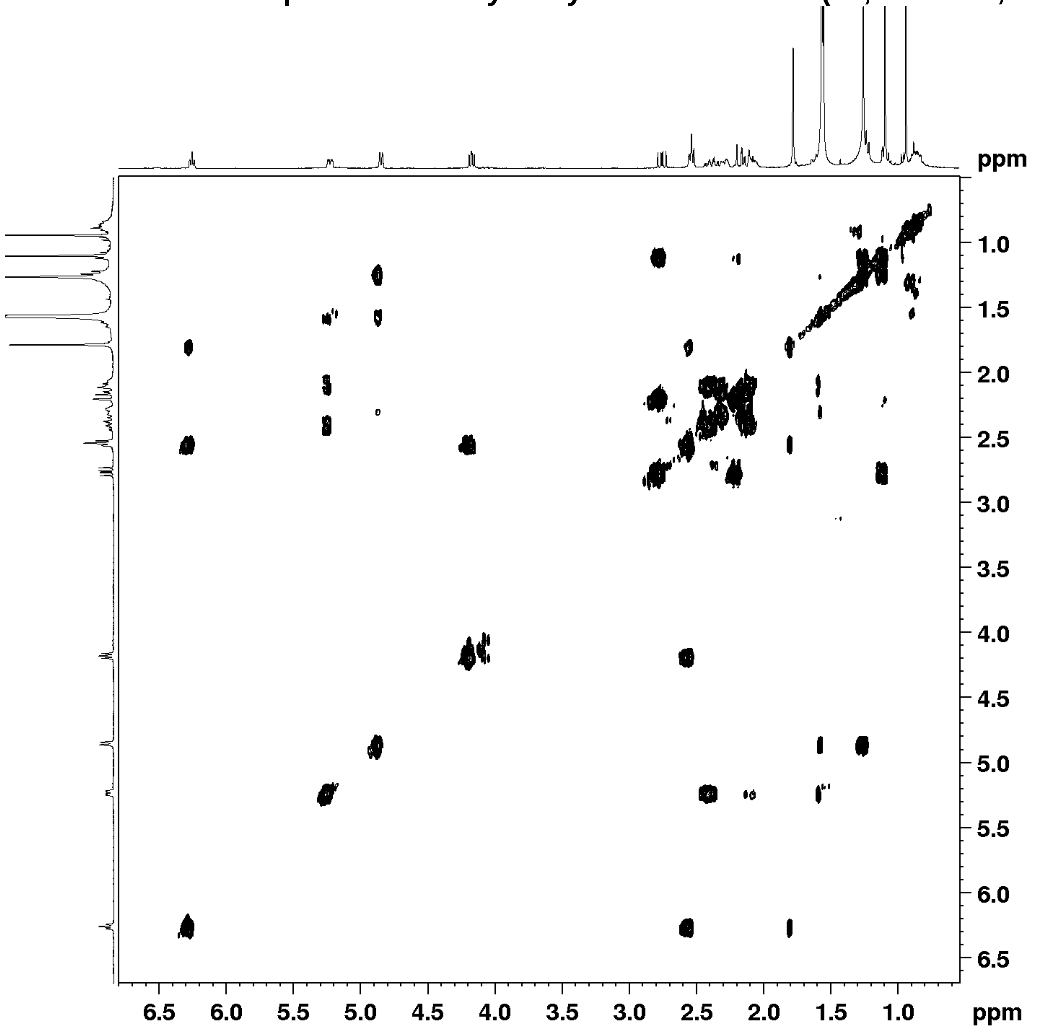

Figure S20. HSQC spectrum of 9-hydroxy-13-ketocasbene (10, 100 MHz, $CDCl_3$)

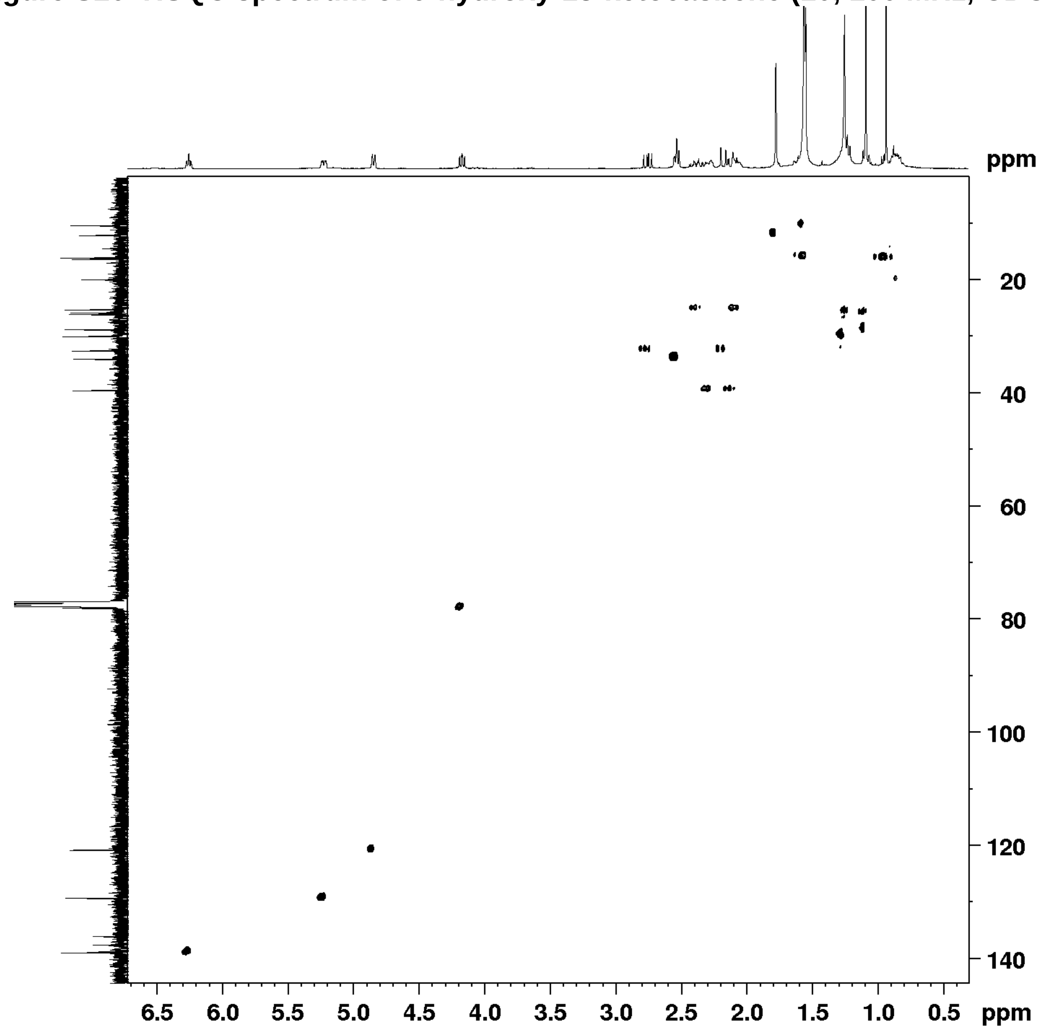

Figure S21. HMBC spectrum of 9-hydroxy-13-ketocasbene (10, 100 MHz, $CDCl_3$)

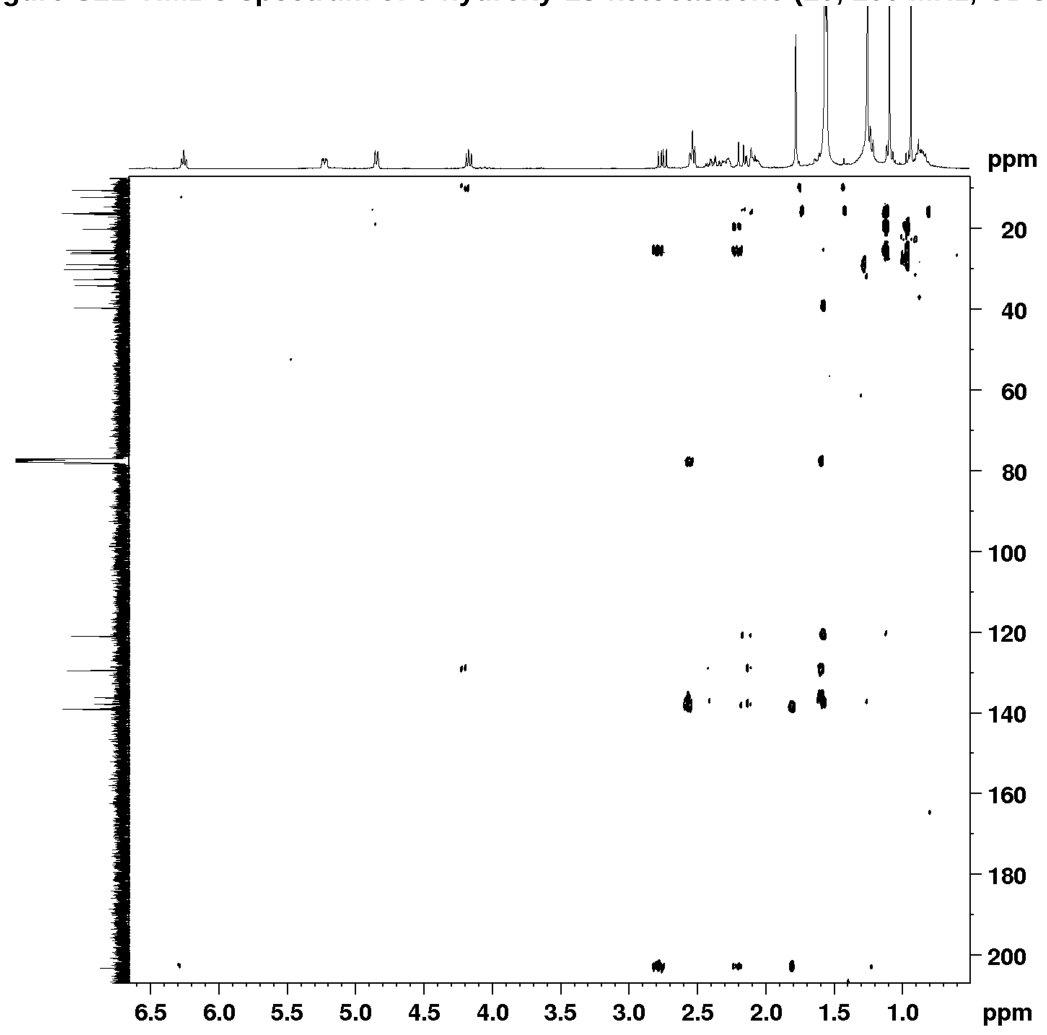

Figure S22. ^{1}H NMR spectrum of compound 12 (400 MHz, $CDCl_3$)

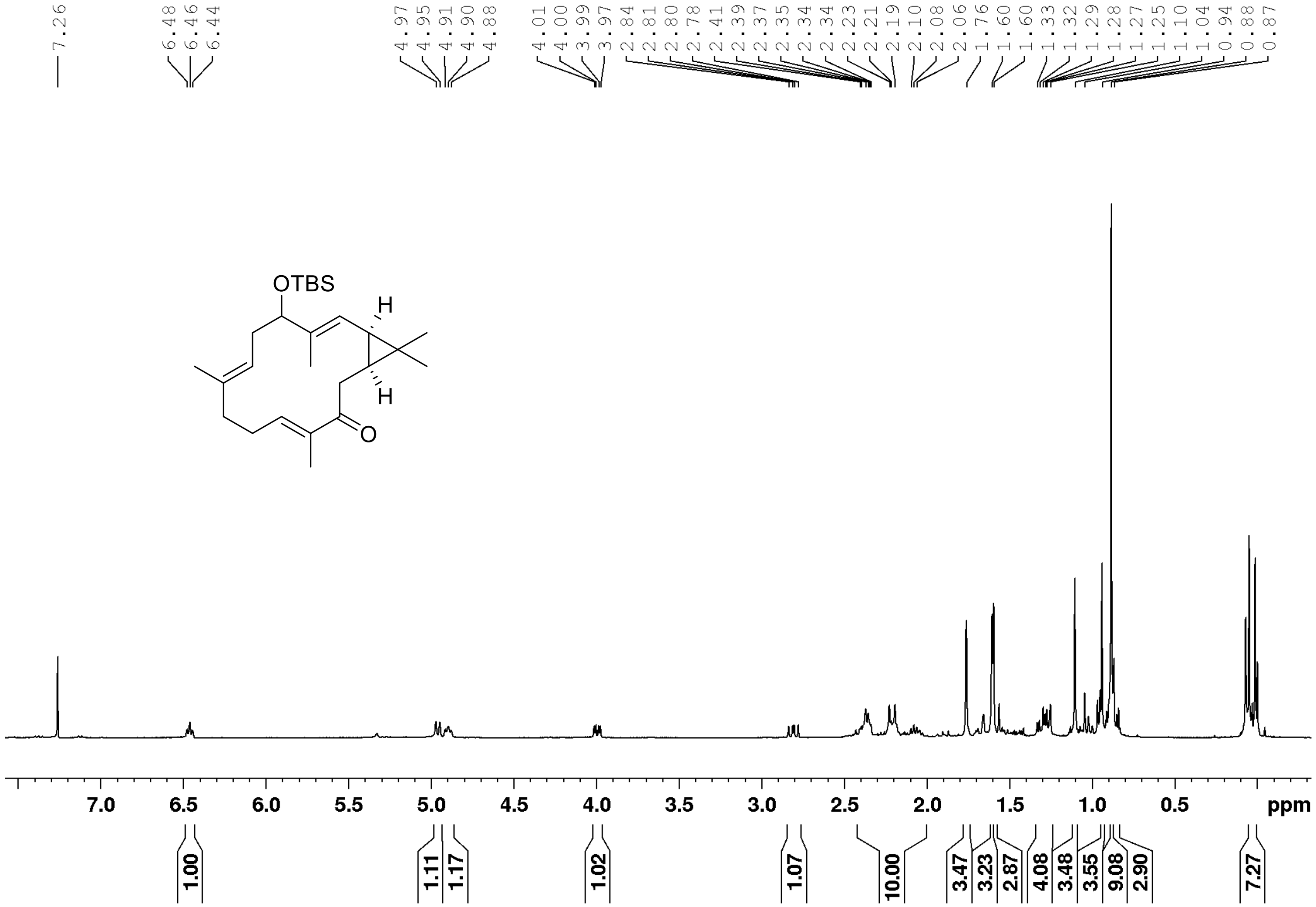

Figure S23. ^{13}C NMR spectrum of compound 12 (100 MHz, $CDCl_3$)

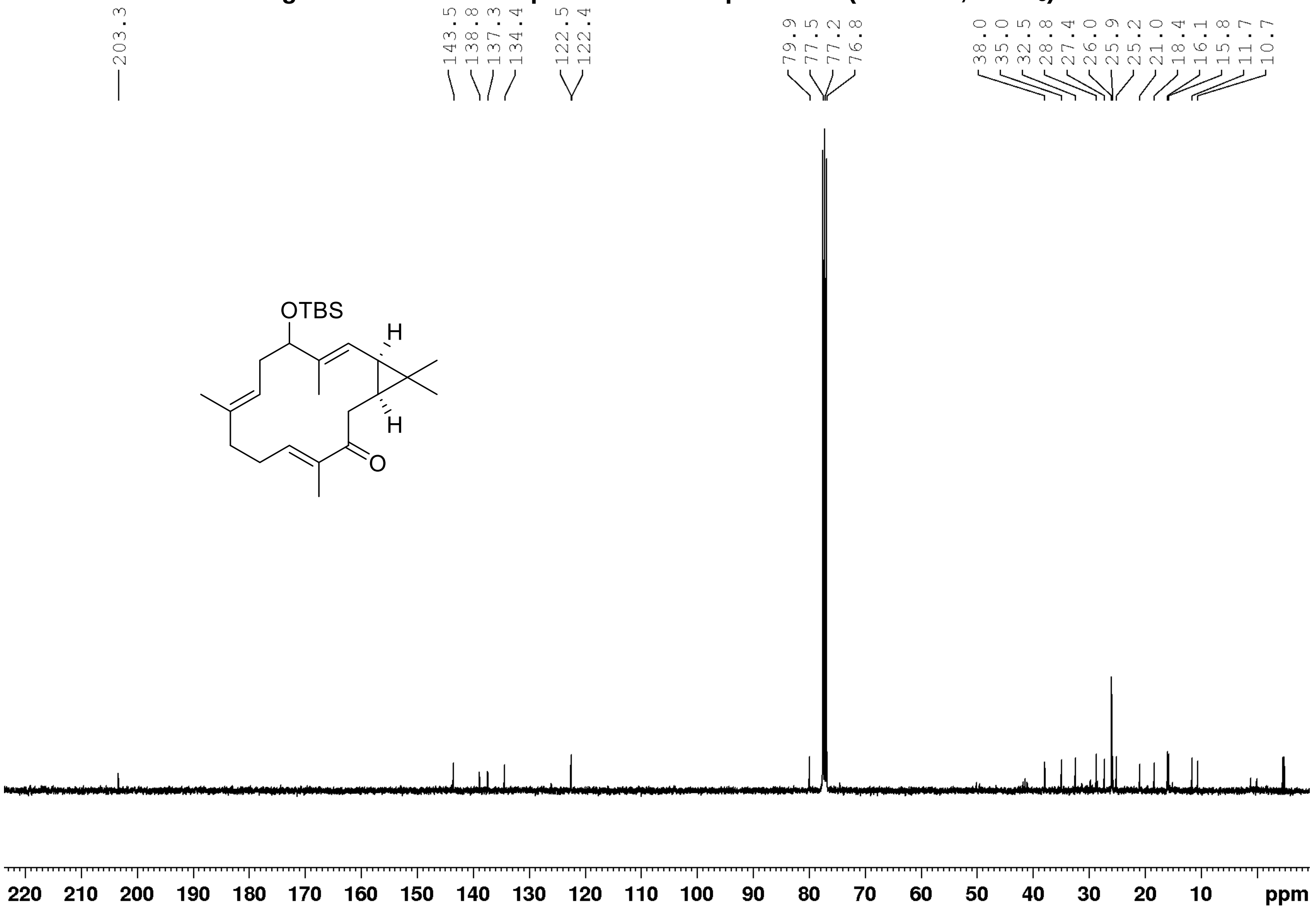

Figure S24. ^{1}H **NMR spectrum of compounds 13a/b (dr = 5:3) (400 MHz, CDCl$_3$)**

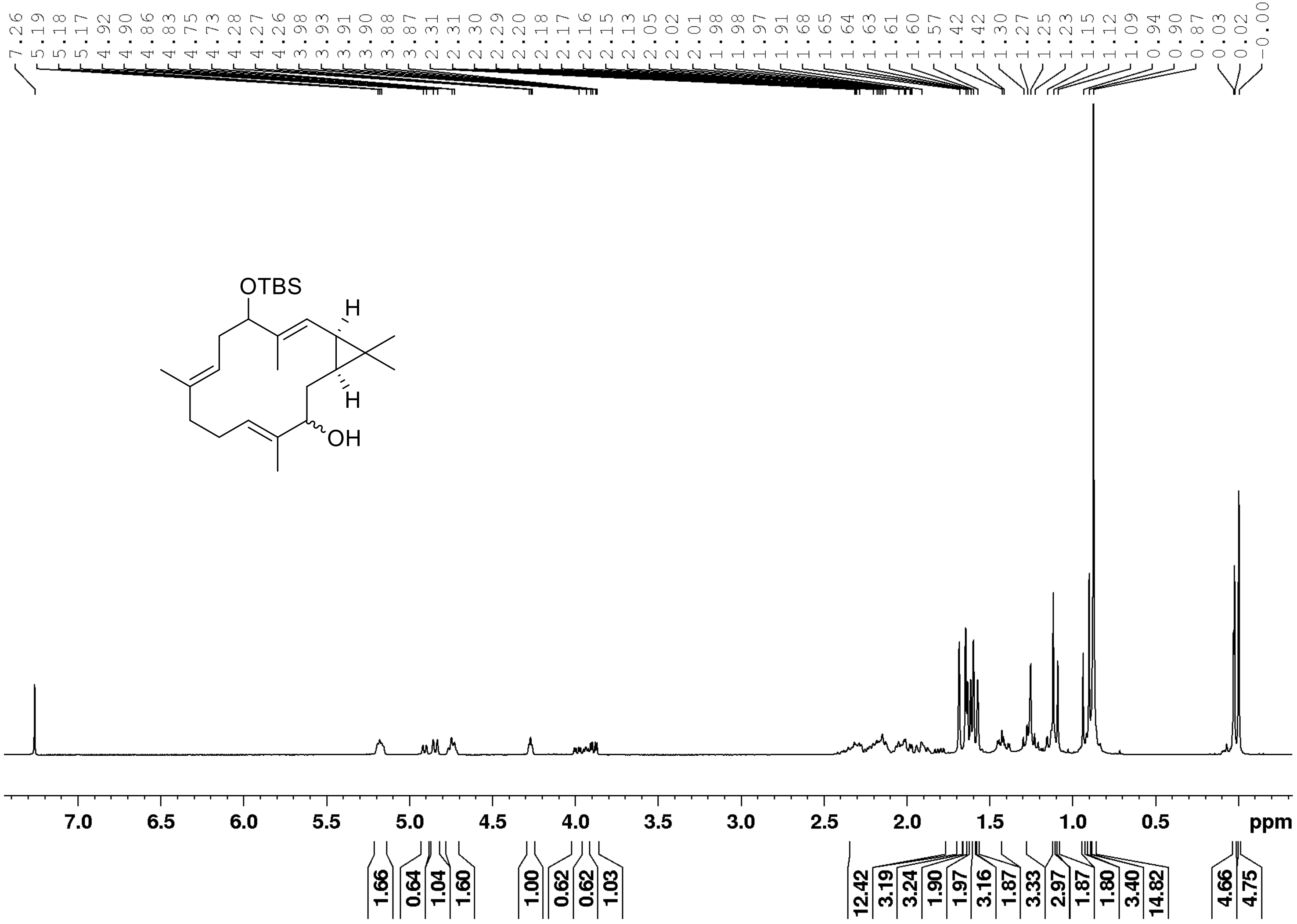

Figure S25. ^{13}C NMR spectrum of compounds 13a/b (dr = 5:3) (100 MHz, $CDCl_3$)

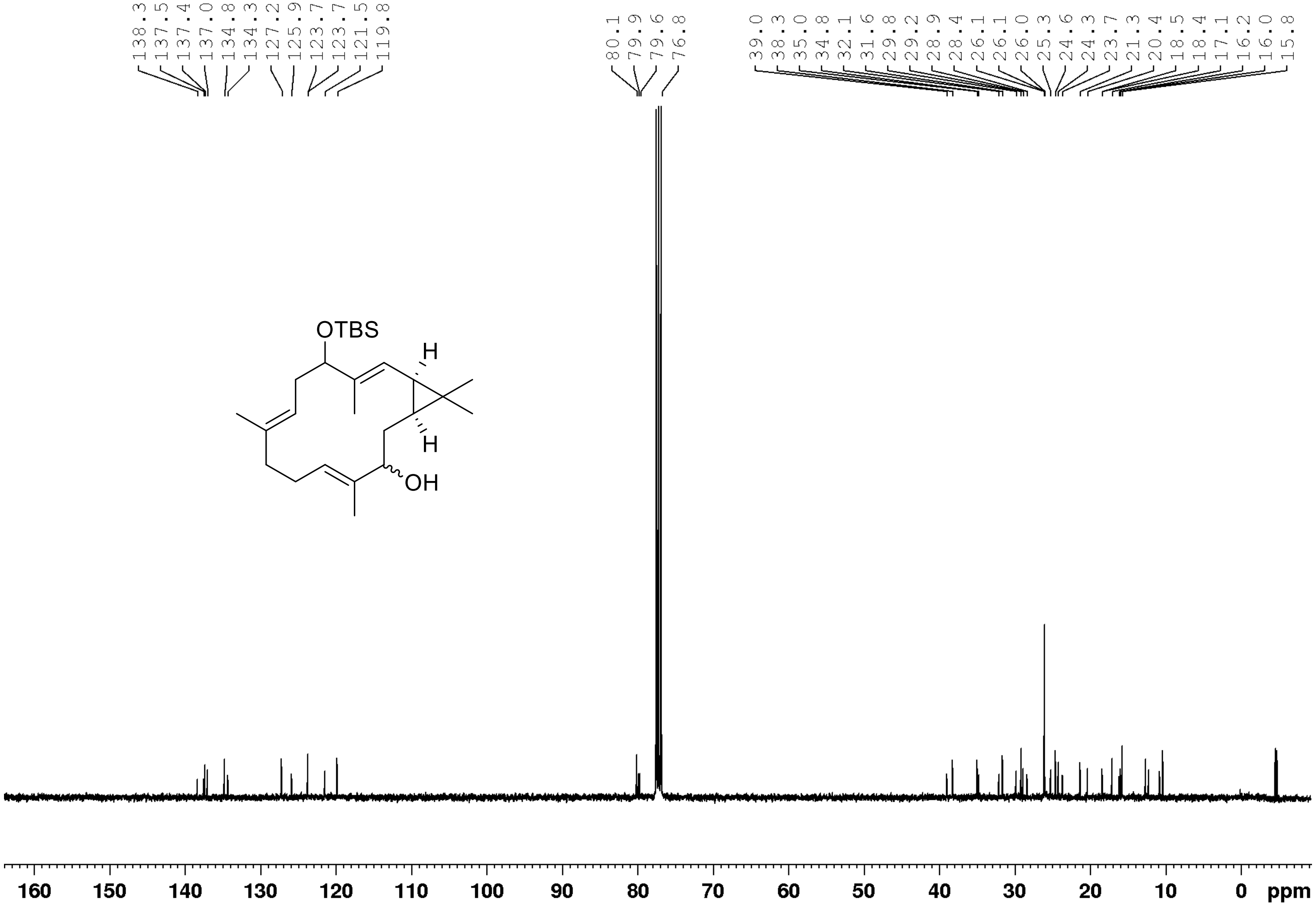

Figure S26. ^{1}H NMR spectrum of depressin (1, 600 MHz, $CDCl_3$)

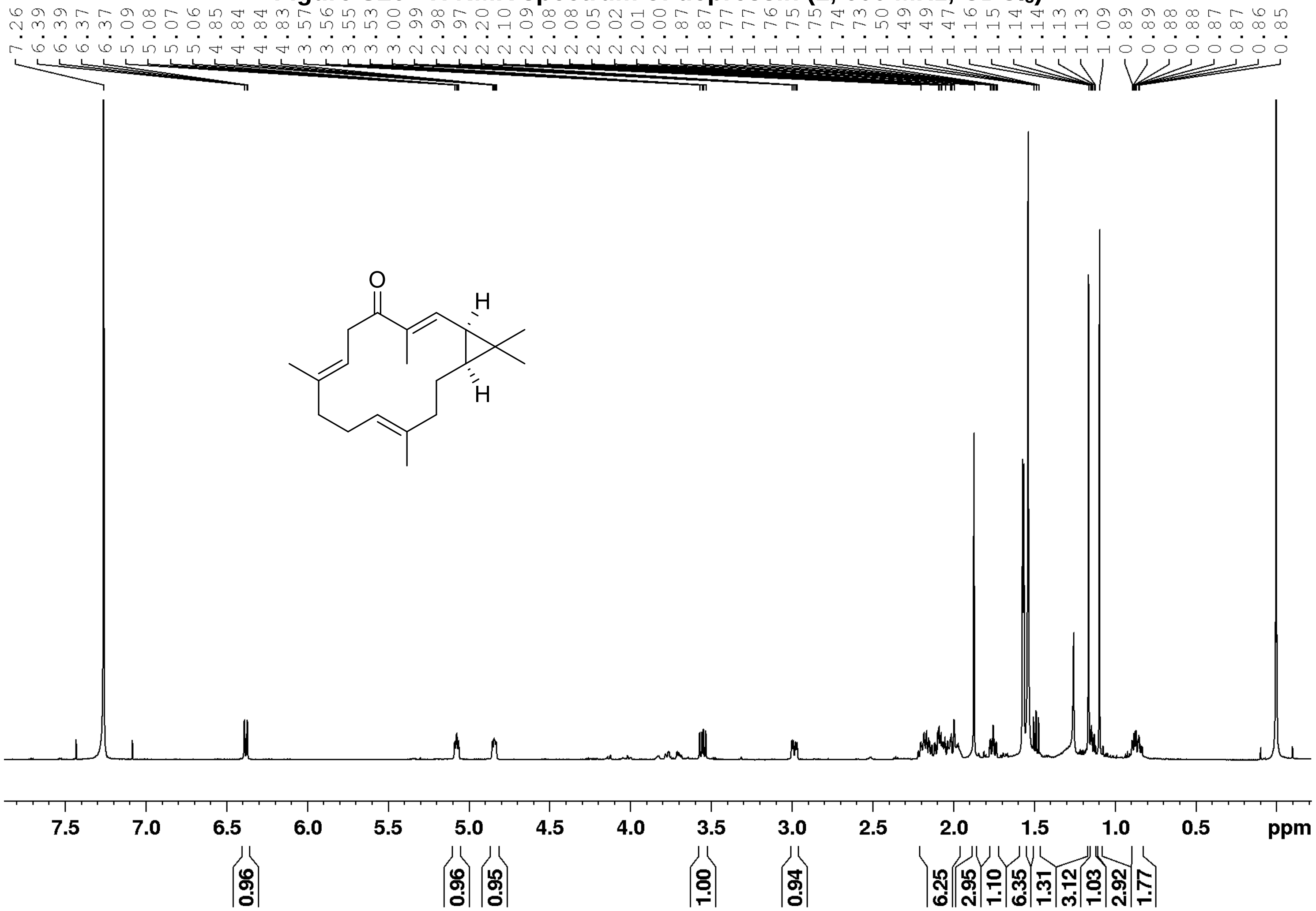

Figure S27. ^{13}C NMR spectrum of depressin (1, 150 MHz, $CDCl_3$)

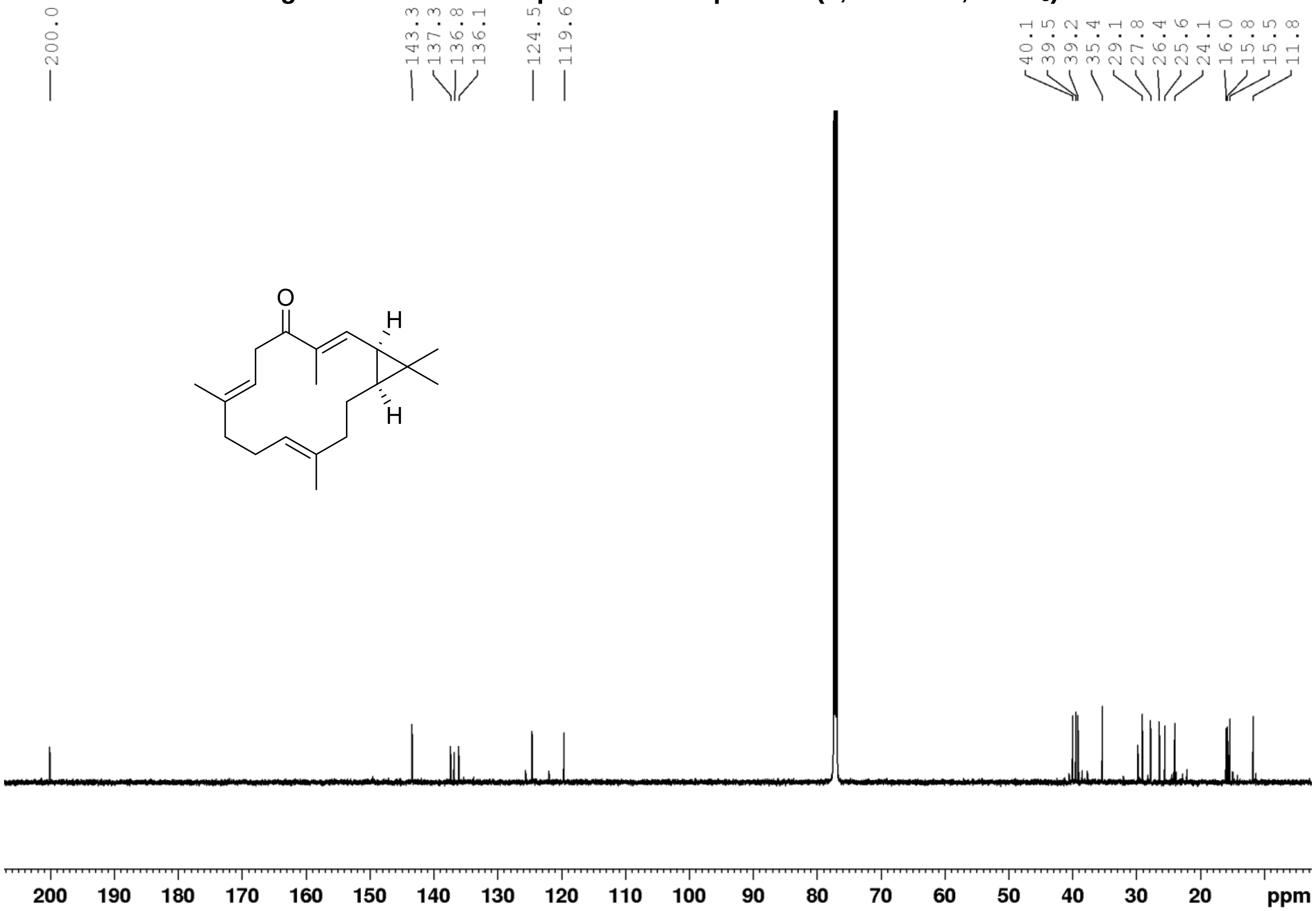

Figure S28. ^1H **NMR spectrum of compound 15 (600 MHz, $CDCl_3$)**

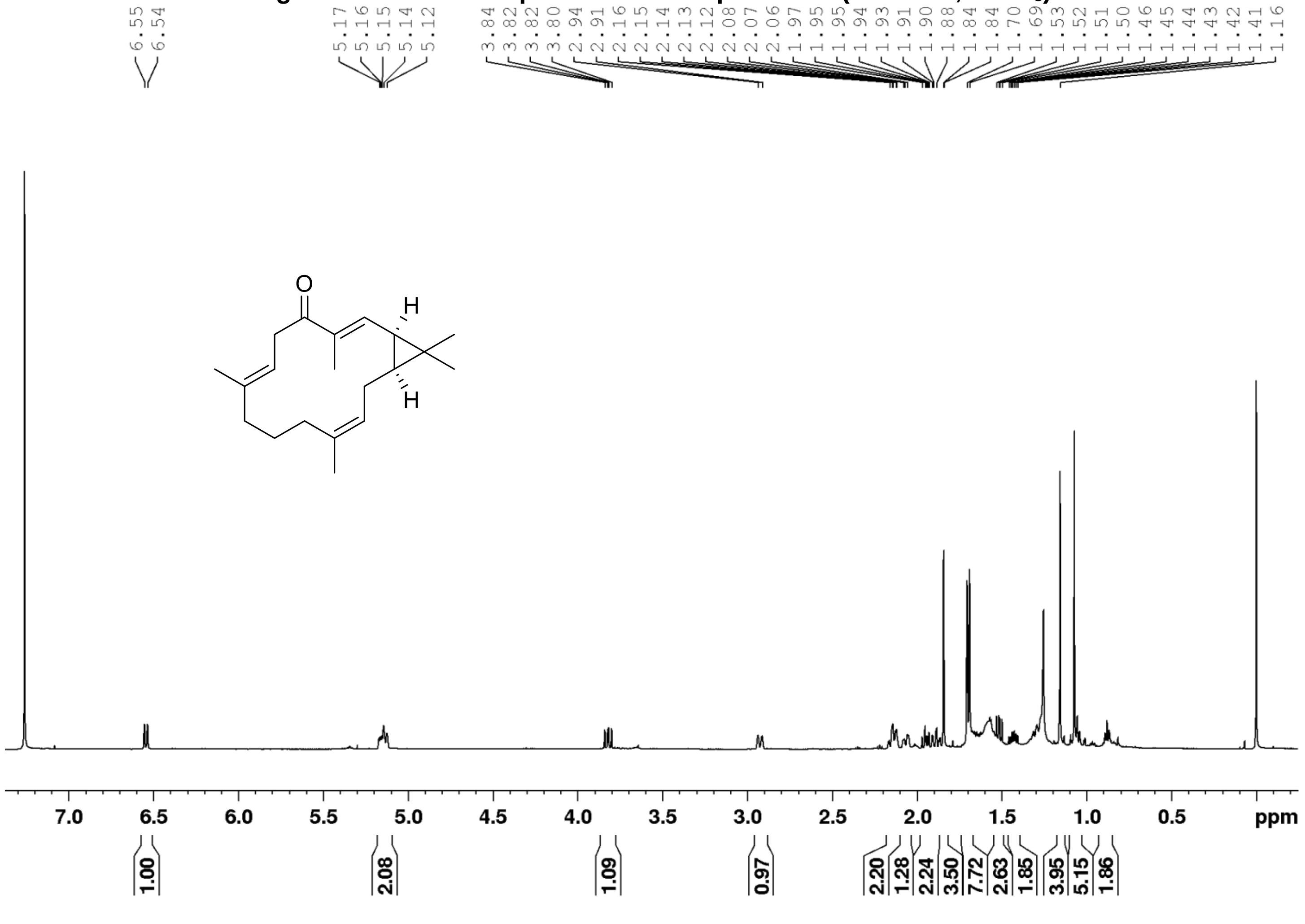

Figure S29. ^{13}C NMR spectrum of compound 15 (150 MHz, $CDCl_3$)

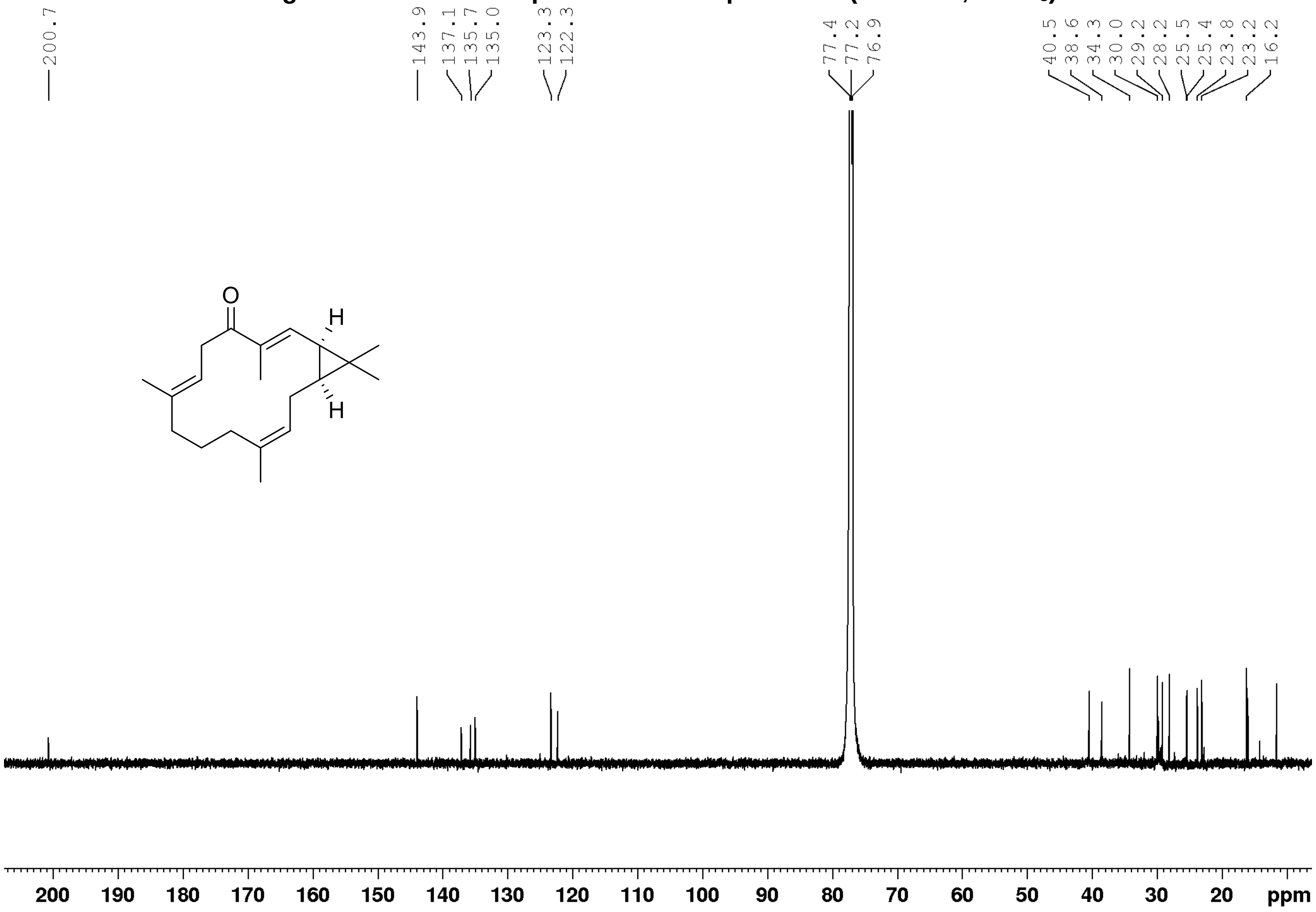

Figure S30. ^1H-^1H COSY spectrum of compound 15 (600 MHz, $CDCl_3$)

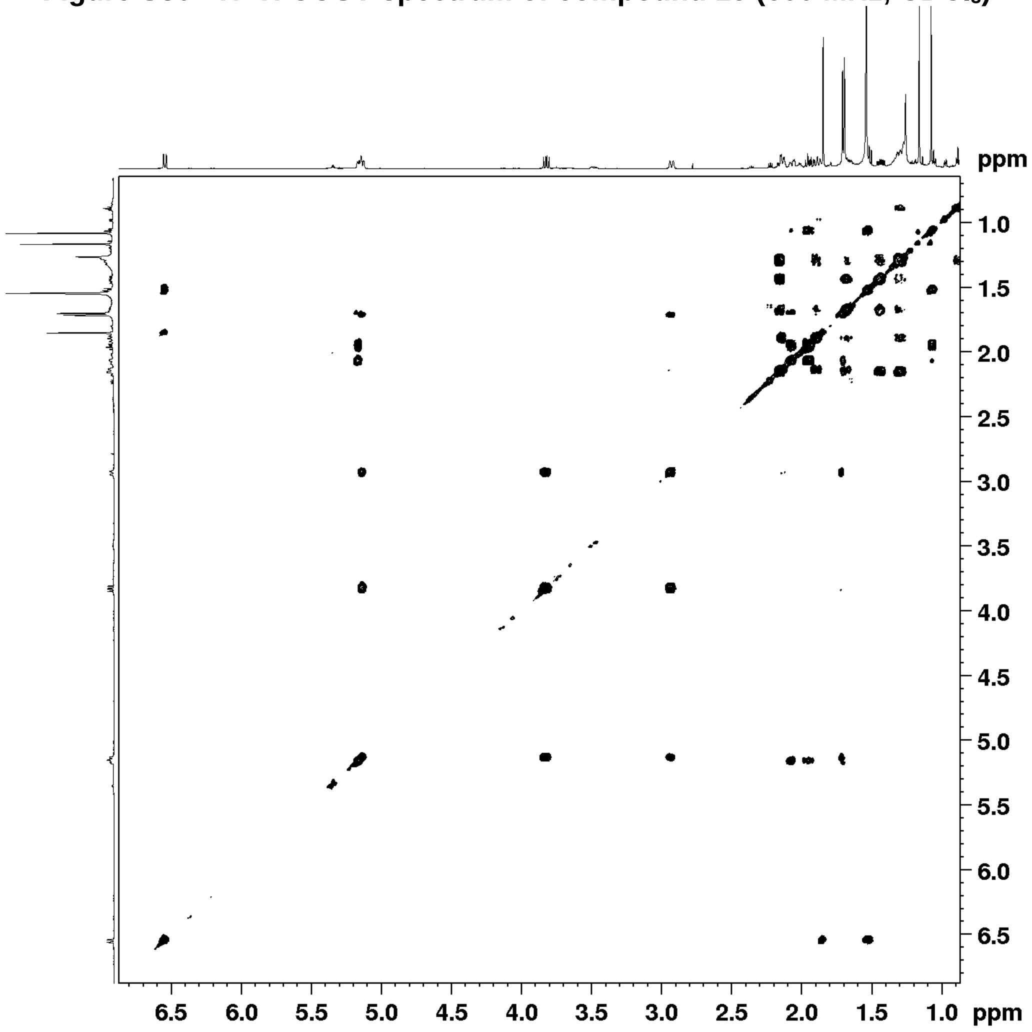

Figure S31. NOESY spectrum of compound 15 (600 MHz, $CDCl_3$)

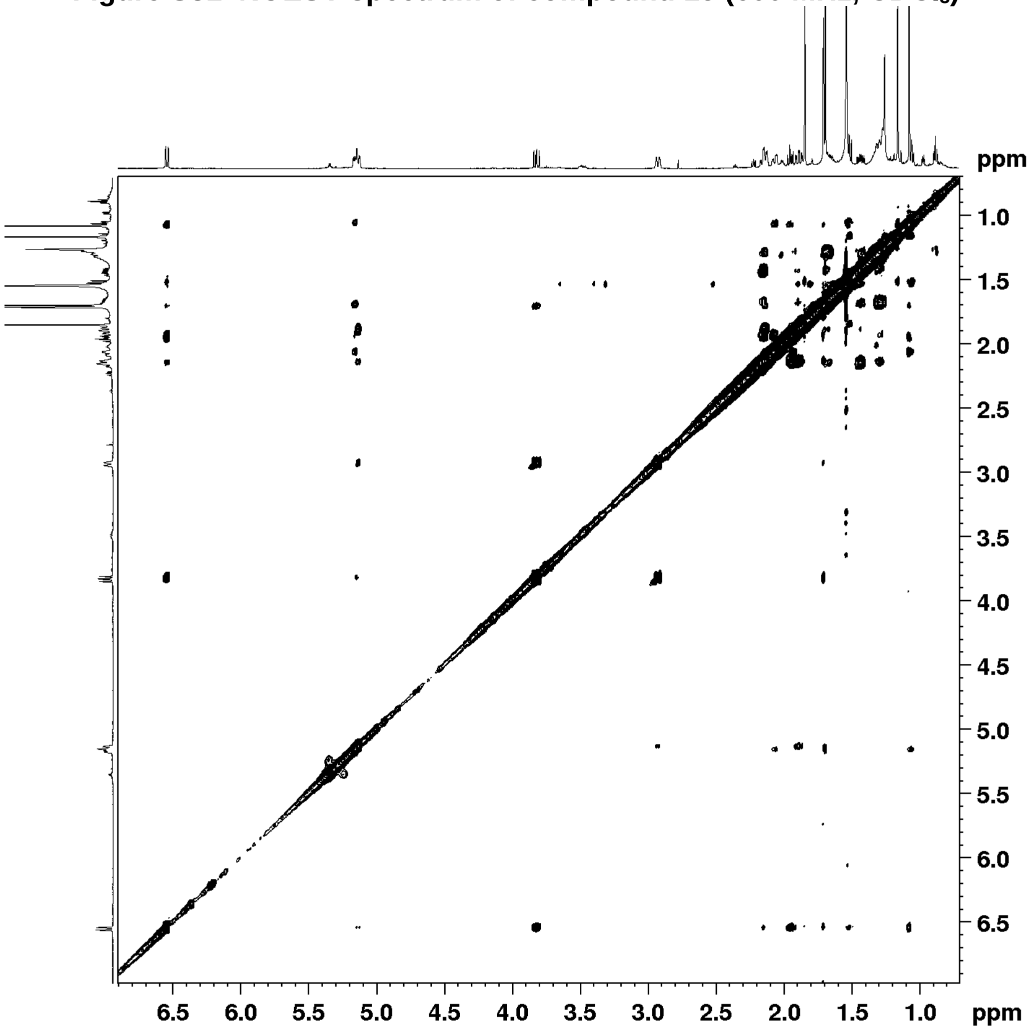

Figure S32. HSQC spectrum of compound 15 (150 MHz, $CDCl_3$)

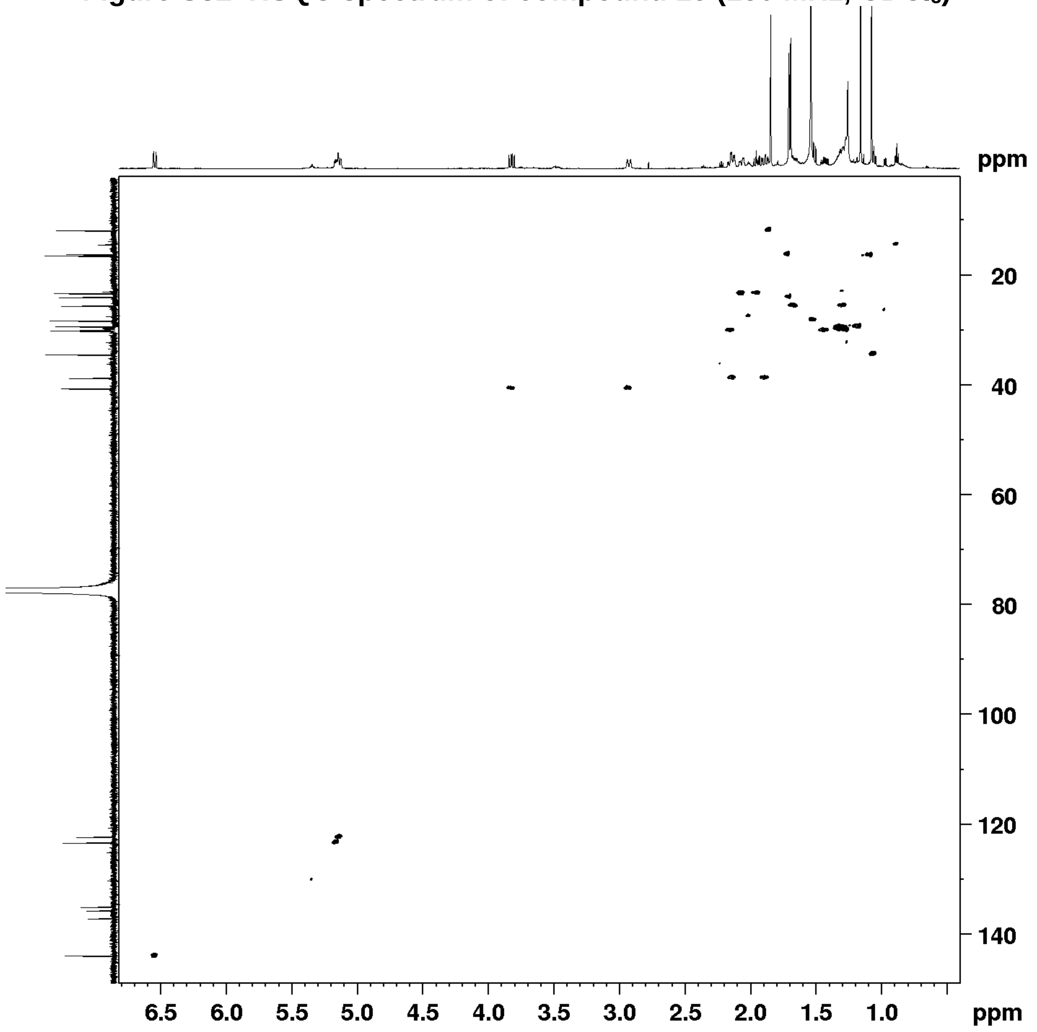

Figure S33. HMBC spectrum of compound 15 (150 MHz, $CDCl_3$)

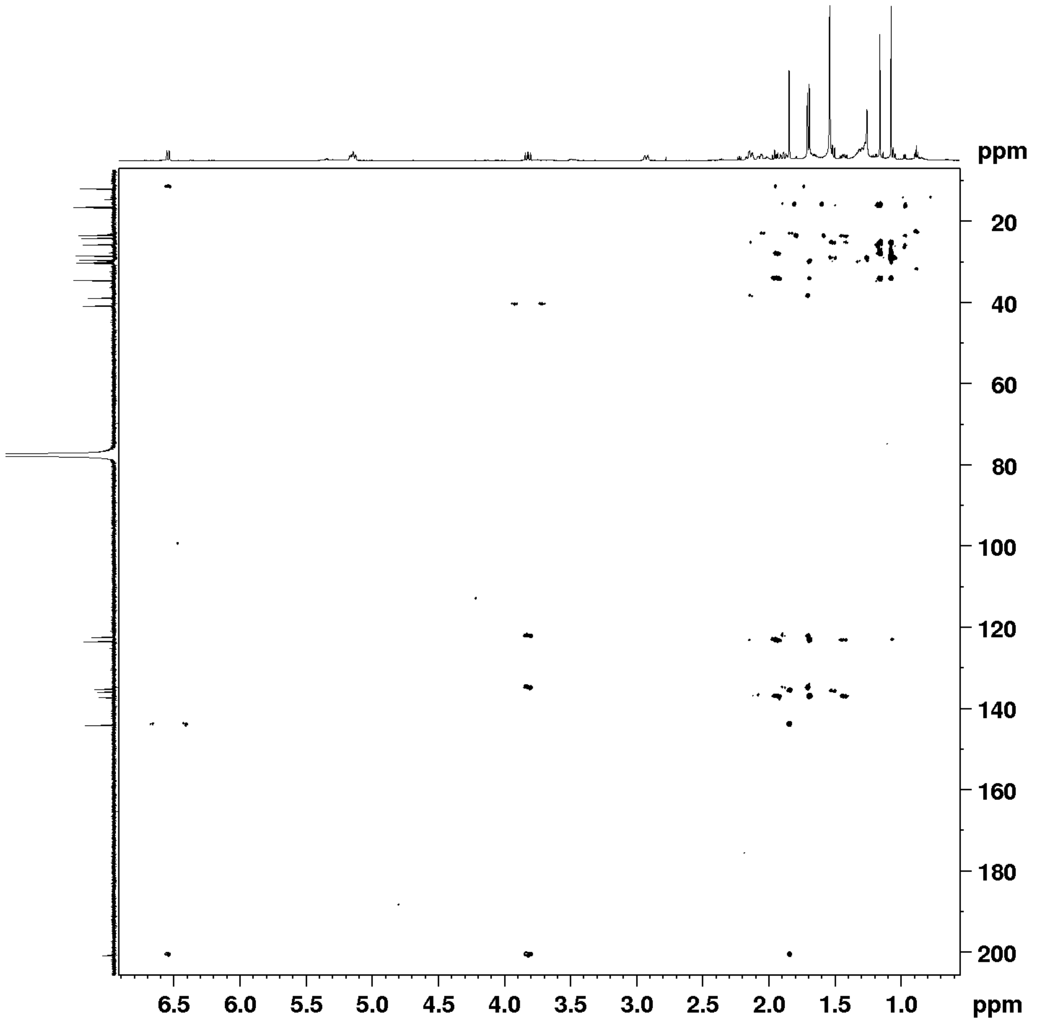

Figure S34. ^{1}H NMR spectrum of germacrene A (5, 400 MHz, $CDCl_3$)

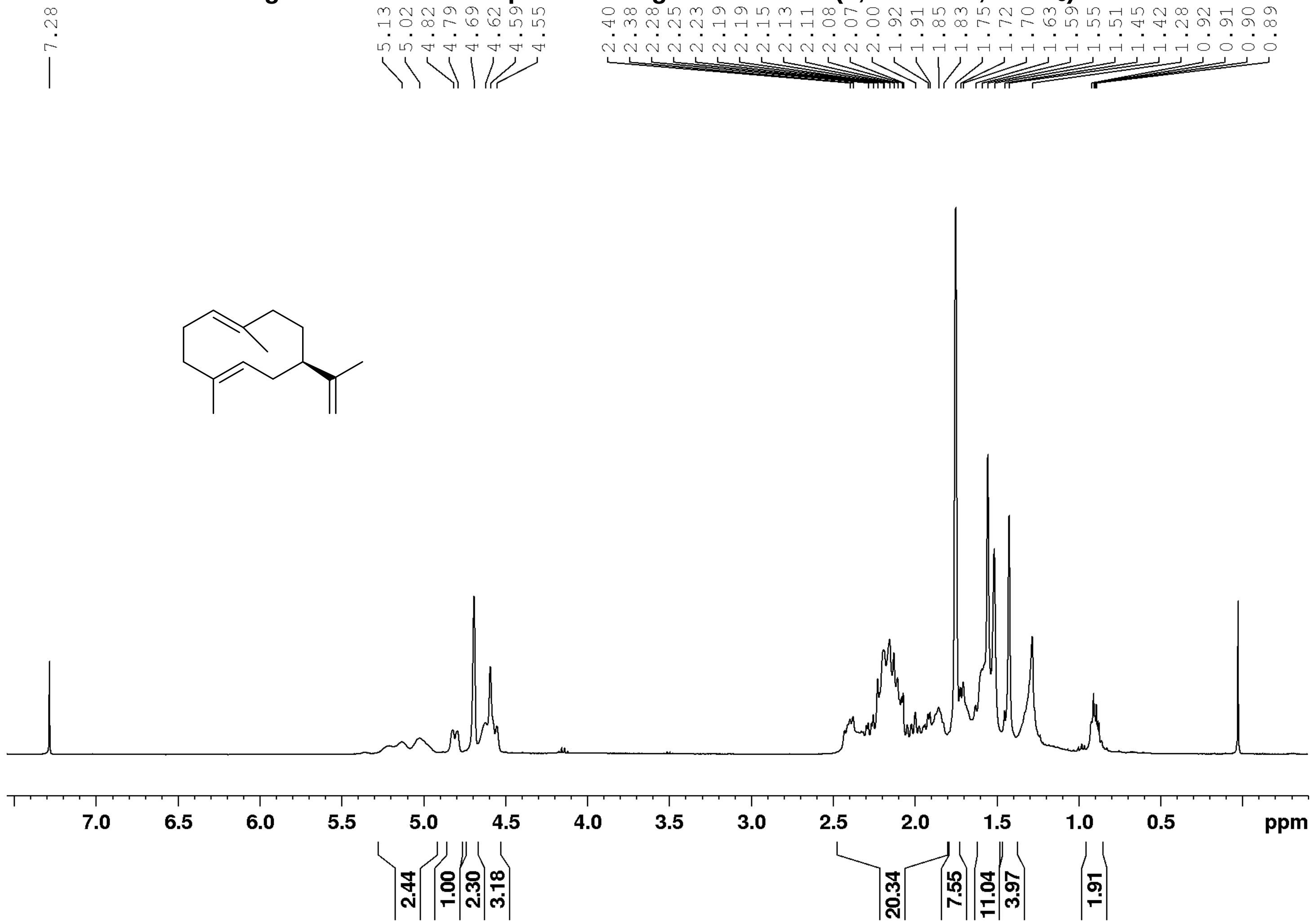

Figure S35. ^{13}C NMR spectrum of germacrene A (5, 100 MHz, $CDCl_3$)

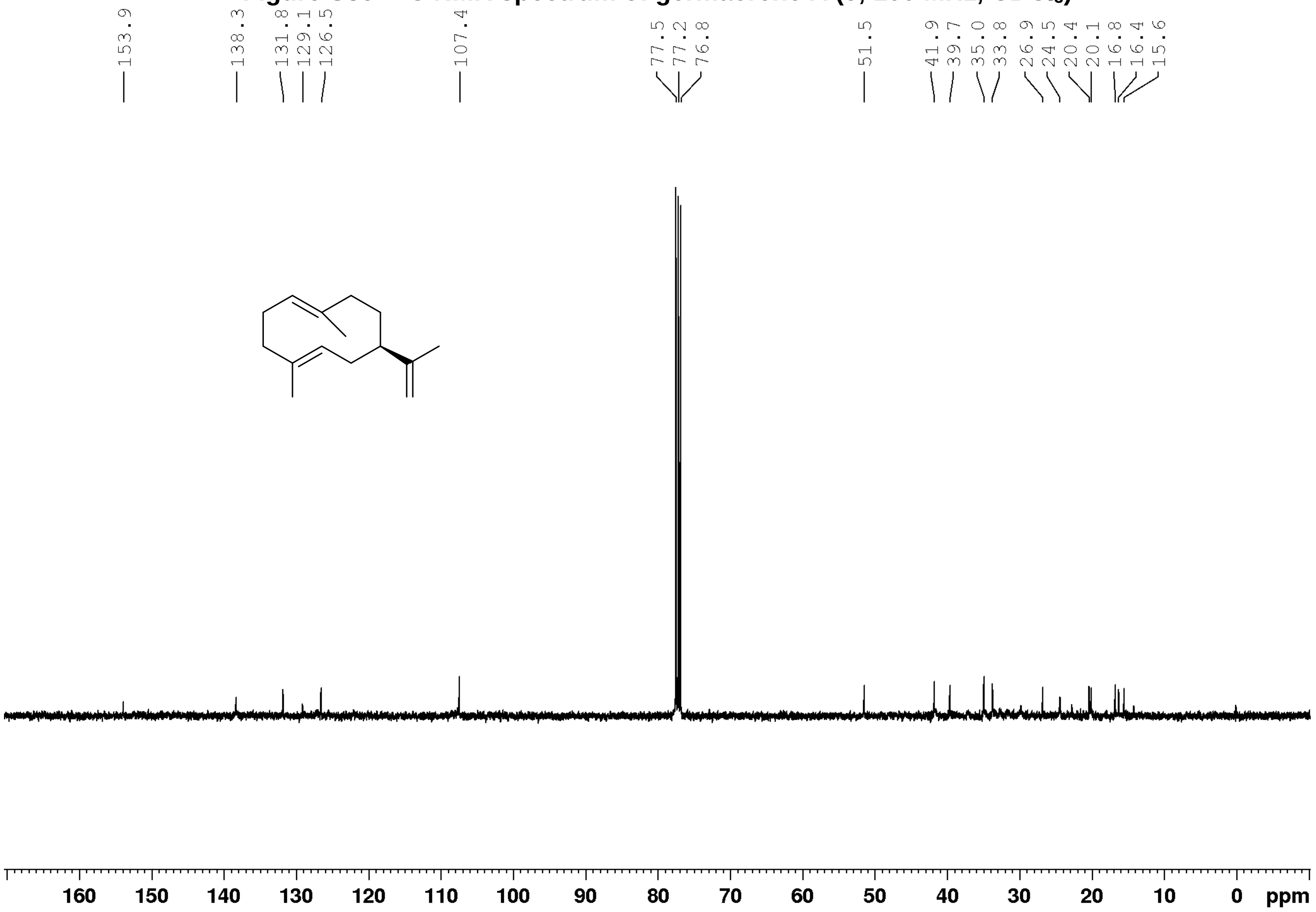

Figure S36. ^{1}H NMR spectrum of 4-*epi*-cryptomeridiol (3, 400 MHz, $CDCl_3$)

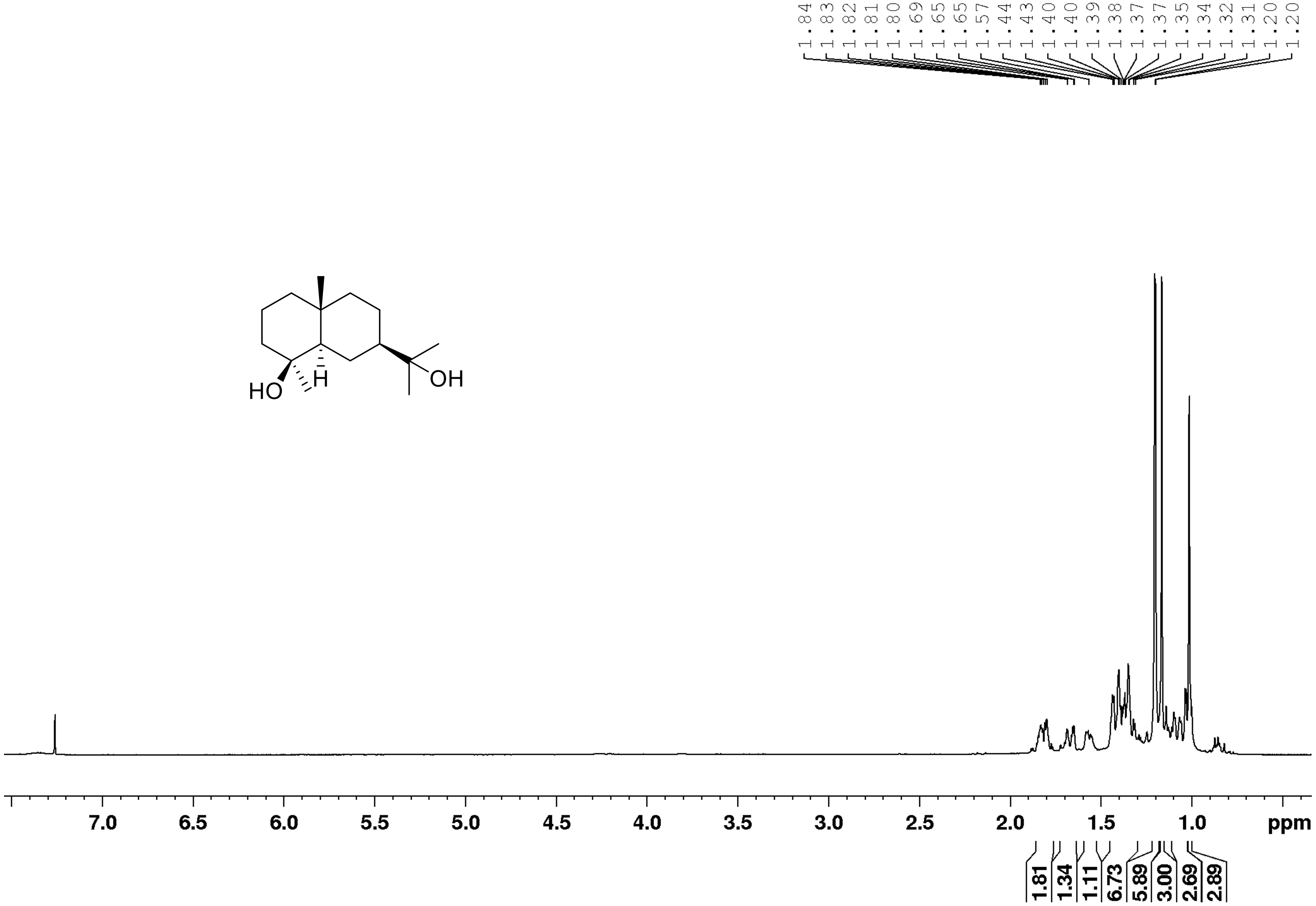

Figure S37. ^{13}**C NMR spectrum of 4-*epi*-cryptomeridiol (3, 100 MHz, $CDCl_3$)**

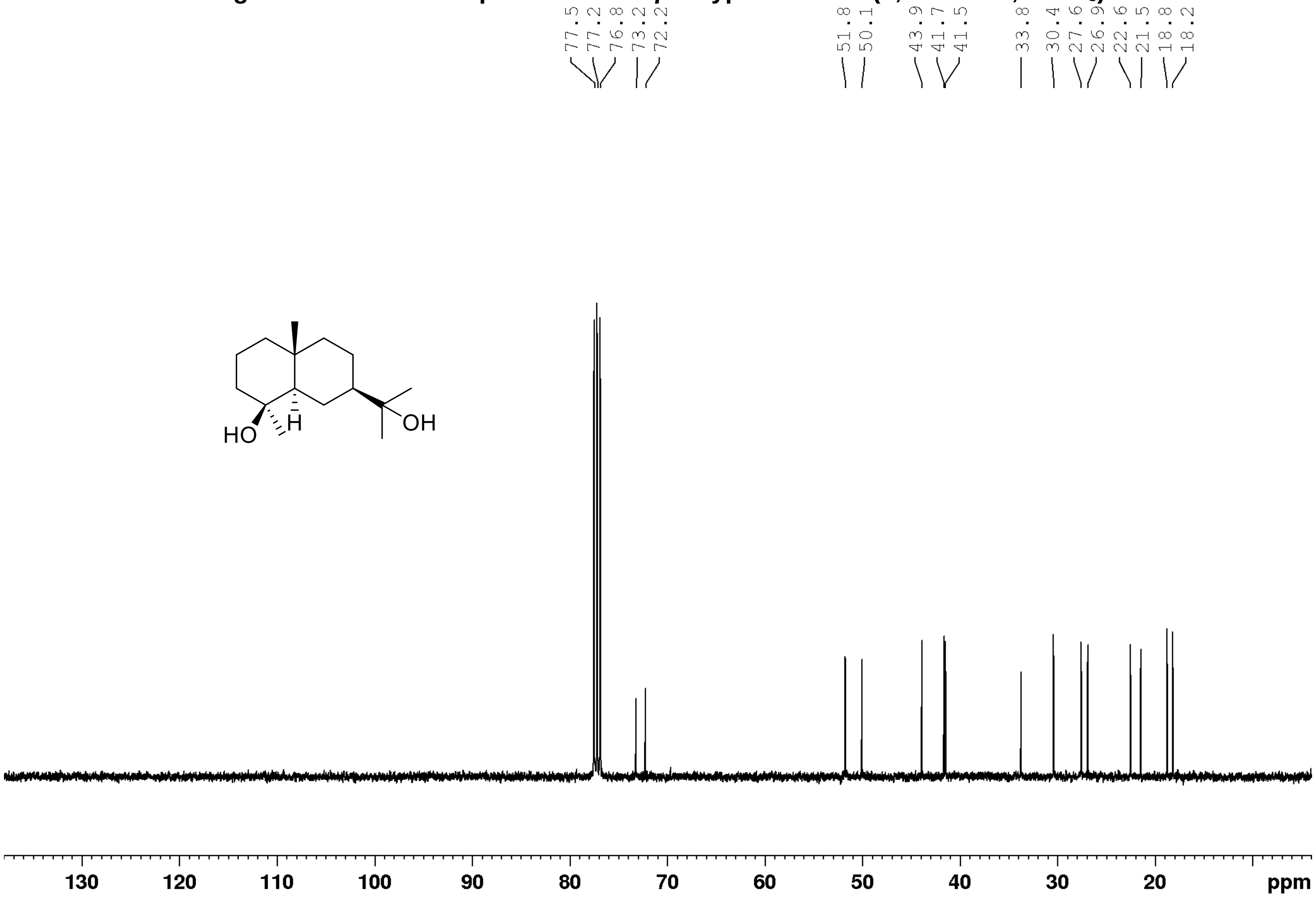

Figure S38. ^{1}H NMR spectrum of cryptomeridiol (2, 400 MHz, $CDCl_3$)

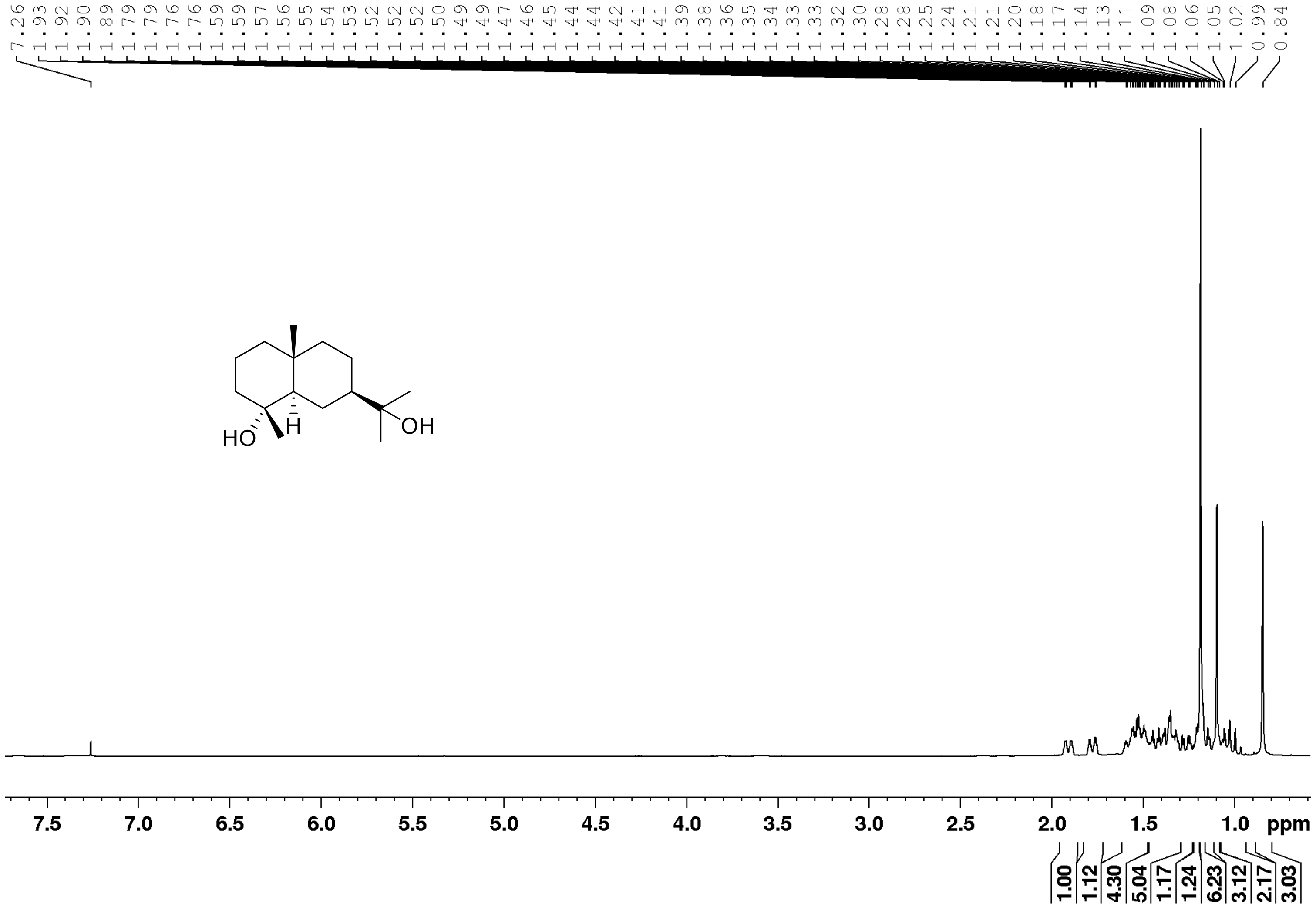

Figure S39. ^{13}C NMR spectrum of cryptomeridiol (2, 100 MHz, $CDCl_3$)

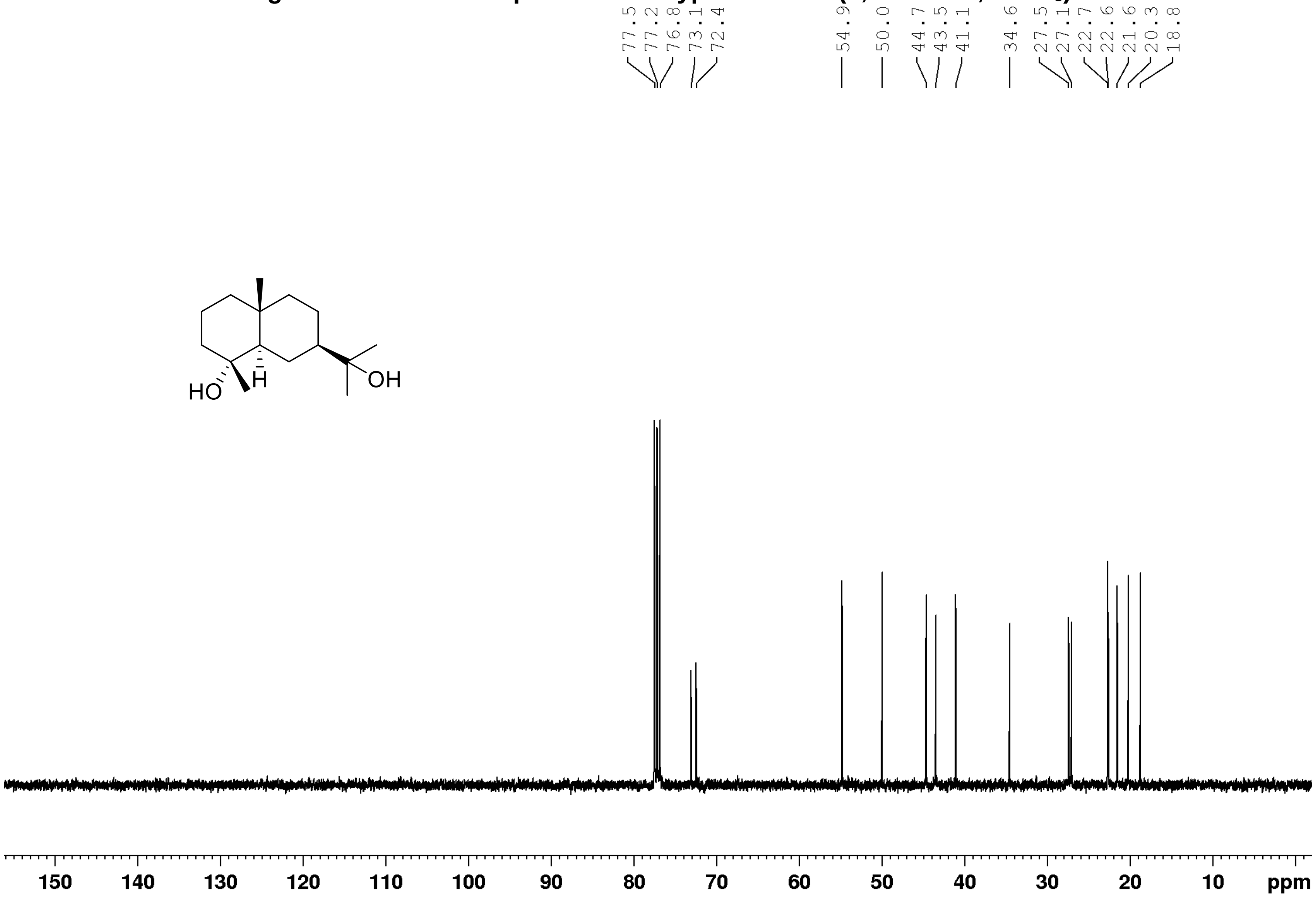

References

1. Faraldos, J. A.; Wu, S.; Chappell, J.; Coates, R. M. Conformational analysis of (+)-germacrene A by variable-temperature NMR and NOE spectroscopy. *Tetrahedron* **2007**, *63*, 7733-7742.
2. McMurry, J. E.; Bosch, G. K. Synthesis of macrocyclic terpenoid hydrocarbons by intramolecular carbonyl coupling: bicyclogermacrene, lepidozene, and casbene. *J. Org. Chem.* **1987**, *52*, 4885-4893.
3. Löffler, L. E.; Wirtz, C.; Fürstner, A. Collective total synthesis of casbane diterpenes: one strategy, multiple targets. *Angew. Chem. Int. Ed.* **2021**, *60*, 5316-5322.
4. Ando, M.; Arai, K.; Kikuchi, K.; Isogai, K. Synthetic Studies of Sesquiterpenes with a cis-Fused Decalin System, 4. Synthesis of (+)-5βH-Eudesma-3,11-diene, (-)-5βH-Eudesmane-4β,11-diol, and (+)-5βH-Eudesmane-4α,11-diol, and Structure Revision of a Natural Eudesmane-4,11-diol Isolated from *Pluchea arguta*. *J. Nat. Prod.* **1994,** *57*, 1189-1199.
5. Zhang, S.; Wang, K.; Liu, Y.; Wang, T.; Kong, Y.; Zhang, P.; Zhang, B.; Yin, M.; Pan, G.; Xu, Z. An isopentenol utilization pathway-based “deuterium-scanning” method for mechanistic investigations of terpene cyclases. *ACS Catal.* **2024**, *14*, 17598-17608.
6. Wang, T.; Zou, J.; Wang, K.; Liu, Y.; Zhang, S.; Kong, Y.; Xu, Z. Chemoenzymatic synthesis of the cyclopiane family of diterpenoid natural products. *Angew. Chem. Int. Ed.* **2025**, *64*, e202419092.
7. Marcos, I. S.; Hernández, F. A.; Sexmero, M. J.; Díez, D.; Basabe, P.; Pedrero, A. B.; García, N.; Urones, J. G. Synthesis and absolute configuration of (-)-Chettaphanin I and (-)-Chettaphanin II. *Tetrahedron* **2003**, *59*, 685-694.
8. Jiang, B.; Shi, H.-P.; Tian, W.-S.; Zhou, W.-S. The Convergent synthesis of novel cytotoxic certonardosterol D_2 from diosgenin. *Tetrahedron* **2008**, 64, 469-476.
9. Mukaiyama, T.; Shiina, I.; Iwadare, H.; Saitoh, M.; Nishimura, T.; Ohkawa, N.; Sakoh, H.; Nishimura, K.; Tani, Y.-i.; Hasegawa, M.; Yamada, K.; Saitoh, K. Asymmetric total Synthesis of Taxol. *Chem. Eur. J.* **1999**, 5, 121-161.
10. Albert, B. J.; Sivaramakrishnan, A.; Naka, T.; Czaicki, N. L.; Koide, K. Total syntheses, fragmentation studies, and antitumor/antiproliferative activities of FR901464 and its low picomolar analogue. *J. Am. Chem. Soc.* **2007**, 129, 2648-2659.
11. Shing, T. K. M.; Jiang, Q. Total synthesis of (+)-Quassin. *J. Org. Chem.* **2000**, 65, 7059-7069.
12. Mori, K.; Takaishi, H. Synthesis of Mono- and Sesquiterpenoids, XVI Synthesis of (-)-Pereniporins A and B, Sesquiterpene Antibiotics from a Basidiomycete. *Liebigs Ann. Chem.* **1989**, *1989*, 939-943.
13. Liu, C.; Cui, X.; Chen, W.; Ma, X.; Prather, K. J.; Zhou, K.; Wu, J. Synthesis of oxygenated sesquiterpenoids enabled by combining metabolic engineering and Visible-Light photocatalysis. *Chem. Eur. J.* **2022**, 28, e202201230.
14. Schultz, A. G.; Wang, A. First asymmetric synthesis of a Hasubanan Alkaloid. Total synthesis of (+)-Cepharamine. *J. Am. Chem. Soc.* **1998**, 120, 8259-8260.
15. Ho, T. L.; Su, C. Y. Total synthesis of (±)-Nudenoic Acid. *J. Org. Chem.* **2000**, 65, 3566-3568.
16. Sparling, B. A.; Moebius, D. C.; Shair, M. D. Enantioselective total synthesis of Hyperforin. *J. Am. Chem. Soc.* **2013**, 135, 644-647.
17. Uwamori, M.; Saito, A.; Nakada, M. Stereoselective total synthesis of Nemorosone. *J. Org. Chem.* **2012**, 77, 5098-5107.
18. Toyota, M.; Asano, T.; Ihara, M. Total synthesis of Serofendic Acids A and B employing Tin-free Homoallyl−Homoallyl radical rearrangement. *Org. Lett.* **2005**, 7, 3929-3932.
19. Soorukram, D.; Qu, T.; Barrett, A. G. M. Four-Component benzyne coupling reactions: A concise total synthesis of Dehydroaltenuene B. *Org. Lett.* **2008**, 10, 3833-3835.
20. Nicolaou, K. C.; Toh, Q. Y.; Chen, D. Y. K. An expedient asymmetric synthesis of Platencin. *J. Am. Chem. Soc.* **2008**, 130, 11292-11293.
21. Jung, M. E.; Johnson, T. W. First total synthesis of xestobergsterol A and active structural analogues of the xestobergsterols. *Tetrahedron*. **2001**, 57, 1449-1481.

22. Li, Y.; Carbone, M.; Vitale, R. M.; Amodeo, P.; Castelluccio, F.; Sicilia, G.; Mollo, E.; Nappo, M.; Cimino, G.; Guo, Y.-W.; Gavagnin, M. Rare casbane diterpenoids from the Hainan soft coral *Sinularia depressa*. *J. Nat. Prod.* **2010**, *73*, 133-138.
23. Löffler, L. E.; Wirtz, C.; Fürstner, A. Collective total synthesis of casbane diterpenes: one strategy, multiple targets. *Angew. Chem. Int. Ed.* **2021**, *60*, 5316-5322.
24. Kong, Y.; Liu, Y.; Wang, K.; Wang, T.; Wang, C.; Ai, B.; Jia, H.; Pan, G.; Yin, M.; Xu, Z. Confirmation of the stereochemistry of spiroviolene. *Beiltein J. Org. Chem.* **2024**, *20*, 852-858.
